# Cluster Tails for Critical Power-Law Inhomogeneous Random Graphs

**DOI:** 10.1007/s10955-018-1978-0

**Published:** 2018-03-03

**Authors:** Remco van der Hofstad, Sandra Kliem, Johan S. H. van Leeuwaarden

**Affiliations:** 1 Department of Mathematics and Computer Science, Eindhoven University of Technology, P.O. Box 513, 5600 MB Eindhoven, The Netherlands; 2 Fakultät für Mathematik, Universität Duisburg-Essen, Thea-Leymann-Str. 9, 45127 Essen, Germany

**Keywords:** Critical random graphs, Power-law degrees, Inhomogeneous networks, Thinned Lévy processes, Exponential tilting, Large deviations, 60C05, 05C80, 90B15

## Abstract

Recently, the scaling limit of cluster sizes for critical inhomogeneous random graphs of rank-1 type having finite variance but infinite third moment degrees was obtained in Bhamidi et al. (Ann Probab 40:2299–2361, 2012). It was proved that when the degrees obey a power law with exponent $\tau \in (3,4)$, the sequence of clusters ordered in decreasing size and multiplied through by $n^{-(\tau -2)/(\tau -1)}$ converges as $n\rightarrow \infty$ to a sequence of decreasing non-degenerate random variables. Here, we study the tails of the limit of the rescaled largest cluster, i.e., the probability that the scaling limit of the largest cluster takes a large value *u*, as a function of *u*. This extends a related result of Pittel (J Combin Theory Ser B 82(2):237–269, 2001) for the Erdős–Rényi random graph to the setting of rank-1 inhomogeneous random graphs with infinite third moment degrees. We make use of delicate large deviations and weak convergence arguments.

## Introduction

The Erdős–Rényi random graph *G*(*n*, *p*) on the vertex set $[n]:=\{1,\ldots ,n\}$ is constructed by including each of the ${n\atopwithdelims ()2}$ possible edges with probability *p*, independently of all other edges. Erdős and Rényi discovered the double-jump phenomenon: The size of the largest component was shown to be, in probability, of order $\log n$, $n^{2/3}$, or *n*, depending on whether the average vertex degree was less than, close to, or more than one. In 1984 Bollobás [10] and subsequently Łuczak [28] showed for the scaling window $p=(1+\lambda n^{-1/3})/n$, that the largest component is of the order $n^{2/3}$. Since then, the critical, or near-critical behavior of random graphs has received tremendous attention (see [2, 4, 9, 18, 27]). Let $({\mathcal {C}}_{\scriptscriptstyle (i)})_{i\ge 1}$ denote the connected components of *G*(*n*, *p*), ordered in size, i.e., $|{\mathcal {C}}_{\mathrm{max}}|=|{\mathcal {C}}_{\scriptscriptstyle (1)}|\ge |{\mathcal {C}}_{\scriptscriptstyle (2)}|\ge \cdots$ Aldous [2] proved the following result:

### Theorem 1.1

(Aldous [2]). For $p=(1+\lambda n^{-1/3})/n$, $\lambda \in \mathbb {R}$ fixed, and $n\rightarrow \infty$,

1.1 $$\begin{aligned} \left( |{\mathcal {C}}_{\scriptscriptstyle (1)}|n^{-2/3},|{\mathcal {C}}_{\scriptscriptstyle (2)}|n^{-2/3}, \ldots \right) {\mathop {\longrightarrow }\limits ^{d}}\left( \gamma _1(\lambda ),\gamma _2(\lambda ), \ldots \right) , \end{aligned}$$

where $\gamma _1(\lambda )>\gamma _2(\lambda )>\cdots$ are the ordered excursions of the reflected version of the process $(W^\lambda _t)_{t\ge 0} \equiv (W_t + \lambda t -t^2/2)_{t\ge 0}$ with $(W_t)_{t\ge 0}$ a standard Wiener process.

Theorem 1.1 says that the ordered connected components in the critical Erdős–Rényi random graph are described by the ordered excursions of the reflected version of $(W^\lambda _t)_{t\ge 0}$. The strict inequalities between the scaling limits of the ordered cluster follows from the local limit theorem proved in [23], see also [25, 29]. Pittel [31, Eq. (1.12)] derived an exact formula for the distribution function of the limiting variable $\gamma _1(\lambda )$ (of the largest component) and various asymptotic results were obtained, including

1.2 $$\begin{aligned} \mathbb {P}(\gamma _1(\lambda )>u)= \frac{1}{\sqrt{9\pi /8}u^{3/2}}{\mathrm {e}}^{-\frac{1}{8}u(u-2\lambda )^2}(1+o(1)), \quad u\rightarrow \infty . \end{aligned}$$

As pointed out in [32, 33], the constant $\sqrt{9\pi /8}$ was mistakenly reported in [31, Eq. (1.12)] as $\sqrt{2\pi }$ due to a small oversight in the derivation. The result in (1.2) gives sharp asymptotics for the largest component in the critical Erdős–Rényi graph. It was rederived and extended in [33] using the original approach in [31]. Another generalization of (1.2) was obtained in [24] by studying the excursions of the scaling limit of the exploration process that is used to describe the limits in Theorem 1.1. In this paper, we follow a similar path, but then for a class of inhomogeneous random graphs and its scaling limit, and extend (1.2) to this setting.

Several recent works have studied inhomogeneity in random graphs and how it changes the critical nature. In our model, the vertices have a weight associated to them, and the weight of a vertex moderates its degree. Therefore, by choosing these weights appropriately, we can generate random graphs with highly variable degrees. For our class of random graphs, it is shown in [22, Theorem 1.1] that when the weights do not vary too much, the critical behavior is similar to the one in the Erdős–Rényi random graph. See in particular the recent works [6, 34], where it was shown that if the degrees have finite *third* moment, then the scaling limit for the largest critical components in the critical window are essentially the same (up to a trivial rescaling that we explain in more detail below) as for the Erdős–Rényi random graph in Theorem 1.1.

When the degrees have *infinite* third moment, instead, it was shown in [22, Theorem 1.2] that the sizes of the largest critical clusters are quite different. In [7] scaling limits were obtained for the sizes of the largest components at criticality for rank-1 inhomogeneous random graphs with power-law degrees with power-law exponent $\tau \in (3,4)$. For $\tau \in (3,4)$, the degrees have finite variance but infinite third moment. It was shown that the sizes of the largest components, rescaled by $n^{-(\tau -2)/(\tau -1)}$, converge to hitting times of a *thinned Lévy process*. The latter is a special case of the general multiplicative coalescents studied by Aldous and Limic in [2] and [3]. We next discuss these results in more detail.

### Inhomogeneous Random Graphs

In our random graph model, vertices have weights, and the edges are independent, with edge probabilities being approximately equal to the rescaled product of the weights of the two end vertices of the edge. While there are many different versions of such random graphs (see below), it will be convenient for us to work with the so-called Poissonian random graph or Norros–Reittu model [30]. To define the model, we consider the vertex set $[n]:=\{1,2,\ldots , n\}$ and suppose each vertex is assigned a weight, vertex *i* having weight $w_i$. Now, attach an edge between vertices *i* and *j* with probability

1.3 $$\begin{aligned} p_{ij} = 1-\mathrm{exp}\Big (-\frac{w_i w_j}{\ell _n}\Big ), \quad \text{ where }\quad \ell _n=\sum _{i\in [n]} w_i. \end{aligned}$$

Different edges are independent. In this model, the average degree of vertex *i* is close to $w_i$, thus incorporating inhomogeneity in the model.

There are many adaptations of this model, for which equivalent results hold. Indeed, the model considered here is a special case of the so-called *rank-1 inhomogeneous random graph* introduced in great generality by Bollobás et al. [11]. It is asymptotically equivalent with many related models, such as the *random graph with prescribed expected degrees* or Chung-Lu model, where instead

1.4 $$\begin{aligned} p_{ij}=\max (w_iw_j/\ell _n, 1), \end{aligned}$$

and which has been studied intensively by Chung and Lu (see [13–17]). A further adaptation is the *generalized random graph* introduced by Britton et al. [12], for which

1.5 $$\begin{aligned} p_{ij} = \frac{w_i w_j}{\ell _n+w_iw_j}. \end{aligned}$$

See Janson [26] for conditions under which these random graphs are *asymptotically equivalent*, meaning that all events have asymptotically equal probabilities. As discussed in more detail in [22, Sect. 1.3], these conditions apply in the setting to be studied in this paper. Therefore, all results proved here also hold for these related rank-1 models. We refer the interested reader to [22, Sect. 1.3] for more details.

Having specified the edge probabilities as functions of the vertex weights $\varvec{w}= (w_i)_{i\in [n]}$ in (1.3), we now explain how we choose the vertex weights. Let the weight sequence $\varvec{w}= (w_i)_{i\in [n]}$ be defined by

1.6 $$\begin{aligned} w_i = [1-F]^{-1}(i/n), \end{aligned}$$

where *F* is a distribution function on $[0,\infty )$ for which we assume that there exists a $\tau \in (3,4)$ and $0<c_{\scriptscriptstyle F}<\infty$ such that

1.7 $$\begin{aligned} \lim _{x\rightarrow \infty }x^{\tau -1}[1-F(x)]= c_{\scriptscriptstyle F}, \end{aligned}$$

and where $[1-F]^{-1}$ is the generalized inverse function of $1-F$ defined, for $u\in (0,1)$, by

1.8 $$\begin{aligned}{}[1-F]^{-1}(u)=\inf \{ s:[1-F](s)\le u\}. \end{aligned}$$

By convention, we set $[1-F]^{-1}(1)=0$. Note that our inhomogeneity is chosen in such a way that the vertex weights $i\mapsto w_i$ are decreasing, with $w_1$ being the largest vertex weight.

For the setting in (1.3) and (1.6), by [11, Theorem 3.13], the number of vertices with degree *k*, which we denote by $N_k$, satisfies

1.9 $$\begin{aligned} N_k/n{\mathop {\longrightarrow }\limits ^{\scriptscriptstyle {\mathbb {P}}}}\mathbb {E}\Big [\mathrm {e}^{-W} \frac{W^k}{k!}\Big ], \qquad k\ge 0, \end{aligned}$$

where ${\mathop {\longrightarrow }\limits ^{\scriptscriptstyle {\mathbb {P}}}}$ denotes convergence in probability, and where *W* has distribution function *F* appearing in (1.6). We recognize the limiting distribution as a so-called *mixed Poisson distribution with mixing distribution F*, i.e., conditionally on $W=w$, the distribution is Poisson with mean *w*. As discussed in more detail in [22], since a Poisson random variable with large parameter *w* is closely concentrated around its mean *w*, the tail behavior of the degrees in our random graph is close to that of the distribution *F*. As a result, when (1.7) holds, and with $D_n$ the degree of a uniformly chosen vertex in [*n*], $\limsup _{n\rightarrow \infty } \mathbb {E}[D^a_n]<\infty$ when $a<\tau -1$ and $\limsup _{n\rightarrow \infty } \mathbb {E}[D^a_n]=\infty$ when $a\ge \tau -1$. In particular, the degree of a uniformly chosen vertex in [*n*] has finite second, but infinite third moment when (1.7) holds with $\tau \in (3,4)$.

Under the key assumption in (1.7),

1.10 $$\begin{aligned}{}[1-F]^{-1}(u)=\big (c_{\scriptscriptstyle F}/u\big )^{1/(\tau -1)}(1+o(1)), \quad u\downarrow 0, \end{aligned}$$

and the third moment of the degrees tends to infinity, i.e., with $W\sim F$, we have $\mathbb {E}[W^3]=\infty$. Define

1.11 $$\begin{aligned} \nu = \mathbb {E}[W^2]/\mathbb {E}[W], \end{aligned}$$

so that, again by (1.7), $\nu <\infty$. Then, by [11, Theorem 3.1] (see also [11, Sect. 16.4] for a detailed discussion on rank-1 inhomogeneous random graphs, of which our random graph is an example), when $\nu > 1$, there is one giant component of size proportional to *n*, while all other components are of smaller size *o*(*n*), and when $\nu \le 1$, the largest connected component contains a proportion of vertices that converges to zero in probability. Thus, the critical value of the model is $\nu =1$. The main goal of this paper is to investigate what happens close to the critical point, i.e., when $\nu =1$.

With the definition of the weights in (1.6) and for *F* such that $\nu =1$, we write $\mathcal {G}_n^0(\varvec{w})$ for the graph constructed with the probabilities in (1.3), while, for any fixed $\lambda \in {\mathbb {R}}$, we write $\mathcal {G}_n^\lambda (\varvec{w})$ when we use the weight sequence

1.12 $$\begin{aligned} \varvec{w}(\lambda )=(1+\lambda n^{-(\tau -3)/(\tau -1)})\varvec{w}. \end{aligned}$$

We shall assume that *n* is so large that $1+\lambda n^{-(\tau -3)/(\tau -1)}\ge 0$, so that ${w_i(\lambda )\ge 0}$ for all $i\in [n]$. When $\tau >4$, so that $\mathbb {E}[W^3]<\infty$, it was shown in [6, 22, 34] that the scaling limit of the random graphs studied here are (apart from a trivial rescaling of time and $\lambda$) *equal* to the scaling limit of the ordered connected components in the Erdős–Rényi random graph in Theorem 1.1. The rescaling of time and $\lambda$ is due to the variance of the step distribution of the cluster exploration process being unequal to 1 (see Sect. 1.2 below for more details on what we mean with ‘cluster exploration’). For the Erdős–Rényi random graph the step distribution has a Poisson distribution with parameter 1 minus one. When $\tau \in (3,4)$ the situation is entirely different, as discussed next.

Throughout this paper, we make use of the following standard notation. We let ${\mathop {\longrightarrow }\limits ^{d}}$ denote convergence in distribution, and ${\mathop {\longrightarrow }\limits ^{\scriptscriptstyle {\mathbb {P}}}}$ convergence in probability. For a sequence of random variables $(X_n)_{n\ge 1}$, we write $X_n=o_{\scriptscriptstyle \mathbb {P}}(b_n)$ when $|X_n|/b_n{\mathop {\longrightarrow }\limits ^{\scriptscriptstyle {\mathbb {P}}}}0$ as $n\rightarrow \infty$. For a non-negative function $n\mapsto g(n)$, we write $f(n)=O(g(n))$ when |*f*(*n*)| / *g*(*n*) is uniformly bounded, and $f(n)=o(g(n))$ when $\lim _{n\rightarrow \infty } f(n)/g(n)=0$. Furthermore, we write $f(n)=\Theta (g(n))$ if $f(n)=O(g(n))$ and $g(n)=O(f(n))$. Finally, we abbreviate

1.13 $$\begin{aligned} \alpha =1/(\tau -1),\qquad \rho =(\tau -2)/(\tau -1), \qquad \eta =(\tau -3)/(\tau -1). \end{aligned}$$

### The Scaling Limit for $\tau \in (3,4)$

We next recall two key results that we recently established in [7]:

#### Theorem 1.2

(Weak convergence of the ordered critical clusters for $\tau \in (3,4)$ [7]) Fix the Norros–Reittu random graph with weights $\varvec{w}(\lambda )$ defined in (1.6) and (1.12). Assume that $\nu =1$ and that (1.7) holds. Then, for all $\lambda \in {\mathbb {R}}$,

1.14 $$\begin{aligned} \big (|{\mathcal {C}}_{\scriptscriptstyle (1)}|n^{-\rho },|{\mathcal {C}}_{\scriptscriptstyle (2)}|n^{-\rho },\ldots \big ) {\mathop {\longrightarrow }\limits ^{d}}(\gamma _1(\lambda ),\gamma _2(\lambda ),\ldots ), \end{aligned}$$

in the product topology, for some non-degenerate limit $(\gamma _i(\lambda ))_{i\ge 1}$.

In order to further specify the scaling limit $(\gamma _i(\lambda ))_{i\ge 1}$, we need to introduce a continuous-time process $(\mathcal {S}_t)_{t\ge 0}$, referred to as a *thinned Lévy process*, and defined as

1.15 $$\begin{aligned} \mathcal {S}_t=b -abt+ct+\sum _{i=2}^{\infty } \frac{b}{i^{\alpha }} \Big [\mathcal {I}_i(t)-\frac{at}{i^{\alpha }}\Big ], \end{aligned}$$

where *a*, *b*, *c* have been identified in [7, Theorem 2.4] as $a=c_{\scriptscriptstyle F}^{\alpha }/\mathbb {E}[W]$, $b=c_{\scriptscriptstyle F}^{\alpha }$ and $c=\theta =\lambda + \zeta$ with

1.16 $$\begin{aligned} \zeta =\frac{c_{\scriptscriptstyle F}^{2\alpha }}{\mathbb {E}[W]} \sum _{i\ge 1} \left[ \int _{i-1}^i u^{-2\alpha }du-i^{-2\alpha }\right] \in (-\infty ,0) \end{aligned}$$

the constant given in [7, (2.18)]^1^. The process $(\mathcal {S}_t)_{t\ge 0}$ starts out positive. It can be positive or negative, and we will be interested in the first hitting time of $(\mathcal {S}_t)_{t\ge 0}$ of zero.

Further, here we use the notation

1.17 $$\begin{aligned} \mathcal {I}_i(t)=\mathbb {1}_{\{T_i\le t\}}, \end{aligned}$$

where $(T_i)_{i\ge 2}$ are independent exponential random variables with mean

1.18 $$\begin{aligned} \mathbb {E}[T_i]=i^{\alpha }/a. \end{aligned}$$

The term thinned Lévy process refers to the fact that $\mathcal {I}_i(t)$ can be interpreted as $\mathbb {1}_{\{N_i(t)\ge 1\}}$, where $(N_i(t))_{i\ge 1}$ are independent Poisson processes with rate $a/i^{\alpha }$. If we replace $\mathbb {1}_{\{N_i(t)\ge 1\}}$ by $N_i(t)$ in this representation, then the corresponding process is a Lévy process. In $(\mathcal {S}_t)_{t\ge 0}$, only the *first* point in these Poisson processes is counted, thus we can think about the Poisson processes as being *thinned*. See below for more details on the interpretation of $(\mathcal {S}_t)_{t\ge 0}$.

Let $H_1(0)$ denote the first hitting time of 0 of the process $(\mathcal {S}_t)_{t\ge 0}$, i.e.,

1.19 $$\begin{aligned} H_1(0)=\inf \{t\ge 0:\mathcal {S}_t=0\}, \end{aligned}$$

and ${\mathcal {C}}(1)$ the connected component to which vertex 1 (with the largest weight) belongs. We recall from [7, Theorem 2.1 and Proposition 3.7] that also $|{\mathcal {C}}(1)|n^{-\rho }$ converges in distribution:

#### Theorem 1.3

(Weak convergence of the cluster of vertex 1 for $\tau \in (3,4)$). Fix the Norros–Reittu random graph with weights $\varvec{w}(\lambda )$ defined in (1.6) and (1.12). Assume that $\nu =1$ and that (1.7) holds. Then, for all $\lambda \in {\mathbb {R}}$,

1.20 $$\begin{aligned} n^{-\rho }|{\mathcal {C}}(1)|{\mathop {\longrightarrow }\limits ^{d}}H_1^{a}(0), \end{aligned}$$

with $H_1^{a}(0)$ the hitting time of 0 of $(\mathcal {S}_t)_{t\ge 0}$ with $a=c_{\scriptscriptstyle F}^{\alpha }/\mathbb {E}[W]$, $b=c_{\scriptscriptstyle F}^{\alpha }$, $c=\theta$.

Let us informally describe how the process $(\mathcal {S}_t)_{t\ge 0}$ arises through a cluster exploration, and how it is linked to $H_1^{a}(0)$ in (1.20) as well as $(\gamma _i(\lambda ))_{i\ge 1}$ in (1.14). In Theorem 1.3, we explore the connected component of vertex 1 one vertex at a time in a breadth-first way, and keep track of the number of active vertices, which are vertices that are found to be in ${\mathcal {C}}(1)$, but whose neighbors have not yet been inspected whether they are in ${\mathcal {C}}(1)$. Let $\mathcal {S}_k^{\scriptscriptstyle (n)}$ be the number of active vertices after *k* steps, so that $\mathcal {S}_0^{\scriptscriptstyle (n)}=1.$ Obviously, $|{\mathcal {C}}(1)|=\inf \{k :\mathcal {S}_k^{\scriptscriptstyle (n)}=0\}$, since we are done with the exploration of a cluster when there are no unexplored vertices left, and we explore one vertex at a time. By construction, $\mathcal {S}_1^{\scriptscriptstyle (n)}$ is the number of neighbors of vertex 1, which can be seen to be close to $w_1\approx b n^{\alpha }$. Thus, the exploration process can be expected to be of order $n^{\alpha }$, and we will rescale $\mathcal {S}_1^{\scriptscriptstyle (n)}$ by a factor $n^{-\alpha }$.

As explained in more detail in [7] and for the edge-probabilities in (1.3), the exploration can be performed rather effectively in terms of a marked branching process with mixed Poisson offspring distribution. Here, an unexplored vertex in the branching process, *v*, first draws a mark $M_v$ for which $\mathbb {P}(M_v=i)=w_i/\ell _n$, and after this, it draws a Poisson number of children with mean $w_{M_v}$. The connection to the cluster exploration in the graph is obtained by *thinning* all vertices whose mark has appeared earlier. Here, we can think of the mark $M_v=i$ as indicating that the vertex *v* in the branching process is being mapped to vertex *i* in the graph.

The largest weights correspond to the small values of $i\in [n]$. The amount of time it takes us to draw a mark corresponding to vertex *i* is of the order $\ell _n/w_i$, which is of order $n^{\rho } a/i^\alpha$, which suggests that $(n^{-\alpha }\mathcal {S}_{tn^\rho }^{\scriptscriptstyle (n)})_{t\ge 0}$ converges in distribution to some process $(\mathcal {S}_t)_{t\ge 0}$. Further, the first time that $i\ge 2$ is chosen, $T_i^{\scriptscriptstyle (n)}$, satisfies $n^{-\rho } T_i^{\scriptscriptstyle (n)}{\mathop {\longrightarrow }\limits ^{d}}T_i$, where $T_i$ is exponential with rate $a/i^\alpha$. Further, vertex *i* has roughly $w_i\approx n^{\alpha } b/i^\alpha$ neighbors, so that $\mathcal {S}_k^{\scriptscriptstyle (n)}$ makes a jump of order $n^{\alpha } b/i^\alpha$ when *i* is found to be in ${\mathcal {C}}(1)$. This informally explains the process (1.15)–(1.18), while (1.19) explains that when the exploration process hits zero, then the cluster is fully explored. Turning this into a formal proof was one of the main steps in [7].

The above description does not yet describe the scaling limit $(\gamma _i(\lambda ))_{i\ge 1}$ in (1.14). For this, we note that after the exploration of ${\mathcal {C}}(1)$, we need to explore the clusters of the (high-weight) vertices that are not part of ${\mathcal {C}}(1)$. We do this by taking the vertex with largest weight that is not in ${\mathcal {C}}(1)$, which in the scaling limit corresponds to the smallest *i* for which $\mathcal {I}_i(H_1(0))=0$, and start exploring the cluster of that vertex. This is again done by using processes similar to $(\mathcal {S}_t)_{t\ge 0}$, but changes arise due to the *depletion-of-points* effect. Indeed, since ${\mathcal {C}}(1)$ is fully explored, in later explorations those vertices cannot arise again. We refrain from describing this in more detail, as it is not needed in this paper. We repeat this procedure, and explore the connected components of unexplored vertices of the highest weights one by one. After performing these explorations infinitely often, we obtain $(\gamma _i(\lambda ))_{i\ge 1}$ as the ordered vector of hitting times of zero of these cluster exploration processes. Some more details are given in Sect. 2.4.

By scaling, $H_1^{a}(0)/a$ for some *a*, *b*, *c* has the same distribution as the hitting time $H_1(0)$ obtained by taking $b'=a'=1$, and $c'=c/(ab)=(\lambda +\zeta )/(ab)$. We shall reparametrize $a'=b'=1$ and let

1.21 $$\begin{aligned} \mathcal {S}_t=1+\tilde{\beta }t+\sum _{i=2}^{\infty } c_i [\mathcal {I}_i(t)-c_i t], \end{aligned}$$

where we set

1.22 $$\begin{aligned} \tilde{\beta }=\beta -1\qquad \text{ with } \qquad \beta =c'=\theta /(ab)=(\lambda + \zeta )/(ab), \end{aligned}$$

have used the notation

1.23 $$\begin{aligned} c_i = i^{-\alpha }, \end{aligned}$$

and where $\mathcal {I}_i(t)$ is defined in (1.17)–(1.18) with *a* replaced by $a'=1$. This scaling is convenient, as it reduces the clutter in our notation.

### Main Results

In this section we state our main results. Recall $\gamma _1(\lambda )$ from (1.14). Our Main Theorem establishes a generalization of Pittel’s result in (1.2) to our rank-1 inhomogeneous random graph with power-law exponents $\tau \in (3,4)$:

#### Main Theorem

(Tail behavior scaling limit for $\tau \in (3,4)$). When $u\rightarrow \infty$, there exists $I>0$ independent of $\lambda$ and $A=A(\lambda ), \kappa _{i,j}=\kappa _{i,j}(\lambda )$ such that

1.24 $$\begin{aligned} \mathbb {P}(\gamma _1(\lambda )>a u)= \frac{A}{u^{(\tau -1)/2}}{\mathrm {e}}^{-Iu^{\tau -1} +u^{\tau -1}\sum _{i+j \ge 1} \kappa _{ij}u^{-i(\tau -2)-j(\tau -3)}}(1+o(1)). \end{aligned}$$

The constants *I*, *A* and $\kappa _{ij}$ are specified in Sect. 2. By scaling, these constants only depend on *a*, *b* through $c'=c/(ab)=(\lambda +\zeta )/(ab)$, any other dependence disappears since the law of $H_1(0)$ only depends on $c'$. Since $\tau \in (3,4)$, the sum over *i*, *j* such that $i+j\ge 1$ is in fact *finite*, as we can ignore all terms for which $\tau -1-i(\tau -2)-j(\tau -3)<0$. We also see that the Main Theorem connects up nicely with Pittel’s result in (1.2) that arises for $\tau =4$, as for example seen in the fact that the exponent of *u* in the exponential is equal to 3 for $\tau =4$ and the exponent in the power of *u* in the prefactor is equal to 3 / 2, as in (1.2). That these powers depend sensitively on $\tau$ is a manifestation of the importance of the *inhomogeneity*, which we will see throughout this paper.

Aside from the Main Theorem, we prove two further theorems about the structure of the largest connected component when it is large. The first theorem concerns the probability that $H_1^a(0)>u$ for some $u>0$ large, where $H_1^a(0)$ is the weak limit of $n^{-\rho } |{\mathcal {C}}(1)|$ identified in Theorem 1.3. This is achieved by investigating the hitting time $H_1(0)$ of 0 of the process $(\mathcal {S}_t)_{t\ge 0}$ in (1.21).

#### Theorem 1.4

(Tail behavior scaling limit cluster vertex 1 for $\tau \in (3,4)$). When $u\rightarrow \infty$, there exists $I>0$ independent of $\tilde{\beta }$ and $A=A(\tilde{\beta })$ and $\kappa _{ij}(\tilde{\beta })\in {\mathbb R}$ such that

1.25 $$\begin{aligned}&\mathbb {P}(H_1(0)>u)= \mathbb {P}(H_1^a(0)>au) \nonumber \\&\quad =\frac{A}{u^{(\tau -1)/2}} {\mathrm {e}}^{-Iu^{\tau -1}+u^{\tau -1}\sum _{i+j \ge 1} \kappa _{ij}u^{-i(\tau -2)-j(\tau -3)}}(1+o(1)). \end{aligned}$$

The constants *I*, *A* and $\kappa _{ij}$ are equal to those in the Main Theorem. Comparing the Main Theorem and Theorem 1.4, we see that $\mathbb {P}(H_1(0)>u)=\mathbb {P}(\gamma _1(\lambda )>a u) \cdot (1+o(1))$.

This has the interpretation that vertex 1, which is the vertex with the largest weight in our rank-1 inhomogeneous random graph, is with overwhelming probability in the largest connected component when this largest connected component is quite large.

**Fig. 1:**
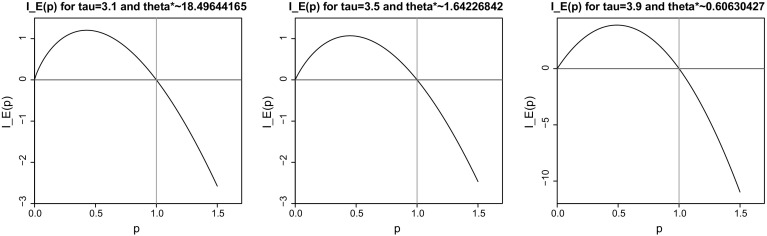
Numerical plots of $p \mapsto I_E(p)$ for $\tau =3.1, 3.5$ and 3.9. In a first step, $\theta ^*$ was determined as the unique $\theta$ such that $I_E(1)=I_E(1,\theta )=0$

We can even go one step further and study the optimal trajectory the process $t\mapsto \mathcal {S}_t$ takes in order to achieve the unlikely event that $H_1(0)>u$ when *u* is large. In order to describe this trajectory, we need to introduce some further notation. In the proof, it will be crucial to *tilt* the distribution, i.e., to investigate the measure $\tilde{\mathbb {P}}$ with Radon–Nikodym derivative ${\mathrm {e}}^{\theta u \mathcal {S}_{u}}/\mathbb {E}[{\mathrm {e}}^{\theta u \mathcal {S}_{u}}]$, for some appropriately chosen $\theta$. The selection of an appropriate $\theta$ for the thinned Lévy process$(\mathcal {S}_t)_{t\ge 0}$ is quite subtle, and has been the main topic of our paper [1]. The main results from paper [1] are reported in Sect. 2, and will play an important role in the present analysis. We refer to below (2.13) for the definition of $\theta ^*$ that appears in the description of the optimal trajectory that is identified in the following theorem (Fig. 1):

#### Theorem 1.5

(Optimal trajectory). For $p\in [0,1]$, define

1.26 $$\begin{aligned} I_{\scriptscriptstyle E}(p)=(\tau -1)\int _0^{\infty } \Big ( \frac{ {\mathrm {e}}^{\theta ^* v} ( 1 - {\mathrm {e}}^{-p v} ) }{ {\mathrm {e}}^{\theta ^* v}(1- {\mathrm {e}}^{-v}) + {\mathrm {e}}^{-v} } - p v\Big ) \frac{ dv }{ v^{\tau - 1} }, \end{aligned}$$

with $\theta ^*$ as defined below (2.13). Then, for $u\rightarrow \infty$ and for any $\varepsilon >0$,

1.27 $$\begin{aligned} \mathbb {P}\Big (\sup _{p\in [0,1]} |\mathcal {S}_{pu}-u^{\tau -2}I_{\scriptscriptstyle E}(p)|\le u^{\tau -2}\varepsilon \mid H_1(0)>u\Big ) =1-o(1). \end{aligned}$$

Our Main Theorem follows by combining Theorems 1.4 and 1.5, and showing that, for *u* large, the probability that $1\in {\mathcal {C}}_{\scriptscriptstyle (1)}$ is overwhelmingly large. This argument is performed in detail in Sect. 2.4:

**Brownian motion on a parabola** Note that substituting $\tau =4$ into (1.24) yields $\frac{A}{u^{3/2}}{\mathrm {e}}^{-Iu^{3}+\kappa _{01} u^2+(\kappa _{10}+\kappa _{02})u}(1+o(1))$, which agrees with the result of Pittel in (1.2). This suggests a smooth transition from the case $\tau \in (3,4)$ to the case $\tau >4$. We next further explore this relation.

Consider the process $(W^\lambda _t)_{t\ge 0}=(W_t + \lambda t -t^2/2)_{t\ge 0}$ with $(W_t)_{t\ge 0}$ a standard Wiener process as mentioned in Theorem 1.1. We now apply the technique of exponential change of measure to this process. First note that the moment generating function of $W_u^{\lambda }$ can be computed as

1.28 $$\begin{aligned} \log \phi (u; \vartheta ) \equiv \log \mathbb {E}\left[ {\mathrm {e}}^{\vartheta u W_u^\lambda }\right] =\vartheta u\left( \lambda u-\tfrac{1}{2} u^2+\tfrac{1}{2} \vartheta u^2\right) \end{aligned}$$

and let $\theta ^{*}_u$ be the solution of $\theta ^{*}_u=\arg \min _{\vartheta } \log \phi (u; \vartheta )$, which is given by

1.29 $$\begin{aligned} \theta ^{*}_u=\frac{1}{2}-\frac{\lambda }{u}. \end{aligned}$$

The main term is

1.30 $$\begin{aligned} \phi (u)=\phi (u; \theta ^{*}_u)=\mathbb {E}\left[ {\mathrm {e}}^{\theta ^{*}_u u W_u^\lambda }\right] ={\mathrm {e}}^{-\frac{1}{8} u^3+\frac{1}{2} \lambda u^2-\frac{1}{2} \lambda ^2 u}={\mathrm {e}}^{-\frac{1}{8}u(u-2\lambda )^2}. \end{aligned}$$

Noting that

1.31 $$\begin{aligned} \mathbb {P}(\gamma _1(\lambda )>u)\le \mathbb {P}\left( W_u^{\lambda }>0\right) \le \mathbb {P}\left( {\mathrm {e}}^{\theta ^{*}_u u W_u^{\lambda }}>1\right) \le \mathbb {E}\left[ {\mathrm {e}}^{\theta ^{*}_u u W_u^\lambda }\right] , \end{aligned}$$

we see that this upper bound agrees to leading order with the result of Pittel in (1.2). In order to derive the full asymtptotics in (1.2), one can define the measure

1.32 $$\begin{aligned} \widetilde{\mathbb {P}}(E)=\phi (u)^{-1}\mathbb {E}\left[ {\mathrm {e}}^{\theta ^{*}_u u W_u^\lambda } \mathbb {1}_{E}\right] , \end{aligned}$$

rewrite

1.33 $$\begin{aligned} \mathbb {P}(\gamma _1(\lambda )>u) = \phi (u) \widetilde{\mathbb {E}}\left[ {\mathrm {e}}^{\theta ^{*}_u u W_u^\lambda } \mathbb {1}_{\{\gamma _1(\lambda )>u\}}\right] , \end{aligned}$$

and then deduce the asymptotics of the latter expectation in full detail. Our analysis will be based on this intuition, now applied to a more involved, so-called thinned Lévy, stochastic process.

## Overview of the Proofs

In this section, we give the overview of the proofs of Theorems 1.4–1.3. The point of departure for our proofs is the conjecture that $\mathbb {P}(H_1(0)>u)\approx \mathbb {P}(\mathcal {S}_u>0)$ for large *u*. The event $\{H_1(0)>u\}$ obviously implies $\{\mathcal {S}_u>0\}$, but because of the strong downward drift of the process $(\mathcal {S}_t)_{t\ge 0}$, it seems plausible that both events are roughly equivalent.

In [1] a detailed study was presented on the large deviations behavior of the process $(\mathcal {S}_t)_{t\ge 0}$. Using exponential tilting of measure the following two theorems were proved.

### Theorem 2.1

(Exact asymptotics tail $\mathcal {S}_u$ [1, Theorem 1.1]). There exists $I, D>0$ and $\kappa _{ij}\in {\mathbb R}$ such that, as $u\rightarrow \infty$,

2.1 $$\begin{aligned} \mathbb {P}(\mathcal {S}_u>0)= \frac{D}{u^{(\tau -1)/2}} {\mathrm {e}}^{-Iu^{\tau -1}+u^{\tau -1}\sum _{i+j \ge 1} \kappa _{ij}u^{-i(\tau -2)-j(\tau -3)}}(1+o(1)). \end{aligned}$$

### Theorem 2.2

(Sample path large deviations [1, Theorem 1.2]). There exists a function $p\mapsto I_{\scriptscriptstyle E}(p)$ on [0, 1] such that, for any $\varepsilon >0$ and $p\in [0,1)$,

2.2 $$\begin{aligned} \lim _{u\rightarrow \infty } \mathbb {P}\big (\big |\mathcal {S}_{pu}-u^{\tau -2}I_{\scriptscriptstyle E}(p)|\le \varepsilon u^{\tau -2}\mid \mathcal {S}_u>0)=1. \end{aligned}$$

In [1] it is explained that specific challenges arise in the identification of a tilted measure due to the power-law nature of $(\mathcal {S}_t)_{t\ge 0}$. General principles prescribe that the tilt should follow from a variational problem, but in the case of $(\mathcal {S}_t)_{t\ge 0}$ this involves a Riemann sum that is hard to control. In [1] this Riemann sum is approximated by its limiting integral, and it is proved that the tilt that follows from the corresponding approximate variational problem is sufficient to establish the large deviations results in Theorems 2.1 and 2.2. Details about this tilted measure are presented in Sect. 2.1.

It is clear that Theorems 2.1 and 2.2 for the event $\{\mathcal {S}_u>0\}$ are the counterparts of Theorems 1.4 and 1.5 for $\{H_1(0)>u\}$. Let us now sketch how we make the conjecture that $\mathbb {P}(H_1(0)>u)\approx \mathbb {P}(\mathcal {S}_u>0)$ for large *u* formal. We show that $\mathbb {P}(H_1(0)>u)$ has the same asymptotic behavior as $\mathbb {P}(\mathcal {S}_u>0)$ in (2.1), with the same constants except for the constant *D*. Despite the similarity of this result, the proof method we shall use is entirely different from the exponential tilting in [1]. In order to establish the asymptotics for $\mathbb {P}(H_1(0)>u)$, we establish sample path large deviations, not conditioned on the event $\{\mathcal {S}_u>0\}$, but on the event $\{H_1(0)>u\}$. This is much harder, since we have to investigate the probability that $\mathcal {S}_t>0$ for *all* $t\in [0,u]$. However, this is also more important, as only the hitting times $H_1(0)$ give us asymptotics of the limiting cluster sizes. In order to prove these strong sample-path properties, we first prove that, under the tilted measure, $\mathcal {S}_t$ is close to its expected value for a finite, but large, number of *t*’s, followed by a proof that the path cannot deviate much in the small time intervals between these times.

Now here is our **strategy for the proofs.** We extend the conjecture $\mathbb {P}(H_1(0)>u)\approx \mathbb {P}(\mathcal {S}_u>0)$ by a conjectured sample path behavior that says that, under the tilted measure, the typical sample path of $(\mathcal {S}_t)_{t\ge 0}$ that leads to the event $\{\mathcal {S}_u>0\}$ remains positive and hence implies $\{H_1(0)>u\}$. To be more specific, we divide up this likely sample path into three parts: the early part, the middle part, and the end part. Our proof consists of treating each of these parts separately. We shall prove consecutively that with high probability the process:

(i) Does not cross zero in the initial part of the trajectory (‘no early hits’);
(ii) Is high up in the state space in the middle part of the trajectory, while experiencing small fluctuations, and therefore does not hit zero (‘no middle ground’);
(iii) Is forced to remain positive until the very end.

In the last step, we have to be *very* careful, and it is in this step that it will turn out that the constant *D* arising in the asymptotics of $\mathbb {P}(\mathcal {S}_u>0)$ in (2.1) is *different* from the constant *A* arising in the asymptotics of $\mathbb {P}(H_1(0)>u)$ in (1.25). This is due to the fact that even when $\mathcal {S}_u>0$, the path could dip below zero right before time *u* and does so with non-vanishing probability. The proof reveals that then it will do so in the time interval $[u-Tu^{-(\tau -2)},u]$ for some large *T*.

We next summarize the technique of exponential tilting developed in [1] for the thinned Lévy process $(\mathcal {S}_t)_{t\ge 0}$ with $\tau \in (3,4)$, which allows us to give more details about how we shall establish the conjectured sample path behavior for each of the three parts described above.

### Tilting and Properties of the Tilted Process

All results presented in this subsection are proved in [1].

**Exponential tilting** Parts of this section are taken almost verbatim from [1]. We use the notion of exponential tilting of measure in order to rewrite

2.3 $$\begin{aligned} \mathbb {P}(\mathcal {S}_{u}>0) =\phi (u;\vartheta ) \widetilde{\mathbb {E}}_{\vartheta }\left[ {\mathrm {e}}^{-\vartheta u \mathcal {S}_u}\mathbb {1}_{\{\mathcal {S}_{u}>0\}}\right] , \end{aligned}$$

where $\vartheta$ is chosen later on. For every event *E*, define the measure $\widetilde{\mathbb {P}}_{\vartheta }$ with corresponding expectation $\widetilde{\mathbb {E}}_{\vartheta }$ by means of the equality

2.4 $$\begin{aligned} \widetilde{\mathbb {P}}_{\vartheta }(E)=\frac{1}{\phi (u;\vartheta )}\mathbb {E}_{\vartheta }\left[ {\mathrm {e}}^{\vartheta u \mathcal {S}_u}\mathbb {1}_{E}\right] , \end{aligned}$$

with normalizing constant $\phi (u;\vartheta )$ given by

2.5 $$\begin{aligned} \phi (u; \vartheta )=\mathbb {E}\left[ {\mathrm {e}}^{\vartheta u \mathcal {S}_u}\right] . \end{aligned}$$

In terms of this notation, we are interested in

2.6 $$\begin{aligned} \mathbb {P}(H_1(0)>u) =\phi (u;\vartheta ) \widetilde{\mathbb {E}}_{\vartheta }\left[ {\mathrm {e}}^{-\vartheta u \mathcal {S}_u}\mathbb {1}_{\{\mathcal {S}_{[0,u]}>0\}}\right] , \end{aligned}$$

where we write $\{\mathcal {S}_{[0,u]}>0\}=\{\mathcal {S}_{t}>0\forall t\in [0,u]\}$.

We now explain in more detail how to choose a good $\vartheta$. The independence of the indicators $(\mathcal {I}_i(u))_{i\ge 2}$ yields

2.7 $$\begin{aligned} \phi (u; \vartheta )&=\mathbb {E}[{\mathrm {e}}^{\vartheta u \mathcal {S}_u}]={\mathrm {e}}^{\vartheta u(1+\tilde{\beta }u)}\prod _{i=2}^{\infty }{\mathrm {e}}^{-\vartheta u^2 c_i^2} \Big ({\mathrm {e}}^{- c_iu} +{\mathrm {e}}^{\vartheta c_iu} (1-{\mathrm {e}}^{-c_iu})\Big ) \nonumber \\&={\mathrm {e}}^{\vartheta u(1+\tilde{\beta }u)} {\mathrm {e}}^{\sum _{i=2}^{\infty }f(i/u^{\tau -1};\vartheta )} \end{aligned}$$

with

2.8 $$\begin{aligned} f(x;\vartheta )=\log \big (1+{\mathrm {e}}^{-x^{-\alpha }} ({\mathrm {e}}^{-\vartheta x^{-\alpha }}-1)\big )+\vartheta x^{-\alpha }-\vartheta x^{-2\alpha }. \end{aligned}$$

The function $x\mapsto f(x;\vartheta )$ is integrable at $x=0$ and at $x=\infty$, so the above sum can be approximated by the integral

2.9 $$\begin{aligned} \sum _{i=2}^{\infty }f(i/u^{\tau -1};\vartheta )=u^{\tau -1} \int _0^{\infty }f(x;\vartheta ) dx+e_\vartheta (u) \equiv u^{\tau -1}\Lambda (\vartheta )+ e_\vartheta (u), \end{aligned}$$

for some error term $u\mapsto e_\vartheta (u)$ given by

2.10 $$\begin{aligned} e_\vartheta (u)=\vartheta \left\{ u [\zeta (\alpha )-1] - u^2[\zeta (2\alpha )-1] \right\} + o_\vartheta (1), \end{aligned}$$

where $\alpha =1/(\tau -1)$ and the Riemann zeta functions $\zeta (\cdot )$ defined as

2.11 $$\begin{aligned} \zeta (s)=\lim _{N\rightarrow \infty }\left\{ \sum _{n=1}^N n^{-s}-\frac{N^{1-s}}{1-s}- \frac{1}{2} N^{-s}\right\} , \quad {\mathrm {Re}}(s)>-1, s\ne 1, \end{aligned}$$

where ${\mathrm {Re}}(s)$ denotes the real part of $s\in {\mathbb C}$. Equation (2.11) follows from Euler–Maclaurin summation [21, p. 333]. The error term in (2.10) converges to 0 uniformly for $\vartheta$ in compact sets bounded away from zero. As a result,

2.12 $$\begin{aligned} \phi (u; \vartheta ) ={\mathrm {e}}^{u^{\tau -1}\Lambda (\vartheta )+\vartheta u(\zeta (\alpha )+(\tilde{\beta }-\zeta (2\alpha )+1) u)+o_\vartheta (1)}. \end{aligned}$$

Let $\theta ^{*}_u$ be the solution of

2.13 $$\begin{aligned} \theta ^{*}_u=\arg \min _{\vartheta } \Big [\Lambda (\vartheta ) +\vartheta u^{2-\tau }(\zeta (\alpha )+(\tilde{\beta }-\zeta (2\alpha )+1) u)\Big ]. \end{aligned}$$

Moreover, let $\theta ^{*}$ be the value of $\vartheta$ where $\vartheta \mapsto \Lambda (\vartheta )$ is minimal. It follows easily that $I\equiv -\Lambda (\theta ^{*})>0$ and that $\theta ^{*}$ is unique. In [1, Lemma 3.6], we have seen that $\theta ^{*}_u=\theta ^{*}+o(1)$. Further, $\theta ^{*}>0$ by [1, Lemma 3.5]. Set $\phi (u)=\phi (u;\theta ^{*}_u)$. The asymptotics of $\phi (u)$ are as follows.

#### Proposition 2.3

(Asymptotics of main term [1, Proposition 2.1]). As $u\rightarrow \infty$, and with $I=-\min _{\vartheta \ge 0} \Lambda (\vartheta )>0$, there exist $\kappa _{ij}\in {\mathbb R}$ such that

2.14 $$\begin{aligned} \phi (u)=\mathbb {E}\left[ {\mathrm {e}}^{\theta ^{*}_u u \mathcal {S}_u}\right] ={\mathrm {e}}^{-Iu^{\tau -1}+u^{\tau -1}\sum _{i+j \ge 1} \kappa _{ij}u^{-i(\tau -2)-j(\tau -3)}}(1+o(1)). \end{aligned}$$

**Properties of the process under the tilted measure** In what follows, take $\vartheta =\theta ^{*}_u$, and let $\widetilde{\mathbb {P}}=\widetilde{\mathbb {P}}_{\theta ^{*}_u}$ with corresponding expectation $\widetilde{\mathbb {E}}=\widetilde{\mathbb {E}}_{\theta ^{*}_u}$. Abbreviate $\theta =\theta ^{*}_u$. Under this new measure, the rare event of $\mathcal {S}_u$ being positive becomes quite likely. To describe these results, let us introduce some notation. Recall from (1.26) that, for $p\in [0,1]$,

2.15 $$\begin{aligned} I_{\scriptscriptstyle E}(p) =(\tau -1)\int _0^{\infty }\Big ( \frac{ {\mathrm {e}}^{\theta ^* v} ( 1 - {\mathrm {e}}^{-p v} ) }{ {\mathrm {e}}^{\theta ^* v}(1- {\mathrm {e}}^{-v}) + {\mathrm {e}}^{-v} } - p v\Big ) \frac{ dv }{ v^{\tau - 1} }, \end{aligned}$$

where we take $\vartheta =\theta ^{*}$, which turns out to be the limit of $\theta ^{*}_u$ as $u\rightarrow \infty$ (see, e.g., [1, Lemma 3.6]). The asymptotic mean of the process $p\mapsto \mathcal {S}_{pu}$ conditionally on $\mathcal {S}_u > 0$ can be described with the help of the function $p\mapsto I_{\scriptscriptstyle E}(p)$, cf. Theorem 2.2. One easily checks that

2.16 $$\begin{aligned} I_{\scriptscriptstyle E}(0)=0,\qquad \text { and } I_{\scriptscriptstyle E}(1)=0, \end{aligned}$$

the latter by definition of $\theta ^{*}$, as $0 = \Lambda '(\theta ^{*}) = I_{\scriptscriptstyle E}(1)$. Finally,

2.17 $$\begin{aligned} I_{\scriptscriptstyle E}(p)>0 \text{ for } \text{ every } p\in (0,1), \end{aligned}$$

and

2.18 $$\begin{aligned} I_{\scriptscriptstyle E}'(0)>0 \text{ and } I_{\scriptscriptstyle E}'(1)<0. \end{aligned}$$

#### Lemma 2.4

(Expectation of $\mathcal {S}_t$ [1, Lemma 2.2]). As $u\rightarrow \infty$,

- $$\widetilde{\mathbb {E}}[\mathcal {S}_{t}] = u^{\tau -2}I_{\scriptscriptstyle E}(t/u)+ O(1+t + t |\theta ^*- \theta _u^*| u^{\tau -3})$$ uniformly in $$t\in [0,u]$$.
- $$\widetilde{\mathbb {E}}[\mathcal {S}_{t}-\mathcal {S}_u] = u^{\tau -2}I_{\scriptscriptstyle E}(t/u)+ O(u-t + u^{-1} + |\theta ^*- \theta _u^*| u^{\tau -2})$$ uniformly in $$t\in [u/2,u]$$.
- $$\widetilde{\mathbb {E}}[\mathcal {S}_{t}-\mathcal {S}_u] = u^{\tau -3}I_{\scriptscriptstyle E}'(1)(t-u)(1+o(1))+ O(u^{-1})$$ when $$u-t=o(u)$$.
- $$u\widetilde{\mathbb {E}}[\mathcal {S}_u]=o(1)$$ when $$u\rightarrow \infty $$.

We will also need some consequences of the asymptotic properties of $\widetilde{\mathbb {E}}[\mathcal {S}_{t}]$. This is stated in the following corollary:

#### Corollary 2.5

As $$u\rightarrow \infty $$,

- $\widetilde{\mathbb {E}}[\mathcal {S}_{t}]\ge \underline{c} t u^{\tau -3}$ and $\widetilde{\mathbb {E}}[\mathcal {S}_{t}]\le \overline{c} t u^{\tau -3}$ uniformly for $t\in [\varepsilon ,u/2]$, where $0<\underline{c}<\overline{c}<\infty$;
- $\widetilde{\mathbb {E}}[\mathcal {S}_{u-t}-\mathcal {S}_u]\ge \underline{c} t u^{\tau -3}$ and $\widetilde{\mathbb {E}}[\mathcal {S}_{u-t}-\mathcal {S}_u]\le \overline{c} t u^{\tau -3}$ uniformly for $t\in [T u^{-(\tau -2)},u/2]$, where $0<\underline{c}<\overline{c}<\infty$;
- $\widetilde{\mathbb {E}}[\mathcal {S}_{t}]=\widetilde{\mathbb {E}}[\mathcal {S}_{t_1}](1+o(1))$ for $t\in [t_1,t_2]$ and $t_1\in [\varepsilon , u/2]$ and $t_2-t_1=O(u^{-(\tau -2)})$;
- $\widetilde{\mathbb {E}}[\mathcal {S}_{t}]=\widetilde{\mathbb {E}}[\mathcal {S}_{t_1}](1+o_{\scriptscriptstyle T}(1))$ for $t\in [t_1,t_{2}]$ and $t_1\in [u/2, u-Tu^{-(\tau -2)}]$, where $o_{\scriptscriptstyle T}(1)$ denotes a quantity *c*(*T*, *u*) such that $\lim _{T\rightarrow \infty } \limsup _{u\rightarrow \infty }c(T,u)=0$ and $t_2-t_1=O(u^{-(\tau -2)})$.

#### Proof

Part (a) for $t\in [\varepsilon ,\varepsilon u]$ for $\varepsilon >0$ sufficiently small follows from Lemma 2.4(a) together with the facts that $I_{\scriptscriptstyle E}(0)=0$, $I_{\scriptscriptstyle E}'(0)>0$, and that $1+t + t |\theta ^*- \theta _u^*| u^{\tau -3}=o(t u^{\tau -3})$. The fact that $I_{\scriptscriptstyle E}'(0)>0$ also implies that $\underline{c}$ can be taken to be strictly positive. For $t \in [\varepsilon u, u/2]$, Part (a) follows from the fact that $I_{\scriptscriptstyle E}(p)>0$ for all $p\in [\varepsilon ,1/2]$ and that $1+t + t |\theta ^*- \theta _u^*| u^{\tau -3}=o(u^{\tau -2})$.

Part (b) follows as Part (a), now using Lemma 2.4(b) together with the fact that $I_{\scriptscriptstyle E}(1)=0$, $I_{\scriptscriptstyle E}'(1)<0$.

Part (c) follows from Lemma 2.4(a), by subtracting the two terms. Note that the error term $O(1+t_1 + t_1 |\theta ^*- \theta _u^*| u^{\tau -3})$ is $o(t_1 u^{\tau -3})$ since $t_1\ge \varepsilon$, while $\widetilde{\mathbb {E}}[\mathcal {S}_{t_1}]=\Theta (t_1 u^{\tau -3})$ by Part (a) of this corollary. Further, note that

2.19 $$\begin{aligned} u^{\tau -2}[I_{\scriptscriptstyle E}(t/u)-I_{\scriptscriptstyle E}(t_1/u)]=O\left( u^{\tau -2} \max _{p\in [0,1]} |I_{\scriptscriptstyle E}'(p)| (t_2-t_1)\right) =O(1), \end{aligned}$$

which is $$o(1)\widetilde{\mathbb {E}}[\mathcal {S}_{t_1}]$$.

Part (d) follows again from Lemma 2.4(a) by subtracting the two terms. Note again that the error term $O(1+t_1 + t_1 |\theta ^*- \theta _u^*| u^{\tau -3})$ is $o(t_1 u^{\tau -3})$, while $\widetilde{\mathbb {E}}[\mathcal {S}_{t_1}]=\Theta (t_1 u^{\tau -3})$ by part (b) of this corollary and Lemma 2.4(a). Further, note that

2.20 $$\begin{aligned} u^{\tau -2}[I_{\scriptscriptstyle E}(t/u)-I_{\scriptscriptstyle E}(t_1/u)]=O\left( u^{\tau -2} \max _{p\in [0,1]} |I_{\scriptscriptstyle E}'(p)| (t_2-t_1)\right) =O_T(1), \end{aligned}$$

which is $$o_{\scriptscriptstyle T}(1)\widetilde{\mathbb {E}}[\mathcal {S}_{t_1}]$$. $$\square $$

The next lemma gives asymptotic properties of the variance of $\mathcal {S}_{t}$. Define, for $p\in [0,1]$,

2.21 $$\begin{aligned} I_{\scriptscriptstyle V}(p)= (\tau -1)\int _0^{\infty } \frac{ {\mathrm {e}}^{\theta ^{*}v}(1 - {\mathrm {e}}^{-p v} ) }{ {\mathrm {e}}^{\theta ^{*}v}(1- {\mathrm {e}}^{-v}) + {\mathrm {e}}^{-v} } \Big (1- \frac{ {\mathrm {e}}^{\theta ^{*}v} ( 1 - {\mathrm {e}}^{-p v} ) }{ {\mathrm {e}}^{\theta ^{*}v}(1- {\mathrm {e}}^{-v}) + {\mathrm {e}}^{-v} } \Big ) \frac{ dv }{ v^{\tau - 2} } \end{aligned}$$

and

2.22 $$\begin{aligned} J_{\scriptscriptstyle V}(p)&= (\tau -1)\int _0^\infty \frac{ {\mathrm {e}}^{\theta ^{*}v}( {\mathrm {e}}^{-p v} - {\mathrm {e}}^{-v}) }{ {\mathrm {e}}^{\theta ^{*}v}(1- {\mathrm {e}}^{-v}) + {\mathrm {e}}^{-v} } \Big (1- \frac{ {\mathrm {e}}^{\theta ^{*}v} ( {\mathrm {e}}^{-p v} - {\mathrm {e}}^{-v}) }{ {\mathrm {e}}^{\theta ^{*}v}(1- {\mathrm {e}}^{-v}) + {\mathrm {e}}^{-v} } \Big ) \frac{ dv }{ v^{\tau - 2} }, \end{aligned}$$

2.23 $$\begin{aligned} G_{\scriptscriptstyle V}(p)&= (\tau -1)\int _0^\infty \frac{ {\mathrm {e}}^{2\theta ^{*}v}( 1-{\mathrm {e}}^{-p v})({\mathrm {e}}^{-pv} - {\mathrm {e}}^{-v}) }{ \left( {\mathrm {e}}^{\theta ^{*}v}(1- {\mathrm {e}}^{-v}) + {\mathrm {e}}^{-v}\right) ^2 }{dv \over v^{\tau -2}}. \end{aligned}$$

Again, it is not hard to check that

2.24 $$\begin{aligned} 0<I_{\scriptscriptstyle V}(p)<\infty \text{ for } \text{ every } p\in (0,1], \text{ while } I_{\scriptscriptstyle V}(0)=0, \end{aligned}$$

and

2.25 $$\begin{aligned} 0<J_{\scriptscriptstyle V}(p)<\infty \text{ for } \text{ every } p\in [0,1), \text{ while } J_{\scriptscriptstyle V}(1)=0. \end{aligned}$$

#### Lemma 2.6

(Covariance structure of $\mathcal {S}_t$ [1, Lemma 2.3]). As $u\rightarrow \infty$,

- $$\widetilde{\mathrm{Var}}[\mathcal {S}_{t}] = u^{\tau -3}I_{\scriptscriptstyle V}(t/u) + O(1 + t |\theta ^* - \theta _u^*| u^{\tau -4})$$ uniformly in $$t\in [0,u]$$.
- $$\widetilde{\mathrm{Var}}[\mathcal {S}_{t}-\mathcal {S}_u] = u^{\tau -3}J_{\scriptscriptstyle V}(t/u) + O((u-t)u^{-1} + (u-t) |\theta ^* - \theta _u^*| u^{\tau -4})$$ uniformly in $$t\in [0,u]$$.
- $$\widetilde{\mathrm{Cov}}[\mathcal {S}_t,\mathcal {S}_u-\mathcal {S}_t] = -u^{\tau -3}G_{\scriptscriptstyle V}(t/u)+ O((u-t)u^{-1} + (u-t) |\theta ^* - \theta _u^*| u^{\tau -4})$$ uniformly in $$t\in [0,u]$$.

The next result bounds the Laplace transform of the couple $(\mathcal {S}_t,\mathcal {S}_u)$:

#### Proposition 2.7

(Joint moment generating function of $(\mathcal {S}_t,\mathcal {S}_u)$ [1, Proposition 2.4]). (a) As $u\rightarrow \infty$,

2.26 $$\begin{aligned} \widetilde{\mathbb {E}}\Bigg [{\mathrm {e}}^{ \lambda \frac{\mathcal {S}_{t}-\widetilde{\mathbb {E}}[\mathcal {S}_t]}{\sqrt{I_{\scriptscriptstyle V}(t/u) u^{\tau -3}}}}\Bigg ] = {\mathrm {e}}^{{1\over 2}\lambda ^2+ \Theta }, \end{aligned}$$

where $|\Theta |\le o_u(1)$ as $u\rightarrow \infty$ uniformly in $t\in [u/2,u]$ and $\lambda$ in a compact set.

(b) Fix $\varepsilon >0$ small. As $u\rightarrow \infty$, for any $\lambda _1, \lambda _2 \in \mathbb {R}$,

2.27 $$\begin{aligned} \widetilde{\mathbb {E}}\Bigg [{\mathrm {e}}^{ \lambda _1 {\mathcal {S}_{t} -\widetilde{\mathbb {E}}[\mathcal {S}_t] \over \sqrt{I_{\scriptscriptstyle V}(t/u) u^{\tau -3}}} + \lambda _2 {\mathcal {S}_{u} - \mathcal {S}_t - \widetilde{\mathbb {E}}[\mathcal {S}_u - \mathcal {S}_t] \over \sqrt{J_{\scriptscriptstyle V}(t/u)u^{\tau -3}}}}\Bigg ] = {\mathrm {e}}^{{1\over 2}\lambda _1^2 + {1\over 2}\lambda _2^2 - \lambda _1\lambda _2{G_{\scriptscriptstyle V}(t/u)\over I_{\scriptscriptstyle V}(t/u)J_{\scriptscriptstyle V}(t/u)} + \Theta }, \end{aligned}$$

where $|\Theta |\le o_u(1) + O(t^{3(3-\tau )/2})$ uniformly in $t\in [\varepsilon ,u-u^{-(\tau -5/2)}]$ and $\lambda _1, \lambda _2$ in a compact set.

Combine Proposition 2.7 and $u\widetilde{\mathbb {E}}[\mathcal {S}_u]=o(1)$ (see [1, Lemma 4.1]) to show that $u^{-(\tau -3)/2}\mathcal {S}_u$ converges to a normal distribution with mean 0 and variance $I_{\scriptscriptstyle V}(1)$. Moreover, as we see below, the density of $\mathcal {S}_{u}$ close to zero behaves like $\left( 2\pi I_{\scriptscriptstyle V}(1)\right) ^{-1/2}u^{-(\tau -3)/2}$:

#### Proposition 2.8

(Density of $\mathcal {S}_u$ near zero [1, Proposition 2.5]) Uniformly in $s=o(u^{(\tau -3)/2})$, the density $\widetilde{f}_{\mathcal {S}_u}$ of $\mathcal {S}_u$ satisfies

2.28 $$\begin{aligned} \widetilde{f}_{\mathcal {S}_u}(s)=B u^{-(\tau -3)/2}(1+o(1)), \end{aligned}$$

with $B= \left( 2\pi I_{\scriptscriptstyle V}(1)\right) ^{-1/2}$ and $I_{\scriptscriptstyle V}(p)$ defined in (2.21). Moreover, $\widetilde{f}_{\mathcal {S}_t}(s)$ is uniformly bounded by a constant times $u^{-(\tau -3)/2}$ for all *s*, *u* and $t \in [u/2,u]$.

There are three more results from [1] that will be used in this paper. The first is a description of the distribution of the indicator processes $(\mathcal {I}_i(t))_{t\ge 0}$ under the measure $\widetilde{\mathbb {P}}$. Since our indicator processes $(\mathcal {I}_i(t))_{t\ge 0}$ are *independent*, this property also holds under the measure $\widetilde{\mathbb {P}}$:

#### Lemma 2.9

(Indicator processes under the tilted measure [1, Lemma 4.2]) Under the measure $\widetilde{\mathbb {P}}$, the distribution of the indicator processes $(\mathcal {I}_i(t))_{t\ge 0}$ is that of independent indicator processes. More precisely,

2.29 $$\begin{aligned} \mathcal {I}_i(t)=\mathbb {1}_{\{T_i\le t\}}, \end{aligned}$$

where $(T_i)_{i\ge 2}$ are independent random variables with distribution

2.30 $$\begin{aligned} \widetilde{\mathbb {P}}(T_i\le t) = {\left\{ \begin{array}{ll} \frac{{\mathrm {e}}^{\theta c_iu}\left( 1-{\mathrm {e}}^{-c_i t}\right) }{{\mathrm {e}}^{\theta c_iu}\left( 1-{\mathrm {e}}^{-c_iu}\right) +{\mathrm {e}}^{-c_iu}} &{}\text {for } t \le u; \\ \frac{{\mathrm {e}}^{\theta c_iu}\left( 1-{\mathrm {e}}^{-c_iu}\right) +\left( {\mathrm {e}}^{-c_iu}-{\mathrm {e}}^{-c_i t}\right) }{{\mathrm {e}}^{\theta c_iu}\left( 1-{\mathrm {e}}^{-c_iu}\right) +{\mathrm {e}}^{-c_iu}} &{}\text {for } t>u. \end{array}\right. } \end{aligned}$$

The second lemma describes what happens to the variances for small *p* or for *p* close to 1:

#### Lemma 2.10

(Asymptotic variance near extremities [1, Lemma 4.3(b)]). As $p\rightarrow 1$, $J_{\scriptscriptstyle V}(p) = -(1-p)J_{\scriptscriptstyle V}'(1)(1+o(1))$ with $J_{\scriptscriptstyle V}'(1)<0$, while, as $p\rightarrow 0$,

2.31 $$\begin{aligned} I_{\scriptscriptstyle V}(p) = p^{\tau -3} \mathcal I_{\scriptscriptstyle V} (1+o(1)), \qquad \text{ with } \qquad \mathcal I_{\scriptscriptstyle V}= (\tau -1) \int _0^\infty (1 - {\mathrm {e}}^{-y} ) {\mathrm {e}}^{-y} \frac{ dy }{ y^{\tau - 2} }. \end{aligned}$$

Consequently, there exist $0<\underline{c}<\overline{c}<\infty$ such that, for every $p\in [0,\varepsilon ]$ with $\varepsilon >0$ sufficiently small,

2.32 $$\begin{aligned} \underline{c} p^{\tau -3} \le I_{\scriptscriptstyle V}(p) \le \overline{c} p^{\tau -3}. \end{aligned}$$

We finally rely on the following corollary that allows us to compute sums that we will encounter frequently:

#### Corollary 2.11

(Replacing sums by integrals in general [1, Corollary 3.3]). For every $a\in {\mathbb {R}}, a > \tau -1$ and $b>0$, there exists a constant *c*(*a*, *b*) such that

2.33 $$\begin{aligned} \sum _{i=2}^{\infty } c_i^a {\mathrm {e}}^{-b c_i u}=c(a,b) u^{\tau -a-1}(1+o(1)). \end{aligned}$$

### No Early Hits and Middle Ground

In this section, we prove that the tilted process is unlikely to hit 0 until a time that is very close to *u*. We start by investigating the early hits.

**No early hits** In this step, we prove that it is unlikely that the process hits zero early on, i.e., in the first time interval $[0,\varepsilon ]$ for some $\varepsilon >0$ sufficiently small. In its statement, we write $0\in \mathcal {S}_{[0,t]}$ for the event that $\{\mathcal {S}_s=0\}$ for some $s\in [0,t]$, so that $\mathbb {P}(H_1(0)>u)=\mathbb {P}(0\not \in \mathcal {S}_{[0,u]})$.

#### Lemma 2.12

(No early hits). For every $u\in [0,\infty )$, as $\varepsilon \downarrow 0$,

2.34 $$\begin{aligned} \mathbb {P}(0\in \mathcal {S}_{[0,\varepsilon ]},\mathcal {S}_{u}>0) =o_\varepsilon (1)\mathbb {P}(\mathcal {S}_{u}>0), \end{aligned}$$

where $o_\varepsilon (1)$ denotes a function that converges to zero as $\varepsilon \downarrow 0$, uniformly in *u*.

The proof of Lemma 2.12 follows from a straightforward application of the FKG-inequality for independent random variables (see [19], or [20, Theorem 2.4, p. 34]). The standard versions of the FKG-inequality hold for independent indicator random variables, and in our case we need it for independent exponentials. It is not hard to prove that the FKG-inequality we need holds by an approximation argument.

#### Proof

We note that the process $(\mathcal {S}_t)_{t\ge 0}$ is a deterministic function of the exponential random variables $(T_i)_{i\ge 2}$ (recall (1.15), (1.17) and (1.18)). Now, the event $\{0\in \mathcal {S}_{[0,\varepsilon ]}\}$ is *increasing* in terms of the random variables $(T_i)_{i\ge 2}$ (use that $\mathcal {S}_t$ only has positive jumps). Here we say that an event *A* is increasing when, if *A* occurs for a realization $(t_i)_{i\ge 2}$ of $(T_i)_{i\ge 2}$, and if $(t_i')_{i\ge 2}$ is coordinatewise larger than $(t_i)_{i\ge 2}$, then *A* also occurs for $(t_i')_{i\ge 2}$. Clearly, the event $\{\mathcal {S}_{u}>0\}$ is *decreasing* (for a definition, change the role of $t_i$ and $t'_i$ in the definition of an increasing event), so that the FKG-inequality implies that these events are negatively correlated:

2.35 $$\begin{aligned} \mathbb {P}(0\in \mathcal {S}_{[0,\varepsilon ]},\mathcal {S}_{u}>0) \le \mathbb {P}(0\in \mathcal {S}_{[0,\varepsilon ]})\mathbb {P}(\mathcal {S}_{u}>0). \end{aligned}$$

We conclude the proof by noting that $\mathbb {P}(0\in \mathcal {S}_{[0,\varepsilon ]})=o_\varepsilon (1)$ independently of *u*. $\square$

The key to our proof of Theorem 1.4 will be to show that $\mathbb {P}(H_1(0)>u)=\Theta (\mathbb {P}(\mathcal {S}_{u}>0))$, so that Lemma 2.12 and the known asymptotics of $\mathbb {P}(\mathcal {S}_{u}>0)$ imply that it is unlikely to have an early hit of zero.

**No middle ground** By (2.4) (recall that $\phi (u)=\phi (u;\theta )$ with $\theta =\theta ^{*}_u$), Lemma 2.12 and Theorem 2.1,

2.36 $$\begin{aligned}&\mathbb {P}(H_1(0)>u) = \phi (u) \widetilde{\mathbb {E}}\left[ {\mathrm {e}}^{-\theta u \mathcal {S}_u} \mathbb {1}_{\{\mathcal {S}_{[0,u]}>0\}}\right] \nonumber \\&\quad =\phi (u) \widetilde{\mathbb {E}}\left[ {\mathrm {e}}^{-\theta u \mathcal {S}_u} \mathbb {1}_{\{\mathcal {S}_{[\varepsilon ,u]}>0\}}\right] +\phi (u) u^{-(\tau -1)/2} o_{\varepsilon }(1). \end{aligned}$$

For $M>0$ arbitrarily fixed, we split

2.37 $$\begin{aligned}&\mathbb {P}(H_1(0)>u) = \phi (u) \widetilde{\mathbb {E}}\left[ {\mathrm {e}}^{-\theta u \mathcal {S}_u} \mathbb {1}_{\{\mathcal {S}_{[\varepsilon ,u]}>0, \mathcal {S}_u\in [0,M/u]\}}\right] \nonumber \\&\quad +\, \phi (u) \widetilde{\mathbb {E}}\left[ {\mathrm {e}}^{-\theta u \mathcal {S}_u} \mathbb {1}_{\{\mathcal {S}_{[\varepsilon ,u]}>0, \mathcal {S}_u>M/u\}}\right] +\phi (u)u^{-(\tau -1)/2}o_{\varepsilon }(1). \end{aligned}$$

By Proposition 2.8, we can bound

2.38 $$\begin{aligned} \widetilde{\mathbb {E}}\left[ {\mathrm {e}}^{-\theta u \mathcal {S}_u} \mathbb {1}_{\{\mathcal {S}_{[\varepsilon ,u]}>0, \mathcal {S}_u>M/u\}}\right]&\le \widetilde{\mathbb {E}}\left[ {\mathrm {e}}^{-\theta u \mathcal {S}_u} \mathbb {1}_{\{\mathcal {S}_u>M/u\}}\right] \le \int _{M/u}^{\infty } {\mathrm {e}}^{-\theta u v}\tilde{f}_{\mathcal {S}_u}(v)dv \nonumber \\&\le O(u^{-(\tau -3)/2}) \int _{M/u}^{\infty } {\mathrm {e}}^{-\theta u v}dv=O(u^{-(\tau -1)/2}){\mathrm {e}}^{-\theta M}. \end{aligned}$$

As a result, we arrive at

2.39 $$\begin{aligned}&\mathbb {P}(H_1(0)>u) = \phi (u) \widetilde{\mathbb {E}}\left[ {\mathrm {e}}^{-\theta u \mathcal {S}_u} \mathbb {1}_{\{\mathcal {S}_{[\varepsilon ,u]}>0, \mathcal {S}_u\in [0,M/u]\}}\right] \nonumber \\&\quad + \,\phi (u)u^{-(\tau -1)/2}o_{\scriptscriptstyle M}(1)+\phi (u)u^{-(\tau -1)/2}o_{\varepsilon }(1), \end{aligned}$$

where $o_{\scriptscriptstyle M}(1)$ denotes a quantity *c*(*M*, *u*) such that $\limsup _{M\rightarrow \infty } \limsup _{u\rightarrow \infty } c(M,u)=0$.

We continue to prove that the dominant contribution to the expectation of the right-hand side of (2.39) originates from paths that remain positive until time $u-t$ for $t=Tu^{-(\tau -2)}$, with $T>0$ arbitrarily fixed.

#### Proposition 2.13

(No middle ground). Fix $\varepsilon >0$. For every $u\in [0,\infty )$ and $\varepsilon , M>0$ fixed,

2.40 $$\begin{aligned} \widetilde{\mathbb {P}} (0\in \mathcal {S}_{[\varepsilon ,u-Tu^{-(\tau -2)}]}, \mathcal {S}_u \in [0,M/u]) \le o_{\scriptscriptstyle T}(1)u^{-(\tau -1)/2}, \end{aligned}$$

where we recall that $o_{\scriptscriptstyle T}(1)$ denotes a quantity *c*(*T*, *u*) such that $\lim _{T\rightarrow \infty } \limsup _{u\rightarrow \infty }c(T,u)=0$.

We prove Proposition 2.13 in Sect. 3.

By (2.39) and Proposition 2.13,

2.41 $$\begin{aligned}&\mathbb {P}(H_1(0)>u) = \phi (u) \widetilde{\mathbb {E}}\left[ {\mathrm {e}}^{-\theta u \mathcal {S}_u} \mathbb {1}_{\{\mathcal {S}_{[u-T u^{-(\tau -2)},u]}>0\}}\right] \nonumber \\&\quad + \, \phi (u)u^{-(\tau -1)/2}\big [o_{\varepsilon }(1)+o_{\scriptscriptstyle M}(1)+o_{\scriptscriptstyle T}(1)\big ]. \end{aligned}$$

Since $\varepsilon$, *M* and *T* are arbitrary, it now suffices to identify the asymptotics of the expectation appearing on the right-hand side of (2.41).

### Remaining Positive Near the End

To prove Theorem 1.4, by Proposition 2.3 and Eq. (2.41), it suffices to prove that, with $\gamma =(\tau -1)/2$,

2.42 $$\begin{aligned} \widetilde{\mathbb {E}}\left[ {\mathrm {e}}^{-\theta u \mathcal {S}_u}\mathbb {1}_{\{\mathcal {S}_{[u-Tu^{-(\tau -2)},u]}>0, \mathcal {S}_u \in [0,M/u]\}}\right] =(A+o_{\scriptscriptstyle M}(1)+o_{\scriptscriptstyle T}(1))u^{-\gamma }(1+o(1)),\qquad \quad \end{aligned}$$

where $T>0$ fixed. In the above expectation, we see two terms. The term ${\mathrm {e}}^{-\theta u \mathcal {S}_u}$ forces $\mathcal {S}_u$ to be small, more precisely, $\mathcal {S}_u=\Theta (1/u)$ for *u* large, while the term $\mathbb {1}_{\{\mathcal {S}_{[u-Tu^{-(\tau -2)},u]}>0\}}$ forces the path to remain positive until time *u*. We now study these two effects.

We start by highlighting the ideas behind the analysis of the process $(\mathcal {S}_t)_{t\in [u-T u^{-(\tau -2)},u]}$. Comparing Theorem 1.4 to Theorem 2.1, we see that they are identical, except for the precise constant, which is *A* in Theorem 1.4 and $D>A$ in Theorem 2.1. This difference is due to the fact that, conditionally on $\mathcal {S}_u>0$, the process has a probability of not hitting zero in the interval $[u-T u^{-(\tau -2)},u]$ that is strictly positive and bounded away from zero. In order to analyse this probability, we identify the scaling limit of the process $(u \mathcal {S}_{u-tu^{-(\tau -2)}} - u\mathcal {S}_u)_{t \ge 0}$ as $u\rightarrow \infty$ *conditionally on* $u\mathcal {S}_u=v$, and relate it to a certain Lévy process. The parameter *A* / *D* is closely related to the probability that this limiting process is bounded below by $-v$, integrated over *v*. Let us now give the details.

In order to investigate the probability that $\mathcal {S}_{[u-Tu^{-(\tau -2)},u]}>0$, we proceed as follows. Let

2.43 $$\begin{aligned} \mathcal {J}(u)=\{j:\mathcal {I}_j(u)=1\} \end{aligned}$$

denote the set of indices for which $T_j\le u$. We condition on the set $\mathcal {J}(u)$. Note that $\mathcal {S}_u$ is measurable with respect to $\mathcal {J}(u)$. We now rewrite $\mathcal {S}_{u-t}$ in a convenient form. For this, recall (1.21) and write

2.44 $$\begin{aligned} \mathcal {S}_{u-t}&=\frac{t}{u}+\frac{u-t}{u}\mathcal {S}_u +\sum _{i=2}^{\infty } c_i\left[ \mathcal {I}_i(u-t)-\frac{u-t}{u}\mathcal {I}_i(u)\right] \nonumber \\&=\frac{t}{u}+\frac{u-t}{u}\mathcal {S}_u -\sum _{i=2}^{\infty } c_i\left[ \mathbb {1}_{\{T_i\in (u-t,u]\}}-\frac{t}{u}\mathcal {I}_i(u)\right] . \end{aligned}$$

Thus, with

2.45 $$\begin{aligned} Q_u(t)&\equiv u \mathcal {S}_{u-t} - t - (u-t) \mathcal {S}_u =-\sum _{i=2}^{\infty } c_i[u\mathbb {1}_{\{T_i\in (u-t,u]\}}-t\mathcal {I}_i(u)], \end{aligned}$$

we have that $\mathcal {S}_{u-t}>0$ precisely when $Q_u(t) > -t-(u-t)\mathcal {S}_u$. We rewrite

2.46 $$\begin{aligned} Q_u(t)=-\sum _{i\in \mathcal {J}(u)} c_i[u\mathbb {1}_{\{T_i\in (u-t,u]\}}-t]. \end{aligned}$$

Note that, for any $t=o(u)$,

2.47 $$\begin{aligned} \widetilde{\mathbb {E}}[{\mathrm {e}}^{-\theta u \mathcal {S}_u}\mathbb {1}_{\{\mathcal {S}_{[u-t,u]}>0\}}]&= \frac{1}{u}\int _0^{\infty } {\mathrm {e}}^{-\theta v} \widetilde{\mathbb {P}}\big (\mathcal {S}_{[u-t,u]}>0\mid u\mathcal {S}_u=v\big ) \widetilde{f}_{\mathcal {S}_u}(v/u)dv \nonumber \\&= \frac{1}{u}\int _0^{\infty } {\mathrm {e}}^{-\theta v} \widetilde{\mathbb {P}}\big (Q_u(s)> -v-s+sv/u \,\forall s\in [0,t]\mid u\mathcal {S}_u=v\big ) \nonumber \\&\widetilde{f}_{\mathcal {S}_u}(v/u)dv. \end{aligned}$$

We aim to use dominated convergence on the above integral, and we start by proving pointwise convergence. By Proposition 2.8, $\widetilde{f}_{\mathcal {S}_u}(v/u)=B u^{-(\tau -3)/2}(1+o(1))$ pointwise in *v* (in fact, even when $v=o(u^{(\tau -1)/2})$). This leads us to study, for all $v>0$,

2.48 $$\begin{aligned} g_{u,t}(v)\equiv \widetilde{\mathbb {P}}\big (Q_u(s)> -v-s+sv/u \,\forall s\in [0,t]\mid u\mathcal {S}_u=v\big ). \end{aligned}$$

We split

2.49 $$\begin{aligned} Q_u(t)=A_u(t)-B_u(t), \end{aligned}$$

where

2.50 $$\begin{aligned}&B_u(t)\equiv u\sum _{i\in \mathcal {J}(u)} c_i\left[ \mathbb {1}_{\{T_i\in (u-t,u]\}}-\widetilde{\mathbb {P}}(T_i>u-t \mid T_i\le u)\right] , \nonumber \\&\quad A_u(t)\equiv -\sum _{i\in \mathcal {J}(u)} c_i\left[ u\widetilde{\mathbb {P}}(T_i>u-t \mid T_i\le u)-t\right] . \end{aligned}$$

Thus, $(A_u(t))_{t\in [0,u]}$ is *deterministic* given $\mathcal {J}(u)$, while $(B_u(t))_{t\in [0,u]}$ is *random* given $\mathcal {J}(u)$. The main result for the near-end regime is the following proposition, which proves that $g_u(v)$ converges pointwise.

#### Proposition 2.14

(Weak conditional convergence of time-reversed process). (a) As $u\rightarrow \infty$, conditionally on $u\mathcal {S}_{u}=v$,

2.51 $$\begin{aligned} (A_u(t u^{-(\tau -2)}))_{t\ge 0}{\mathop {\longrightarrow }\limits ^{d}}(\kappa t)_{t\ge 0}, \end{aligned}$$

where $\kappa \in (0,\infty )$ is given by

2.52 $$\begin{aligned} \kappa =\int _0^{\infty } x^{-\alpha } \frac{{\mathrm {e}}^{\theta x^{-\alpha }}{\mathrm {e}}^{-x^{-\alpha }}}{{\mathrm {e}}^{\theta x^{-\alpha }}(1-{\mathrm {e}}^{-x^{-\alpha }})+{\mathrm {e}}^{-x^{-\alpha }}} \big [{\mathrm {e}}^{x^{-\alpha }}-1-x^{-\alpha } \big ]dx. \end{aligned}$$

(b) As $u\rightarrow \infty$, conditionally on $u\mathcal {S}_{u}=v$,

2.53 $$\begin{aligned} (B_u(tu^{-(\tau -2)}))_{t\ge 0}{\mathop {\longrightarrow }\limits ^{d}}(L_t)_{t\ge 0}, \end{aligned}$$

where $(-L_t)_{t\ge 0}$ is a Lévy process with no positive jumps and with Laplace transform

2.54 $$\begin{aligned} \mathbb {E}\left[ {\mathrm {e}}^{a (-L_s)}\right] ={\mathrm {e}}^{s \int _{-\infty }^0 \left( {\mathrm {e}}^{a z}-1-a z\right) \Pi (dz)}, \quad a \ge 0, \end{aligned}$$

and characteristic measure

2.55 $$\begin{aligned} \Pi (dz)=(\tau -1)\frac{(-z)^{-(\tau -1)}{\mathrm {e}}^{-\theta z}}{{\mathrm {e}}^{-\theta z}(1-{\mathrm {e}}^z) +{\mathrm {e}}^z} {\mathrm {e}}^z dz. \end{aligned}$$

Proposition 2.14 is proved in Sect. 5, and determines the precise constant *A* from (1.25), as we now explain in more detail.

We proceed by investigating some properties of the supremum of the Lévy process from (2.53) that we need later on. Note in particular that the distribution of $L_s$ in (2.54) does not depend on *v*. With a slight abuse of notation, also the probability law describing the limiting process $(L_s)_{s\ge 0}$ shall be denoted by $\mathbb {P}$.

#### Lemma 2.15

(Supremum of the Lévy process). Let $I_\infty \equiv \inf _{t \ge 0} (-L_t+\kappa t)$. Then

2.56 $$\begin{aligned} \mathbb {P}(I_\infty \ge -v) = \mathcal {W}(v)/\mathcal {W}(\infty ), \end{aligned}$$

where $\mathcal {W}:[0,\infty ) \rightarrow [0,\infty )$ is the unique continuous increasing function that has Laplace transform

2.57 $$\begin{aligned} \int _0^\infty {\mathrm {e}}^{-a x} \mathcal {W}(x) dx = \frac{1}{\psi (a)}, \quad a>\Psi (0), \end{aligned}$$

where the Laplace exponent $\psi$ is given by $\mathbb {E}[{\mathrm {e}}^{a (\kappa t-L_t)}]={\mathrm {e}}^{t\psi (a)}$ and is computed in (2.58) below, while $\Psi (0)$ is the largest solution of the equation $\psi (a)=0$, and $\mathcal {W}(\infty )=1/\psi '(0)=1/\kappa$ is a constant.

#### Proof

We rewrite (2.54) to see that $X_s \equiv -L_s+\kappa s$ is a Lévy process with no positive jumps and Laplace exponent

2.58 $$\begin{aligned} \psi (a)&= \kappa a + \int _{(-\infty ,0)} \left( {\mathrm {e}}^{az}-1-az \right) \Pi (dz) \nonumber \\&= \beta ' a + \int _{(-\infty ,0)} \left( {\mathrm {e}}^{az}-1-az \mathbb {1}_{\{z>-1\}} \right) \Pi (dz) \end{aligned}$$

with

2.59 $$\begin{aligned} \beta '&= \kappa + \int _{(-\infty ,0)} (-z) \mathbb {1}_{\{z \le -1\}} \Pi (dz) > 0 \end{aligned}$$

as defined in [5, Sect. VII.1]. Indeed, recall from [5, Sect. VII.1] that $\mathbb {E}[{\mathrm {e}}^{aX_s}] = {\mathrm {e}}^{s \psi (a)}$ and note that our $\beta '$ corresponds to *a* in [5]. Also note from (2.52) that $\kappa >0$. Thus $\psi '(0+) = \kappa >0$ and [5, Corollary 2(ii) in Sect. VII.1] yields that $X_s$ drifts to $\infty$ (for a definition, see [5, Theorem 12(ii) in Sect. VI.3]). This in turn implies (see [5, Proof of Theorem 8, in Sect. VII.2])

2.60 $$\begin{aligned} \mathbb {P}(I_\infty \ge -v) = \mathcal {W}(v)/\mathcal {W}(\infty ), \end{aligned}$$

where $\mathcal {W}$ is given in the statement of [5, Theorem 8, in Sect. VII.2]. For the definition of $\Psi$ see before [5, Theorem 1 of Sect. VII.1]. Also note from the second equation of the proof of [5, Proof of Theorem 8, in Sect. VII.2] that $\mathcal {W}(\infty )>0$. To see that $\mathcal {W}(\infty )=1/\psi (0)$, note that if $a\downarrow 0$,

2.61 $$\begin{aligned} \int _0^\infty {\mathrm {e}}^{-a x} \mathcal {W}(x) dx= \frac{\mathcal {W}(\infty )}{a (1+o(1))}. \end{aligned}$$

Now, $\psi (0)=0$, so that $1/\psi (a)=1/(a\psi '(0))(1+o(1))$ as $a\downarrow 0$, which identifies $\mathcal {W}(\infty )=1/\psi '(0)=1/\kappa$. $\square$

By Proposition 2.14 and the continuity of $\mathcal {W}$ in Lemma 2.15, with $\mathcal {M}_T=\sup _{0\le s\le T} (L_s-\kappa s)$ for each $v\ge 0$ and for $t=Tu^{-(\tau -2)}$, for $u \rightarrow \infty$,

2.62 $$\begin{aligned} g_{u,t}(v)\rightarrow g_T(v)\equiv \mathbb {P}\big (\mathcal {M}_T \le v\big ). \end{aligned}$$

Further, as $$T\rightarrow \infty $$,

2.63 $$\begin{aligned} g_T(v)\downarrow g(v)\equiv \mathbb {P}\left( \sup _{0\le s < \infty } (L_s-\kappa s)\le v\right) =\frac{\mathcal {W}(v)}{\mathcal {W}(\infty )}. \end{aligned}$$

Now we are ready to complete the proofs of our main results.

### Completion of the Proofs

**Completion of the Proof of Theorem**1.4 We start by completing the proof of Theorem 1.4. Recall that it remains to prove (2.42) with $\gamma =(\tau -1)/2$. By (2.47) and (2.48), we need to compute

2.64 $$\begin{aligned} \widetilde{\mathbb {E}}[{\mathrm {e}}^{-\theta u \mathcal {S}_u}\mathbb {1}_{\{\mathcal {S}_{[u-t,u]}>0, \mathcal {S}_u\in [0,M/u]\}}] = \frac{1}{u^{(\tau -1)/2}}\int _0^{M} {\mathrm {e}}^{-\theta v} g_{u,t}(v)\big [u^{(\tau -3)/2}\widetilde{f}_{\mathcal {S}_u}(v/u)\big ]dv,\nonumber \\ \end{aligned}$$

where $t=Tu^{-(\tau -2)}$. A similar problem was encountered in [1, Proof of Theorem 1.1], which is restated here as Theorem 2.1, apart from the fact that there the function $g_{u,t}(v)$ was absent.

We wish to use bounded convergence. For this, we note that $u^{(\tau -3)/2}\widetilde{f}_{\mathcal {S}_u}(v/u)\rightarrow B$ by Proposition 2.8 for each *v* (in fact, for all $v=o(u)$), while, by (2.62)–(2.63), $g_{u,t}(v)\rightarrow g_T(v)$, which, in turn, converges to *g*(*v*) as $T\rightarrow \infty$. Further, since $g_{u,t}(v)\le 1$ and $u^{(\tau -3)/2}\widetilde{f}_{\mathcal {S}_u}(v/u)$ is uniformly bounded (see Proposition 2.8), the integrand ${\mathrm {e}}^{-\theta v}g_{u,t}(v)\big [u^{(\tau -3)/2}\widetilde{f}_{\mathcal {S}_u}(v/u)\big ]$ is uniformly bounded by a constant. Thus, by the Bounded Convergence Theorem,

2.65 $$\begin{aligned} \widetilde{\mathbb {E}}\left[ {\mathrm {e}}^{-\theta u \mathcal {S}_u}\mathbb {1}_{\{\mathcal {S}_{[u-t,u]}>0, \mathcal {S}_u\in [0,M/u]\}}\right]&=\frac{B}{u^{(\tau -1)/2}}\int _0^M {\mathrm {e}}^{-\theta v} g_T(v)dv(1+o(1)) \nonumber \\&=\frac{B}{u^{(\tau -1)/2}}\int _0^M {\mathrm {e}}^{-\theta v} g(v)dv(1+o(1)+o_{\scriptscriptstyle T}(1)). \end{aligned}$$

This identifies (recall (2.42), (2.56), (2.57) and (2.62))

2.66 $$\begin{aligned} A=B\int _0^{\infty } {\mathrm {e}}^{-\theta v} g(v)dv =B\int _0^{\infty } {\mathrm {e}}^{-\theta v} \mathbb {P}\big (\mathcal {M}\le v\big )dv=\frac{B}{\theta } \mathbb {E}[{\mathrm {e}}^{-\theta \mathcal {M}}]=\frac{B\psi '(0)}{\psi (\theta )}.\qquad \quad \end{aligned}$$

Recall that *D* is the constant appearing in Theorem 2.1. Since $D=B/\theta$ by [1, (7.4)] and $\mathbb {P}\big (\mathcal {M}\le v\big )<1$ for every *v*, we also immediately obtain that $A\in (0,D)$. and completes the proof of Theorem 1.4. $\square$

**Path properties: Proof of Theorem**1.5 We bound, using that $\{H_1(0)>u\}\subseteq \{\mathcal {S}_u>0\}$,

2.67 $$\begin{aligned}&\mathbb {P}\Big (\sup _{p\in [0,1]} |\mathcal {S}_{pu}-u^{\tau -2}I_{\scriptscriptstyle E}(p)|>\varepsilon u^{\tau -2}\mid H_1(0)>u\Big ) \nonumber \\&\qquad \le \mathbb {P}\Big (\sup _{p\in [0,1]} |\mathcal {S}_{pu}-u^{\tau -2}I_{\scriptscriptstyle E}(p)|>\varepsilon u^{\tau -2}\mid \mathcal {S}_u>0\Big )\frac{\mathbb {P}(\mathcal {S}_u>0)}{\mathbb {P}(H_1(0)>u)}. \end{aligned}$$

By Theorems 2.1 and 1.4, the ratio of probabilities converges to $D/A\in (0,\infty )$, while, by Theorem 2.2, the conditional probability converges to 0. This completes the proof of Theorem 1.5. $\square$

**Completion of the Proof of the Main Theorem** We finally complete the proof of the scaling of the critical clusters in the Main Theorem using Theorem 1.4 and recalling (1.22). For this, we go back to the random graph setting. Let us start by giving some introduction.

The process $(\mathcal {S}_t)_{t\ge 0}$ in (1.21) arises when exploring a cluster in the Norros–Reittu random graph with weights $\varvec{w}(\lambda )$ defined in (1.6) and (1.12), as described informally in Sect. 1.2. Recall Theorem 1.3. Here $\mathcal {S}_t$ denotes the scaling limit of $n^{-1/(\tau -1)}=n^{-\alpha }$ times the number of vertices found at time $tn^{(\tau -2)/(\tau -1)}=tn^{\rho }$.

The key idea is that each time that a vertex, say $j\in [n]$, is being explored, we have a chance $(1+\lambda n^{-(\tau -3)/(\tau -1)})w_iw_j/\ell _n$ that the edge to the vertex *i* with the $i\hbox {th}$ largest weight is present. As it turns out (see e.g., [6, Lemma 1.3]), the vertices are found in a size-biased reordered way, meaning that the $k\hbox {th}$ vertex found is $v_{\scriptscriptstyle (k)}$, where (here the factor $(1+\lambda n^{-(\tau -3)/(\tau -1)})$ cancels)

2.68 $$\begin{aligned} \mathbb {P}\big (v_{\scriptscriptstyle (k)}=j\mid v_{\scriptscriptstyle (1)}, \ldots , v_{\scriptscriptstyle (k-1)}\big )=\frac{w_j}{\sum _{l\not \in \{v_{\scriptscriptstyle (1)}, \ldots , v_{\scriptscriptstyle (k-1)}\}} w_l}. \end{aligned}$$

Thus, the average weight of the $k\hbox {th}$ vertex found is

2.69 $$\begin{aligned} \mathbb {E}\big [w_{v_{\scriptscriptstyle (k)}}(1+\lambda n^{-(\tau -3)/(\tau -1)}) \mid v_{\scriptscriptstyle (1)}, \ldots , v_{\scriptscriptstyle (k-1)}\big ] \approx \sum _{j\in [n]}\frac{w_j^2}{\ell _n}\approx \frac{\mathbb {E}[W^2]}{\mathbb {E}[W]}=\nu =1, \end{aligned}$$

which informally corresponds to the graph being close to critical (as made more precise in [7]). By (2.68), the probability that at the $k\hbox {th}$ exploration we find the vertex *i* with the $i\hbox {th}$ largest weight is close to $w_i/\ell _n$. By (1.10) and (1.6),

2.70 $$\begin{aligned} w_i\approx (c_{\scriptscriptstyle F}n/i)^{1/(\tau -1)}, \end{aligned}$$

so the probability of finding *i* is close to $(c_{\scriptscriptstyle F}/i)^{1/(\tau -1)} n^{(2-\tau )/(\tau -1)}/\mathbb {E}[D]\approx a i^{-1/(\tau -1)}n^{-\rho }$ by the definitions below (1.15) and (1.13). If this occurs, then the number of vertices added to the exploration process is close to $w_i(1+\lambda n^{-(\tau -3)/(\tau -1)})\approx (c_{\scriptscriptstyle F}/i)^{1/(\tau -1)}n^{1/(\tau -1)}=bi^{-1/(\tau -1)} n^{\alpha }.$ Further, the probability that vertex *i* is *not* found in the time interval $[0,t]n^{\rho }$ is close to ${\mathrm {e}}^{-t a i^{-1/(\tau -1)}}=\mathbb {P}(\mathcal {I}_i(t)=0)$. It is not hard to see that these events are weakly dependent, so that the scaling limits of the times that the high-weight vertices are found are close to *independent* exponential random variables with rate $a i^{-1/(\tau -1)}$. This explains the random variables arising in (1.21). The restriction to $i\ge 2$ in (1.21) arises since we explore the cluster of vertex 1. The cluster is fully explored when there are no more active vertices waiting to be explored. This corresponds to $\mathcal {S}_t=0$ for the first time, which is $H_1^{a}(0)$ and explains the result in Theorem 1.3. Recall the informal description in Sect. 1.2 here as well.

Next, we claim that when a particularly *large* cluster is found, then, since the weight $w_1$ is the largest of all weights, the maximal cluster is whp the cluster of vertex 1. This explains why the asymptotics in the Main Theorem for the maximal cluster is *identical* to the asymptotics in Theorem 1.4 for the cluster of vertex 1. To make this heuristic precise, we show, in this section, that indeed it is unlikely for an unusually large cluster to be found that does not contain 1. We next make the ideas in this heuristic precise, by introducing the exploration process of the cluster of vertex *i* for a general $i\ge 1$.

Denote

2.71 $$\begin{aligned} \mathcal {S}_t^{\scriptscriptstyle (i)}=c_i+\tilde{\beta }_i t+\sum _{j=1:j\ne i}^{\infty } c_j [\mathcal {I}_j(t)-c_j t], \end{aligned}$$

where $\tilde{\beta }_i=(\lambda +\zeta )/(ab)-c_i^2$ (see [7, Remark 3.9] and recall $a',b',c'$ from above (1.21)). The intuition for the above formula is that

2.72 $$\begin{aligned} \mathcal {S}_t^{\scriptscriptstyle (i)}=\frac{\lambda +\zeta }{ab}t+\sum _{j\ge 1}^{\infty } c_j [\mathcal {I}_j(t)-c_j t], \end{aligned}$$

where we slightly abuse notation to now set $\mathcal {I}_i(0)=1$ for the process $(\mathcal {S}_t^{\scriptscriptstyle (i)})_{t\ge 0}$ since vertex *i* is almost surely in the cluster of vertex *i*. Since $(\mathcal {S}_t^{\scriptscriptstyle (i)})_{t\ge 0}$ describes the scaling limit of the exploration process of the cluster of vertex $i\ge 1$, while $\mathcal {I}_j(t)$ has the interpretation as the indicator that vertex *j* is found in the exploration before time *t*, it is reasonable to set $\mathcal {I}_i(0)=1$ for $(\mathcal {S}_t^{\scriptscriptstyle (i)})_{t\ge 0}$.^2^ Again recall the informal description of the exploration process in Sect. 1.2.

We continue to show that it is highly unlikely that the cluster of vertex *i* is large, while vertex 1 is not in it. For this, we define

2.73 $$\begin{aligned} H^{\scriptscriptstyle (i)}(0)=\inf \left\{ t\ge 0:\mathcal {S}_t^{\scriptscriptstyle (i)}=0\right\} . \end{aligned}$$

Then, $H_1(0)=H^{\scriptscriptstyle (1)}(0)$ and $H^{\scriptscriptstyle (i)}(0)$ denotes (an appropriate multiple of) the scaling limit of the cluster of vertex *i*, i.e., $n^{-\rho }|{\mathcal {C}}(i)|{\mathop {\longrightarrow }\limits ^{d}}a H^{\scriptscriptstyle (i)}(0)$, where ${\mathcal {C}}(i)$ denotes the connected component to which vertex *i* belongs to. Further, let ${\mathcal {C}}_{\scriptscriptstyle \le }(i)$ be the set ${\mathcal {C}}(i)$ if none of the vertices $j\in [i-1]=\{1,\ldots ,i-1\}$ belongs to ${\mathcal {C}}(i)$, and the empty set $\varnothing$ otherwise. We know from [7, (3.78)] and the scaling explained around (1.21) that $n^{-\rho }|{\mathcal {C}}(i)|{\mathop {\longrightarrow }\limits ^{d}}a \cdot H^{\scriptscriptstyle (i)}(0)$ for each $i\ge 1$ with $\rho =(\tau -2)/(\tau -1)$ (cf. (1.13)). Finally, denote

2.74 $$\begin{aligned} H_i(0)= {\left\{ \begin{array}{ll} 0 &{}\text {if }\exists j<i \text { such that } \mathcal {I}_j(H^{\scriptscriptstyle (i)}(0))=1; \\ H^{\scriptscriptstyle (i)}(0) &{}\text {otherwise.} \end{array}\right. } \end{aligned}$$

Then, by [7, (3.79)], $n^{-\rho }|{\mathcal {C}}_{\scriptscriptstyle \le }(i)| {\mathop {\longrightarrow }\limits ^{d}}a \cdot H_i(0)$. This provides us with the appropriate background to complete the proof of the Main Theorem.

We start with the lower bound. By construction, $\gamma _1(\lambda )\ge a \cdot H_1(0)$ (see [7, Theorems 1.1 and 2.1] and recall that ${\mathcal {C}}_{\scriptscriptstyle (i)}$ denotes the $i\hbox {th}$-largest connected component). Therefore,

2.75 $$\begin{aligned} \mathbb {P}(\gamma _1(\lambda )>au)\ge \mathbb {P}(H_1(0)>u), \end{aligned}$$

and thus the lower bound follows from Theorem 1.4.

For the upper bound, we use that (cf. [7, Theorems 1.1])

2.76 $$\begin{aligned} \mathbb {P}(\gamma _1(\lambda )>au)&= \lim _{n\rightarrow \infty } \mathbb {P}\left( \exists i:n^{-\rho }|{\mathcal {C}}_{\scriptscriptstyle \le }(i)|\ge au\right) \nonumber \\&=\lim _{K\rightarrow \infty }\lim _{n\rightarrow \infty } \mathbb {P}\left( \exists i\in [K]:n^{-\rho }|{\mathcal {C}}_{\scriptscriptstyle \le }(i)|\ge au\right) \nonumber \\&\le \lim _{K\rightarrow \infty } \lim _{n\rightarrow \infty } \sum _{i\in [K]} \mathbb {P}\left( n^{-\rho }|{\mathcal {C}}_{\scriptscriptstyle \le }(i)|\ge au\right) , \end{aligned}$$

Here we have used the fact that there are with high probability only finitely many clusters that are larger than $\varepsilon n^{\rho }$ (as proved in [7, Theorem 1.6]).

By the weak convergence of $n^{-\rho }|{\mathcal {C}}_{\scriptscriptstyle \le }(i)|$, it holds that $\lim _{n\rightarrow \infty } \mathbb {P}(n^{-\rho }|{\mathcal {C}}_{\scriptscriptstyle \le }(i)|\ge au)=\mathbb {P}(H_i(0)>u)$ for all $i\ge 1$, so that we arrive at

2.77 $$\begin{aligned} \mathbb {P}(\gamma _1(\lambda )>au)\le \mathbb {P}(H_1(0)>u) +\sum _{i\ge 2} \mathbb {P}(H_i(0)>u). \end{aligned}$$

The first term is the main term, and we prove that $\sum _{i\ge 2} \mathbb {P}(H_i(0)>u)=o(\mathbb {P}(H_1(0)>u))$ now.

For this, we note that

2.78 $$\begin{aligned} \mathbb {P}(H_i(0)>u)&=\mathbb {P}\left( \mathcal {I}_j(u)=0 \, \forall j\in [i-1], \mathcal {S}_{[0,u]}^{\scriptscriptstyle (i)}>0\right) \nonumber \\&=\mathbb {P}(\mathcal {I}_j(u)=0 \, \forall j\in [i-1])\mathbb {P}\left( \mathcal {S}_{[0,u]}^{\scriptscriptstyle (i)}>0\mid \mathcal {I}_j(u)=0 \, \forall j\in [i-1]\right) . \end{aligned}$$

We can rewrite, on the event $\{\mathcal {I}_j(u)=0 \, \forall j\in [i-1]\}$, and using that $c_1\ge c_i$ for every $i\ge 1$,

2.79 $$\begin{aligned} \mathcal {S}_{t}^{\scriptscriptstyle (i)}&=\tfrac{\lambda +\zeta }{ab}t+\sum _{j\ge i+1} c_j(\mathcal {I}_j(t)-c_jt) +c_i-\sum _{j=1}^i c_j^2t \nonumber \\&\le \tfrac{\lambda +\zeta }{ab}t+\sum _{j\ge i+1} c_j(\mathcal {I}_j(t)-c_jt) +c_1-\sum _{j=1}^i c_j^2t. \end{aligned}$$

Therefore,

2.80 $$\begin{aligned}&\mathbb {P}\left( \mathcal {S}_{[0,u]}^{\scriptscriptstyle (i)}>0\mid \mathcal {I}_j(u)=0 \, \forall j\in [i-1]\right) \nonumber \\&\quad \le \mathbb {P}\left( \tfrac{\lambda +\zeta }{ab}t+\sum _{j\ge i+1} c_j(\mathcal {I}_j(t)-c_jt) +c_1-\sum _{j=1}^i c_j^2t>0~\forall t\in [0,u]\right) \nonumber \\&\quad =\mathbb {P}\big (\mathcal {S}_{[0,u]}^{\scriptscriptstyle (1)}>0\mid \mathcal {I}_j(u)=0 \, \forall j\in [i]\setminus \{1\}\big ), \end{aligned}$$

where in the last equality, we use that, conditionally on $\mathcal {I}_j(u)=0$ forall $j\in [i]\setminus \{1\}$, the equality

$$\begin{aligned} \mathcal {S}_{t}^{\scriptscriptstyle (1)}=\tfrac{\lambda +\zeta }{ab}t+\sum _{j\ge i+1} c_j(\mathcal {I}_j(t)-c_jt) +c_1-\sum _{j=1}^i c_j^2t \end{aligned}$$

holds.

The event $\big \{\mathcal {I}_j(u)=0\forall j\in [i]\setminus \{1\}\big \}$ is decreasing (recall the notions used in the proof of Lemma 2.12) in the random variables $(T_i)_{i\ge 2}$, while the event $\{\mathcal {S}_{[0,u]}^{\scriptscriptstyle (1)}>0\}$ is increasing. Thus, by the FKG-inequality,

2.81 $$\begin{aligned} \mathbb {P}\big (\mathcal {S}_{[0,u]}^{\scriptscriptstyle (1)}>0\mid \mathcal {I}_j(u)=0 \, \forall j\in [i]\setminus \{1\}\big ) \le \mathbb {P}(\mathcal {S}_{[0,u]}^{\scriptscriptstyle (1)}>0)=\mathbb {P}(H_1(0)>u). \end{aligned}$$

We can identify

2.82 $$\begin{aligned} \mathbb {P}(\mathcal {I}_j(u)=0\forall j\in [i-1]) ={\mathrm {e}}^{-\sum _{j=1}^{i-1} c_j u}. \end{aligned}$$

Combining (2.77), (2.81)–(2.82) we arrive at

2.83 $$\begin{aligned} \mathbb {P}(\gamma _1(\lambda )>au) \le \mathbb {P}(H_1(0)>u)\Big [1+\sum _{i\ge 2}{\mathrm {e}}^{-\sum _{j=1}^{i-1} c_j u}\Big ]. \end{aligned}$$

Since $c_j=j^{-\alpha }$ with $\alpha \in (1/3,1/2)$, $\sum _{j=1}^{i-1} c_j\ge (i-1) c_{i-1}=(i-1)^{1-\alpha }$. Therefore,

2.84 $$\begin{aligned} \sum _{i\ge 2}{\mathrm {e}}^{-\sum _{j=1}^{i-1} c_j u}=o(1). \end{aligned}$$

This completes the proof of the Main Theorem. $\square$

## No Middle Ground: Proof of Proposition 2.13

In this section, we show that the probability to hit zero in the time interval $[\varepsilon ,u-Tu^{-(\tau -2)}]$, where *T* is a constant, becomes negligible as $T\rightarrow \infty$.

The strategy of proof is as follows. We start in Proposition 3.2 by investigating the value of $\mathcal {S}_t$ at some discrete times $(t_k)_{k\ge 1}$ in [0, *u*] and show that with high probability $\mathcal {S}_t$ does not deviate far from its mean. Next, in Proposition 3.3, we show that it is unlikely for the process $(\mathcal {S}_t)_{t\ge 0}$ to make a substantial deviation in the interval $[t_k,t_{k+1}]$ from its value in $t_k$.

We start with a preparatory lemma that will allow us to give bounds on the asymptotic parameters appearing in the upcoming proofs:

### Lemma 3.1

(Asymptotics of parameters). There exists $K\ge 1$ such that

3.1 $$\begin{aligned} \widetilde{\mathbb {P}}\left( \sum _{i=2}^{\infty } c_i^2 \mathcal {I}_i(u)\ge K u^{\tau -3}\right) \le Cu^{-(\tau -1)}, \end{aligned}$$

and, for all $|\lambda | \le \delta u$ with $\delta >0$ sufficiently small, there exists $K>0$ such that

3.2 $$\begin{aligned} \widetilde{\mathbb {P}}\left( \sum _{i=2}^{\infty } c_i [1-\mathcal {I}_i(u/2)]\big ({\mathrm {e}}^{\lambda c_i}-1-\lambda c_i\big ) \ge K\lambda ^2u^{\tau -4}\right) \le C u^{-(\tau -1)}. \end{aligned}$$

### Proof

We use the second moment method. With Lemma 2.9 we compute that

3.3 $$\begin{aligned} \widetilde{\mathbb {E}}\left[ \sum _{i=2}^{\infty } c_i^2 \mathcal {I}_i(u)\right] \le \sum _{i=2}^{\infty } c_i^2 C(\theta ) \big ( 1-{\mathrm {e}}^{-c_i u} \big ). \end{aligned}$$

Split the sum into *i* with $c_i u \le 1$ and $c_i u>1$. For the first, we bound $1-{\mathrm {e}}^{-c_i u}\le O(1) c_iu$, for the latter, we bound $1-{\mathrm {e}}^{-c_i u}\le 1$, to obtain

3.4 $$\begin{aligned} \widetilde{\mathbb {E}}\left[ \sum _{i=2}^{\infty } c_i^2 \mathcal {I}_i(u)\right] \le O(1) \sum _{i:c_iu\le 1} c_i^3u +O(1) \sum _{i:c_iu>1} c_i^2= O(1) u^{\tau -3}(1+o(1)), \end{aligned}$$

the latter by an explicit computation using that $c_i=i^{-1/(\tau -1)}$.

Further, with Corollary 2.11

3.5 $$\begin{aligned}&\widetilde{\mathrm{Var}}\left( \sum _{i=2}^{\infty } c_i^2 \mathcal {I}_i(u)\right) \le \sum _{i=2}^{\infty } c_i^4 (1-\widetilde{\mathbb {P}}(T_i\le u)) \nonumber \\&\quad \le C(\theta ) \sum _{i=2}^{\infty } c_i^4{\mathrm {e}}^{-c_i u} = O(1)u^{\tau -5}(1+o(1)). \end{aligned}$$

The Chebychev inequality now proves (3.1).

For (3.2), we again compute

3.6 $$\begin{aligned}&\widetilde{\mathbb {E}}\left[ \sum _{i=2}^{\infty } c_i [1-\mathcal {I}_i(u/2)]\big ({\mathrm {e}}^{\lambda c_i}-1-\lambda c_i\big )\right] \nonumber \\&\quad \le C(\theta ) \sum _{i=2}^{\infty } c_i {\mathrm {e}}^{-c_iu/2}\left[ {\mathrm {e}}^{\lambda c_i}-1-\lambda c_i\right] \nonumber \\&\quad = C(\theta ) \sum _{i=2}^{\infty } c_i {\mathrm {e}}^{-c_iu/2}{\mathrm {e}}^{|\lambda | c_i}(\lambda c_i)^2/2. \end{aligned}$$

Thus, for $|\lambda |\le \delta u$ and again using Corollary 2.11, we obtain

3.7 $$\begin{aligned} \widetilde{\mathbb {E}}\left[ \sum _{i=2}^{\infty } c_i [1-\mathcal {I}_i(u/2)]\big ({\mathrm {e}}^{\lambda c_i}-1-\lambda c_i\big )\right] =O(\lambda ^2 u^{\tau -4}). \end{aligned}$$

Further,

3.8 $$\begin{aligned}&\widetilde{\mathrm{Var}}\Big (\sum _{i=2}^{\infty } c_i [1-\mathcal {I}_i(u/2)]\big ({\mathrm {e}}^{\lambda c_i}-1-\lambda c_i\big )\Big ) \nonumber \\&\quad \le C(\theta ) \sum _{i=2}^{\infty } c_i^2{\mathrm {e}}^{-c_i u/2}\big ({\mathrm {e}}^{\lambda c_i}-1-\lambda c_i\big )^2 \nonumber \\&\quad \le C(\theta ) |\lambda |^4 \sum _{i=2}^{\infty } c_i^6{\mathrm {e}}^{-c_i u/2}{\mathrm {e}}^{2|\lambda | c_i} =O(|\lambda |^4 u^{\tau -7}). \end{aligned}$$

Again the claim follows from the Chebychev inequality. $\square$

We continue to show that the probability for $\mathcal {S}_t$ to deviate far from its mean at some *discrete* times in the time interval $[\varepsilon ,u-Tu^{-(\tau -2)}]$ is small when *T* is large enough:

### Proposition 3.2

(Probability to deviate far from mean at discrete times). Let $\eta >0$ and $\delta _u=u^{-(\tau -2)}$. For any $\varepsilon >0$ and $M>0$,3.9where we recall the definition of $o_{\scriptscriptstyle T}(1)$ from Proposition 2.13.

### Proof

The proof is split between the cases $t\in [\varepsilon ,u/2]$, $t\in [u/2, u-\varepsilon ]$ and $t\in [u-\varepsilon , u-u^{-(\tau -2)}]$, where $t=k\delta _u$ and $\varepsilon >0$ is some arbitrary constant.

**Proof for** $t\in [\varepsilon ,u/2]$. We start by proving the proposition for $t\in [\varepsilon ,u/2]$, for which we use Proposition 2.7 with $\lambda _1=\pm 1$ and $\lambda _2=0$ to see that, for any $x>0$,

3.10 $$\begin{aligned} \widetilde{\mathbb {P}}\Big ( \big |{\mathcal {S}_{t} - \widetilde{\mathbb {E}}[\mathcal {S}_{t}] \over \sqrt{I_{\scriptscriptstyle V}(t/u) u^{\tau -3}}}\big | >x\Big ) \le c{\mathrm {e}}^{-x}, \end{aligned}$$

where we note that the ${\mathrm {e}}^{\Theta }$ error term can be put inside the constant *c* since $|\Theta |\le o_u(1)+O(t^{3(\tau -3)})$ and $t\ge \varepsilon$ is strictly positive. By (2.32) in Lemma 2.10, $I_{\scriptscriptstyle V}(p) \le c p^{\tau -3}$ for all $p\in [0,1/2]$. Applying this to $p=t/u$ yields

3.11 $$\begin{aligned} \widetilde{\mathbb {P}}\left( |\mathcal {S}_t -\widetilde{\mathbb {E}}[\mathcal {S}_{t}] | > c x t^{(\tau -3)/2} \right) \le c{\mathrm {e}}^{-x}. \end{aligned}$$

By Corollary 2.5(a), we have $\widetilde{\mathbb {E}}[\mathcal {S}_t]/(tu^{\tau -3}) \in [\underline{c},\overline{c}]$ for $t\in [\varepsilon ,u/2]$ and some constants $\underline{c},\overline{c}>0$. Therefore, taking $x=a \eta t^{\frac{1}{2} (5-\tau )}u^{\tau -3}$ for some $a>0$ chosen appropriately,

3.12 $$\begin{aligned} \widetilde{\mathbb {P}}\left( |\mathcal {S}_t- \widetilde{\mathbb {E}}[\mathcal {S}_t]|> \eta \widetilde{\mathbb {E}}[\mathcal {S}_{t}] \right) \le c{\mathrm {e}}^{-a \eta t^{\frac{1}{2}(5-\tau )}u^{\tau -3}}. \end{aligned}$$

We take $t=k\delta _u$ for $k\delta _u \in [\varepsilon ,u/2]$, so that there are at most $u/\delta _u=u^{\tau -1}$possible values of *k*. Thus,

3.13 $$\begin{aligned} \widetilde{\mathbb {P}}\left( \exists t_k\in [\varepsilon ,u/2]:|\mathcal {S}_{t_k}-\widetilde{\mathbb {E}}[\mathcal {S}_{t_k}]| >\eta \widetilde{\mathbb {E}}[\mathcal {S}_{t_k}] \right) \le c(\epsilon )u^{\tau -1}{\mathrm {e}}^{-a \eta u^{(\tau -1)/2}}. \end{aligned}$$

This proves the proposition for $k\delta _u \in [\varepsilon ,u/2]$.

**Proof for** $t\in [u/2,u-\varepsilon ]$. We continue by proving the proposition for $t\in [u/2,u-\varepsilon ]$, for which we again use Proposition 2.7 with $\lambda _1=\pm 1$ and $\lambda _2=0$ to see that, for any $x>0$,

3.14 $$\begin{aligned} \widetilde{\mathbb {P}}\Big ( \big |{\mathcal {S}_{t}- \widetilde{\mathbb {E}}[\mathcal {S}_{t}] \over \sqrt{I_{\scriptscriptstyle V}(t/u) u^{\tau -3}}}\big | >x\Big ) \le c{\mathrm {e}}^{-x}. \end{aligned}$$

By Lemma 2.10 and the fact that $I_{\scriptscriptstyle V}(p)>0$ for every $p\in (0,1)$, we obtain that there exists a constant $c>0$ such that $I_{\scriptscriptstyle V}(p) \ge c$ for all $p\in [1/2, 1-\varepsilon ]$. Applying this to $p=t/u$ yields

3.15 $$\begin{aligned} \widetilde{\mathbb {P}}\left( |\mathcal {S}_t -\widetilde{\mathbb {E}}[\mathcal {S}_{t}] | > c x u^{(\tau -3)/2}\right) \le c{\mathrm {e}}^{-x}. \end{aligned}$$

By Lemma 2.4(d) and Corollary 2.5(b), we have $\widetilde{\mathbb {E}}[\mathcal {S}_t] u^{-(\tau -2)} \in [\underline{c},\overline{c}]$ for all $t\in [u/2,u-\varepsilon ]$ and some constants $\underline{c}=\underline{c}(\varepsilon ),\overline{c}=\overline{c}(\varepsilon )>0$. Therefore, taking $x=a \eta u^{(\tau -1)/2}$ for some $a>0$ chosen appropriately,

3.16 $$\begin{aligned} \widetilde{\mathbb {P}}\left( |\mathcal {S}_t- \widetilde{\mathbb {E}}[\mathcal {S}_t]|> \eta \widetilde{\mathbb {E}}[\mathcal {S}_{t}] \right) \le c{\mathrm {e}}^{-a \eta u^{(\tau -1)/2}}. \end{aligned}$$

We take $t=t_k=k\delta _u$ for $k\delta _u \in [u/2, u-\varepsilon ]$, so that there are at most $u/\delta _u=u^{\tau -1}$ possible values of *k*. Thus,

3.17 $$\begin{aligned} \widetilde{\mathbb {P}}\left( \exists t_k\in [u/2, u-\varepsilon ]:|\mathcal {S}_{t_k}- \widetilde{\mathbb {E}}[\mathcal {S}_{t_k}]|> \eta \widetilde{\mathbb {E}}[\mathcal {S}_{t_k}] \right) \le c(\epsilon )u^{\tau -1}{\mathrm {e}}^{-a \eta u^{(\tau -1)/2}}. \end{aligned}$$

This proves the proposition for $k\delta _u \in [u/2,u-\varepsilon ]$.

**Proof for** $t\in [u-\varepsilon ,u-Tu^{-(\tau -2)}]$: **Rewrite** The proof for $t\in [u-\varepsilon ,u-Tu^{-(\tau -2)}]$ is the hardest, and is split into three steps. We start by rewriting the event of interest. We define $s=u-t$ and investigate $\mathcal {S}_{u-s}$ in what follows, so that now $s\in [Tu^{-(\tau -2)},\varepsilon ]$.

Recall the definition of $Q_u(s)$ in (2.45),

3.18 $$\begin{aligned} Q_u(s)&= u \mathcal {S}_{u-s} - s - (u-s) \mathcal {S}_u =-\sum _{i=2}^{\infty } c_i[u\mathbb {1}_{\{T_i\in (u-s,u]\}}-s\mathcal {I}_i(u)], \end{aligned}$$

so that $|\mathcal {S}_{u-s}-\widetilde{\mathbb {E}}[\mathcal {S}_{u-s}]|>\eta \widetilde{\mathbb {E}}[\mathcal {S}_{u-s}]$ precisely when

3.19 $$\begin{aligned} |Q_u(s) -\widetilde{\mathbb {E}}[Q_u(s)]+(u-s) \big (\mathcal {S}_u-\widetilde{\mathbb {E}}[\mathcal {S}_u]\big )| > \eta u \widetilde{\mathbb {E}}[\mathcal {S}_{u-s}]. \end{aligned}$$

When $\mathcal {S}_u\in [0,M/u]$ and using that $u\widetilde{\mathbb {E}}[\mathcal {S}_u]=o(1)$ by Lemma 2.4(d), we therefore obtain that if (3.19) holds, then

3.20 $$\begin{aligned} |Q_u(s) -\widetilde{\mathbb {E}}[Q_u(s)]| > \eta u \widetilde{\mathbb {E}}[\mathcal {S}_{u-s}]-M+o(1). \end{aligned}$$

By Lemma 2.4(d) and Corollary 2.5(b), we have that $\widetilde{\mathbb {E}}[\mathcal {S}_{u-s}]\ge c su^{\tau -3}$ for some $c>0$. Therefore, $\eta u \widetilde{\mathbb {E}}[\mathcal {S}_{u-s}]\ge c \eta T$, so that, by taking $T=T(M)$ sufficiently large, we obtain that

3.21 $$\begin{aligned}&\widetilde{\mathbb {P}}\Big (\exists s_k\in [Tu^{-(\tau -2)},\varepsilon ]:\big |\mathcal {S}_{u-s_k} - \widetilde{\mathbb {E}} [\mathcal {S}_{u-_k}]\big |>\eta \widetilde{\mathbb {E}} [\mathcal {S}_{u-s_k}], \mathcal {S}_u\in [0,M/u]\Big ) \nonumber \\&\qquad \le \widetilde{\mathbb {P}}\Big (\exists s_k\in [Tu^{-(\tau -2)},\varepsilon ]:|Q_u(s_k) -\widetilde{\mathbb {E}}[Q_u(s_k)]| > \eta c s_ku^{\tau -2}, \mathcal {S}_u\in [0,M/u]\Big ). \end{aligned}$$

We condition on $\mathcal {J}(u)$ from (2.43), and note that $\mathcal {S}_u$ is measurable w.r.t $\mathcal {J}(u)$ to obtain

3.22 $$\begin{aligned}&\widetilde{\mathbb {P}}\Big (\exists s_k\in [Tu^{-(\tau -2)},\varepsilon ]:|Q_u(s_k) -\widetilde{\mathbb {E}}[Q_u(s_k)]|> \eta c s_ku^{\tau -2}, \mathcal {S}_u\in [0,M/u]\Big ) \nonumber \\&\qquad =\widetilde{\mathbb {E}}\Big [\mathbb {1}_{\{\mathcal {S}_u\in [0,M/u]\}} \widetilde{\mathbb {P}}\big (\exists s_k\in [Tu^{-(\tau -2)},\varepsilon ]:|Q_u(s_k) -\widetilde{\mathbb {E}}[Q_u(s_k)]| > \eta c s_ku^{\tau -2}\mid \mathcal {J}(u)\big )\Big ]. \end{aligned}$$

This is the starting point of our analysis. We split, writing $\eta '=\eta /2$,

3.23 $$\begin{aligned}&\widetilde{\mathbb {P}}\big (\exists s_k\in [Tu^{-(\tau -2)},\varepsilon ]:|Q_u(s_k) -\widetilde{\mathbb {E}}[Q_u(s_k)]|> \eta c s_ku^{\tau -2}\mid \mathcal {J}(u)\big ) \nonumber \\&\qquad \le \widetilde{\mathbb {P}}\big (\exists s_k\in [Tu^{-(\tau -2)},\varepsilon ]:|Q_u(s_k) -\widetilde{\mathbb {E}}[Q_u(s_k)\mid \mathcal {J}(u)]|> \eta ' c s_ku^{\tau -2}\mid \mathcal {J}(u)\big )\nonumber \\&\qquad \qquad + \mathbb {1}_{\{\exists s_k\in [Tu^{-(\tau -2)},\varepsilon ]:|\widetilde{\mathbb {E}}[Q_u(s_k)\mid \mathcal {J}(u)]-\widetilde{\mathbb {E}}[Q_u(s_k)]|>\eta ' c s_ku^{\tau -2}\}}. \end{aligned}$$

We conclude using the union bound that

3.24 $$\begin{aligned}&\widetilde{\mathbb {P}}\Big (\exists s_k\in [Tu^{-(\tau -2)},\varepsilon ]:|Q_u(s_k) -\widetilde{\mathbb {E}}[Q_u(s_k)]|> \eta c s_ku^{\tau -2}, \mathcal {S}_u\in [0,M/u]\Big ) \nonumber \\&\qquad \le \sum _{k:s_k\in [Tu^{-(\tau -2)},\varepsilon ]} \widetilde{\mathbb {E}}\Big [\widetilde{\mathbb {P}}\Big (|Q_u(s_k) -\widetilde{\mathbb {E}}[Q_u(s_k)\mid \mathcal {J}(u)]|> \eta ' c s_ku^{\tau -2}\mid \mathcal {J}(u)\Big ) \nonumber \\&\qquad \qquad \mathbb {1}_{\{\mathcal {S}_u\in [0,M/u]\}}\Big ]\nonumber \\&\qquad \qquad + \widetilde{\mathbb {P}}\Big (\exists s_k\in [Tu^{-(\tau -2)},\varepsilon ]:|\widetilde{\mathbb {E}}[Q_u(s_k)\mid \mathcal {J}(u)]-\widetilde{\mathbb {E}}[Q_u(s_k)]|>\eta ' c s_k u^{\tau -2}\Big ). \end{aligned}$$

We will bound both contributions separately, and start by setting the stage. We compute that

3.25 $$\begin{aligned} Q_u(s) -\widetilde{\mathbb {E}}[Q_u(s)\mid \mathcal {J}(u)]&= -\sum _{i=2}^{\infty } c_i u\big [\mathbb {1}_{\{T_i\in (u-s,u]\}}-\widetilde{\mathbb {P}}(T_i\in (u-s,u]\mid \mathcal {J}(u))] \nonumber \\&=-\sum _{i=2}^{\infty } c_i u\big [\mathbb {1}_{\{T_i\in (u-s,u]\}}-p_{i,u}(s)\big ], \end{aligned}$$

where we abbreviate

3.26 $$\begin{aligned} p_{i,u}(s)=\widetilde{\mathbb {P}}(T_i\in (u-s,u]\mid \mathcal {J}(u))=\widetilde{\mathbb {P}}(T_i\in (u-s,u]\mid i\in \mathcal {J}(u)). \end{aligned}$$

It turns out that both contributions in (3.24) can be expressed in terms of $p_{i,u}(s)$, and we continue our analysis by studying this quantity in more detail.

**Proof for** $t\in [u-\varepsilon ,u-Tu^{-(\tau -2)}]$**: Analysis of** $p_{i,u}(s)$. We next analyse the conditional probability $p_{i,u}(s)$. We compute (recall (1.23), (2.29) and (2.43))

3.27 $$\begin{aligned} p_{i,u}(s)= & {} \widetilde{\mathbb {P}}(T_i\in (u-s,u]\mid i\in \mathcal {J}(u)) =\frac{\widetilde{\mathbb {P}}(T_i\in (u-s,u])}{\widetilde{\mathbb {P}}(T_i\le u)} \nonumber \\= & {} \frac{\widetilde{\mathbb {P}}(T_i\le u)-\widetilde{\mathbb {P}}(T_i\le u-s)}{\widetilde{\mathbb {P}}(T_i\le u)}. \end{aligned}$$

Using the distribution of $T_i$ formulated in Lemma 2.9, we obtain, for any $s\in [0,u]$,

3.28 $$\begin{aligned} \widetilde{\mathbb {P}}(T_i\le u-s) = \frac{{\mathrm {e}}^{\theta c_iu}\left( 1-{\mathrm {e}}^{-c_i (u-s)}\right) }{{\mathrm {e}}^{\theta c_iu}\left( 1-{\mathrm {e}}^{-c_iu}\right) +{\mathrm {e}}^{-c_iu}}, \end{aligned}$$

so that

3.29 $$\begin{aligned} p_{i,u}(s)= \frac{{\mathrm {e}}^{\theta c_iu}\left( 1-{\mathrm {e}}^{-c_i u}\right) -{\mathrm {e}}^{\theta c_iu}\left( 1-{\mathrm {e}}^{-c_i (u-s)}\right) }{{\mathrm {e}}^{\theta c_iu}\left( 1-{\mathrm {e}}^{-c_i u}\right) } =\frac{{\mathrm {e}}^{-c_i u}\left( {\mathrm {e}}^{c_i s}-1\right) }{1-{\mathrm {e}}^{-c_i u}}=\frac{{\mathrm {e}}^{c_i s}-1}{{\mathrm {e}}^{c_i u}-1}. \end{aligned}$$

We start by bounding $p_{i,u}(s)$, for $s\in [0,\varepsilon ]$, by

3.30 $$\begin{aligned} p_{i,u}(s)\le O(s/u), \qquad \text {and} \qquad p_{i,u}(s)\le O(c_i s) {\mathrm {e}}^{-c_iu} (c_i u\wedge 1)^{-1}. \end{aligned}$$

Moreover, for *u* sufficiently large,

3.31 $$\begin{aligned} |up_{i,u}(s)-s|\le s(c_iu\wedge 1). \end{aligned}$$

**Proof for** $t\in [u-\varepsilon ,u-Tu^{-(\tau -2)}]$**: Completion first term** (3.24). For the first term in (3.24), we use Markov’s inequality in the form $\mathbb {P}(|X-\mathbb {E}[X]|>a)\le a^{-4} \mathbb {E}[(X-\mathbb {E}[X])^4]$ to obtain

3.32 $$\begin{aligned}&\widetilde{\mathbb {P}}\big (|Q_u(s) -\widetilde{\mathbb {E}}[Q_u(s)\mid \mathcal {J}(u)]| > \eta ' c su^{\tau -2}\mid \mathcal {J}(u)\big ) \le (\eta ' c su^{\tau -2})^{-4} \nonumber \\&\quad \widetilde{\mathbb {E}}\big [(Q_u(s) -\widetilde{\mathbb {E}}[Q_u(s)\mid \mathcal {J}(u)])^4\mid \mathcal {J}(u)\big ], \end{aligned}$$

and recall from (3.25) that

3.33 $$\begin{aligned} Q_u(s) -\widetilde{\mathbb {E}}[Q_u(s)\mid \mathcal {J}(u)]= & {} -\sum _{i=2}^{\infty } c_i u\big [\mathbb {1}_{\{T_i\in (u-s,u]\}}-\widetilde{\mathbb {P}}(T_i\in (u-s,u]\mid \mathcal {J}(u))] \nonumber \\= & {} -\sum _{i=2}^{\infty } c_i u\big [\mathbb {1}_{\{T_i\in (u-s,u]\}}-p_{i,u}(s)\big ]. \end{aligned}$$

The summands are conditionally independent given $\mathcal {J}(u)$ and identically 0 when $\mathcal {I}_i(u)=0$, so that

3.34 $$\begin{aligned}&\widetilde{\mathbb {E}}\big [(Q_u(s) -\widetilde{\mathbb {E}}[Q_u(s)\mid \mathcal {J}(u)])^4\mid \mathcal {J}(u)\big ] \nonumber \\&\quad \le \sum _{i\ge 2} c_i^4 u^4 p_{i,u}(s)\mathcal {I}_i(u) \nonumber \\&\qquad +\sum _{i,j\ge 2:i\ne j} c_i^2 c_j^2 u^4 p_{i,u}(s)(1-p_{i,u}(s))\mathcal {I}_i(u) p_{j,u}(s)(1-p_{j,u}(s))\mathcal {I}_j(u). \end{aligned}$$

By the second bound in (3.30) and Corollary 2.11, the first term is at most

3.35 $$\begin{aligned}&O(1) s u^4 \sum _{i\ge 2} c_i^5 {\mathrm {e}}^{-c_iu} (c_i u\wedge 1)^{-1} \le O(1) s u^4 \nonumber \\&\quad \sum _{i\ge 2} c_i^5 {\mathrm {e}}^{-c_iu} [1+(c_i u)^{-1}]=O(su^{\tau -2}). \end{aligned}$$

By (3.1) in Lemma 3.1, we may assume that $\sum _{i=2}^{\infty } c_i^2 \mathcal {I}_i(u)\le K u^{\tau -3}$, since the complement has a probability that is $o(u^{-(\tau -1)/2})$. Then, in a similar way, using the first bound in (3.30), the second term is at most

3.36 $$\begin{aligned} \Big (\sum _{i\ge 2} c_i^2 u^2 p_{i,u}(s)\mathcal {I}_i(u)\Big )^2&\le O(1)\Big (s\sum _{i\ge 2} c_i^2 u\mathcal {I}_i(u)\Big )^2=O\big ((su^{\tau -2})^2\big ). \end{aligned}$$

As a result,

3.37 $$\begin{aligned} \widetilde{\mathbb {E}}\big [(Q_u(s) -\widetilde{\mathbb {E}}[Q_u(s)\mid \mathcal {J}(u)])^4\mid \mathcal {J}(u)\big ] \le O(su^{\tau -2})+ O\big ((su^{\tau -2})^2\big ). \end{aligned}$$

Since $s\ge Tu^{-(\tau -2)}$, this can be simplified to

3.38 $$\begin{aligned} \widetilde{\mathbb {E}}\big [(Q_u(s) -\widetilde{\mathbb {E}}[Q_u(s)\mid \mathcal {J}(u)])^4\mid \mathcal {J}(u)\big ] \le O\big ((su^{\tau -2})^2\big ). \end{aligned}$$

We conclude using (3.32) that, on the event that $\{\sum _{i=2}^{\infty } c_i^2 \mathcal {I}_i(u)\le K u^{\tau -3}\},$

3.39 $$\begin{aligned}&\widetilde{\mathbb {P}}\big (|Q_u(s) -\widetilde{\mathbb {E}}[Q_u(s)\mid \mathcal {J}(u)]| > \eta ' c su^{\tau -2}\mid \mathcal {J}(u)\big )\le \frac{(cs u^{\tau -2})^2}{(\eta ' c su^{\tau -2})^{4}} \nonumber \\&\quad =O(\eta ^{-4} (su^{\tau -2})^{-2}), \end{aligned}$$

so that, also using that $\widetilde{\mathbb {P}}(\mathcal {S}_u\in [0,M/u]) = O(u^{-(\tau -1)/2})$ by Proposition 2.8,

3.40 $$\begin{aligned}&u^{(\tau -1)/2} \widetilde{\mathbb {E}}\Big [\widetilde{\mathbb {P}}\Big (|Q_u(s_k) -\widetilde{\mathbb {E}}[Q_u(s_k)\mid \mathcal {J}(u)]| > \eta ' c s_ku^{\tau -2}\mid \mathcal {J}(u)\Big )\mathbb {1}_{\{\mathcal {S}_u\in [0,M/u]\}}\Big ]\nonumber \\&\qquad \le O(\eta ^{-4} (su^{\tau -2})^{-2})u^{(\tau -1)/2}\widetilde{\mathbb {P}}(\mathcal {S}_u\in [0,M/u])=O(\eta ^{-4} (su^{\tau -2})^{-2}). \end{aligned}$$

This bound is true for any $s\in [Tu^{-(\tau -2)},\varepsilon ]$. Taking $s=s_k=k u^{-(\tau -2)}$ and summing out over $k\ge T$ leads to

3.41 $$\begin{aligned}&u^{(\tau -1)/2}\sum _{k:s_k\in [Tu^{-(\tau -2)},\varepsilon ]} \nonumber \\&\quad \widetilde{\mathbb {E}}\Big [\widetilde{\mathbb {P}}\Big (|Q_u(s_k) -\widetilde{\mathbb {E}}[Q_u(s_k)\mid \mathcal {J}(u)]| > \eta ' c s_ku^{\tau -2}\mid \mathcal {J}(u)\Big )\mathbb {1}_{\{\mathcal {S}_u\in [0,M/u]\}}\Big ] \nonumber \\&\qquad \le O(\eta ^{-4}) \sum _{k\ge T} k^{-2}=O(\eta ^{-4}/T)=o_{\scriptscriptstyle T}(1), \end{aligned}$$

when we take $T=T(\eta )$ sufficiently large, as required.

**Proof for** $t\in [u-\varepsilon ,u-Tu^{-(\tau -2)}]$**: Completion second term** (3.24). For the second term in (3.24), we need to bound

3.42 $$\begin{aligned} \widetilde{\mathbb {P}}\Big (\exists s_k\in [Tu^{-(\tau -2)}, \varepsilon ]:|\widetilde{\mathbb {E}}[Q_u(s_k)\mid \mathcal {J}(u)]-\widetilde{\mathbb {E}}[Q_u(s_k)]|>\eta ' c s_ku^{\tau -2}\Big ). \end{aligned}$$

We compute using (3.18)

3.43 $$\begin{aligned} \widetilde{\mathbb {E}}[Q_u(s)] =-\sum _{i=2}^{\infty } c_i\big [u\widetilde{\mathbb {P}}(T_i\in (u-s,u])-s\widetilde{\mathbb {P}}(i\in \mathcal {J}(u))\big ], \end{aligned}$$

while

3.44 $$\begin{aligned} \widetilde{\mathbb {E}}[Q_u(s)\mid \mathcal {J}(u)] =-\sum _{i=2}^{\infty } c_i \mathcal {I}_i(u) \big [u\widetilde{\mathbb {P}}(T_i\in (u-s,u]\mid i\in \mathcal {J}(u))-s\big ]. \end{aligned}$$

As a result, using (3.26),

3.45 $$\begin{aligned}&\widetilde{\mathbb {E}}[Q_u(s)\mid \mathcal {J}(u)]-\widetilde{\mathbb {E}}[Q_u(s)] \nonumber \\&\quad =-\sum _{i=2}^{\infty } c_i \big [\mathcal {I}_i(u)-\widetilde{\mathbb {P}}(i\in \mathcal {J}(u))\big ] [up_{i,u}(s)-s]=: s X+Y(s), \end{aligned}$$

where with (3.29)

3.46 $$\begin{aligned}&X=-\sum _{i=2}^{\infty } c_i \big [\mathcal {I}_i(u)-\widetilde{\mathbb {P}}(i\in \mathcal {J}(u))\big ]\frac{1+c_iu-{\mathrm {e}}^{c_iu}}{{\mathrm {e}}^{c_iu}-1}, \nonumber \\&\quad Y(s)=-u\sum _{i=2}^{\infty } c_i \big [\mathcal {I}_i(u)-\widetilde{\mathbb {P}}(i\in \mathcal {J}(u))\big ] \frac{{\mathrm {e}}^{c_is}-1-c_is}{{\mathrm {e}}^{c_iu}-1}. \end{aligned}$$

As a result,

3.47 $$\begin{aligned}&\widetilde{\mathbb {P}}\Big (\exists s_k\in [Tu^{-(\tau -2)},\varepsilon ]:|\widetilde{\mathbb {E}}[Q_u(s_k)\mid \mathcal {J}(u)]-\widetilde{\mathbb {E}}[Q_u(s_k)]|>\eta ' c s_ku^{\tau -2}\Big ) \nonumber \\&\qquad \le \widetilde{\mathbb {P}}\big (|X|\ge \eta ' c u^{\tau -2}/2\big )+ \widetilde{\mathbb {P}}\Big (\exists s_k\in [Tu^{-(\tau -2)},\varepsilon ]:|Y(s_k)|\ge \eta ' c s_ku^{\tau -2}/2\Big ). \end{aligned}$$

For both terms, we use the Chebychev inequality.

For *X*, as $\widetilde{\mathbb {E}}[X]=0$, this leads to

3.48 $$\begin{aligned} \widetilde{\mathbb {P}}\big (|X|\ge \eta ' c u^{\tau -2}/2\big ) \le \frac{4}{(\eta ' c u^{\tau -2})^2}\mathrm{Var}(X). \end{aligned}$$

We use Lemma 2.9 to see that $\widetilde{\mathbb {P}}(i\in \mathcal {J}(u))=\frac{1-{\mathrm {e}}^{-c_i u}}{1-{\mathrm {e}}^{-c_iu} +{\mathrm {e}}^{-c_iu(1+\theta )}}$, so that

3.49 $$\begin{aligned}&\widetilde{\mathbb {P}}(i\in \mathcal {J}(u))\widetilde{\mathbb {P}}(i\not \in \mathcal {J}(u)) =\widetilde{\mathbb {P}}(\mathcal {I}_i(u)=1)\widetilde{\mathbb {P}}(\mathcal {I}_i(u)=0) \nonumber \\&\quad =\frac{\left( 1-{\mathrm {e}}^{-c_i u}\right) {\mathrm {e}}^{-c_iu(1+\theta )}}{\left( 1-{\mathrm {e}}^{-c_iu}+{\mathrm {e}}^{-c_iu(1+\theta )}\right) ^2}\le O(1)c_iu{\mathrm {e}}^{-c_iu(1+\theta )}, \end{aligned}$$

since $1-{\mathrm {e}}^{-x}+{\mathrm {e}}^{-x(1+\theta )}$ is uniformly bounded from below away from 0 for all $x\ge 0$. We use this together with Corollary 2.11 to compute that

3.50 $$\begin{aligned} \mathrm{Var}(X)&=\sum _{i=2}^{\infty } c_i^2 \widetilde{\mathbb {P}}(\mathcal {I}_i(u)=1) \widetilde{\mathbb {P}}(\mathcal {I}_i(u)=0)\Big (\frac{1+c_iu-{\mathrm {e}}^{c_iu}}{{\mathrm {e}}^{c_iu}-1}\Big )^2 \nonumber \\&\le O(1) u\sum _{i=2}^{\infty } c_i^3 {\mathrm {e}}^{-c_iu(1+\theta )}=O(1) u^{\tau -3}. \end{aligned}$$

Therefore,

3.51 $$\begin{aligned}&\widetilde{\mathbb {P}}\big (|X|\ge \eta ' c u^{\tau -2}/2\big ) \le O(1) (\eta ')^{-2} u^{-2(\tau -2)}\mathrm{Var}(X)=O(1) (\eta ')^{-2} u^{-(\tau -1)} \nonumber \\&\quad =o(u^{-(\tau -1)/2}), \end{aligned}$$

as required below.

For the term involving *Y*(*s*), we start by using the union bound to obtain

3.52 $$\begin{aligned}&\widetilde{\mathbb {P}}\Big (\exists s_k\in [Tu^{-(\tau -2)}, \varepsilon ]:|Y(s_k)|\ge \eta ' c s_ku^{\tau -2}/2\Big ) \le \varepsilon u^{\tau -2} \nonumber \\&\quad \max _{k:s_k\in [Tu^{-(\tau -2)}, \varepsilon ]} \widetilde{\mathbb {P}}\big (|Y(s_k)|\ge \eta ' c s_ku^{\tau -2}/2\big ). \end{aligned}$$

Then, by the Chebychev inequality and as $\widetilde{\mathbb {E}}[Y(s_k)]=0$,

3.53 $$\begin{aligned} \widetilde{\mathbb {P}}\big (|Y(s_k)|\ge \eta ' c s_k u^{\tau -2}/2\big )&\le \frac{4}{(\eta ' c s_k u^{\tau -2})^2} \mathrm{Var}(Y(s_k)), \end{aligned}$$

where, using (3.49), ${\mathrm {e}}^{c_is}-1-c_is=O(s^2c_i^2)$ and ${\mathrm {e}}^{c_iu}-1\ge c_iu$,

3.54 $$\begin{aligned}&\mathrm{Var}(Y(s)) =u^2\sum _{i=2}^{\infty } c_i^2 \widetilde{\mathbb {P}}(i\in \mathcal {J}(u))\widetilde{\mathbb {P}}(i\not \in \mathcal {J}(u)) \Big (\frac{{\mathrm {e}}^{c_is}-1-c_is}{{\mathrm {e}}^{c_iu}-1}\Big )^2 \nonumber \\&\quad \le O(s^4) \sum _{i=2}^{\infty } c_i^4{\mathrm {e}}^{-c_iu(1+\theta )}=O(s^4) O(u^{\tau -5}), \end{aligned}$$

where we used Corollary 2.11 in the last equality. Substituting this into (3.52) and (3.53), we arrive at

3.55 $$\begin{aligned}&\widetilde{\mathbb {P}}\Big (\exists s_k\in [Tu^{-(\tau -2)}, \varepsilon ]:|Y(s_k)|\ge \eta ' c s_ku^{\tau -2}/2\Big ) \nonumber \\&\quad \le \varepsilon u^{\tau -2} \max _{k:s_k\in [Tu^{-(\tau -2)}, \varepsilon ]} O(s_k^2 u^{\tau -5} u^{-2(\tau -2)})(\eta ')^{-2}\nonumber \\&\quad =O(\varepsilon ^3/(\eta ')^2) u^{-3}=o(u^{-(\tau -1)/2}), \end{aligned}$$

since $\tau \in (3,4)$. Combining (3.51) and (3.55) in (3.47) completes the proof. $\square$

We now know that with high probability the process does not deviate much from its mean when observed at the discrete times $k\delta _u \in [\varepsilon ,u-T\delta _u]$. We continue to show that this actually holds with high probability on the whole interval $[\varepsilon ,u-T\delta _u]$. We complete the preparations for the proof of Proposition 2.13 by proving that it is unlikely for the process to deviate far from the mean for all times $t\in [\varepsilon , u-T\delta _u]$ simultaneously:

### Proposition 3.3

(Probability to deviate far from mean at some time). For every $\eta >0$ and $M>0$,

3.56 $$\begin{aligned}&\limsup _{u\rightarrow \infty } u^{(\tau -1)/2} \widetilde{\mathbb {P}}\big (\exists \, t\in [\varepsilon ,u-T\delta _u]:|\mathcal {S}_t-\widetilde{\mathbb {E}}[\mathcal {S}_t]| \nonumber \\&\quad \ge 10\eta \widetilde{\mathbb {E}}[\mathcal {S}_t], \mathcal {S}_u\in [0,M/u]\big )=o_{\scriptscriptstyle T}(1). \end{aligned}$$

### Proof

Fix $T>0$ and recall that $\delta _u=u^{-(\tau -2)}$. Let

3.57 $$\begin{aligned} E_u&=\{|\mathcal {S}_{k\delta _u}-\widetilde{\mathbb {E}}[\mathcal {S}_{k\delta _u}]|\le \eta \widetilde{\mathbb {E}}[\mathcal {S}_{k\delta _u}]~\forall k\text { s.t. }k\delta _u\in [\varepsilon ,u-T\delta _u]\} \nonumber \\&\qquad \cap \Big \{\sum _{i=2}^{\infty } c_i [1-\mathcal {I}_i(u/2)]\big ({\mathrm {e}}^{\lambda c_i}-1-\lambda c_i\big ) \le K\lambda ^2u^{\tau -4}\Big \}\cap \Big \{\sum _{i=2}^{\infty } c_i^2 \mathcal {I}_i(u)\le K u^{\tau -3}\Big \}, \end{aligned}$$

where we take $\lambda =\delta u$ with $\delta >0$ sufficiently small and $K \ge 1$ as in Lemma 3.1. We first give a bound on $\widetilde{\mathbb {P}}(E_u^c \cap \{\mathcal {S}_u\in [0,M/u]\})$. We apply (3.1) in Lemma 3.1 to obtain that

3.58 $$\begin{aligned} \widetilde{\mathbb {P}}\left( \sum _{i=2}^{\infty } c_i^2 \mathcal {I}_i(u) \ge K u^{\tau -3}\right) =O(u^{-(\tau -1)})=o(u^{-(\tau -1)/2}), \end{aligned}$$

which is contained in the error term in (3.56). Further, by (3.2) in Lemma 3.1

3.59 $$\begin{aligned}&\widetilde{\mathbb {P}}\left( \sum _{i=2}^{\infty } c_i [1-\mathcal {I}_i(u/2)]\big ({\mathrm {e}}^{\lambda c_i}-1-\lambda c_i\big ) \ge K\lambda ^2u^{\tau -4}\right) \nonumber \\&\quad =O(u^{-(\tau -1)})=o(u^{-(\tau -1)/2}). \end{aligned}$$

Combined with Proposition 3.2, this ensures that

3.60 $$\begin{aligned} \limsup _{u\rightarrow \infty } u^{(\tau -1)/2} \widetilde{\mathbb {P}}\left( E_u^c\cap \{\mathcal {S}_u\in [0,M/u]\} \right) =o_{\scriptscriptstyle T}(1). \end{aligned}$$

As a result, we are left to control the fluctuations of the process on any interval $I_k=[k\delta _u,(k+1)\delta _u]$. We use Boole’s inequality to bound

3.61 $$\begin{aligned}&\widetilde{\mathbb {P}}\left( E_u, \exists \, t\in [\varepsilon ,u-T\delta _u]:|\mathcal {S}_t-\widetilde{\mathbb {E}}[\mathcal {S}_t]|\ge 10\eta \widetilde{\mathbb {E}}[\mathcal {S}_t]\right) \nonumber \\&\quad \le \sum _{k:k\delta _u\in [\varepsilon ,u-T\delta _u]} \widetilde{\mathbb {P}}\big (E_u, \exists \, t\in I_k:|\mathcal {S}_t-\widetilde{\mathbb {E}}[\mathcal {S}_t]|\ge 10\eta \widetilde{\mathbb {E}}[\mathcal {S}_t]\big ). \end{aligned}$$

Let $t_k=k\delta _u$, so that $I_k=[t_{k},t_{k+1}]$. We split the analysis into four cases, depending on whether $t_k\le u/2$ or not, and on whether $\mathcal {S}_t-\widetilde{\mathbb {E}}[\mathcal {S}_t]\ge 10\eta \widetilde{\mathbb {E}}[\mathcal {S}_t]$ or $\mathcal {S}_t-\widetilde{\mathbb {E}}[\mathcal {S}_t]\le -10\eta \widetilde{\mathbb {E}}[\mathcal {S}_t]$, which we refer to as ‘large upper’ and ‘large lower’ deviations, respectively.

In all of the four cases, we take advantage of the following observations concerning the law of our indicator processes under $\widetilde{\mathbb {P}}$. By (1.21),

3.62 $$\begin{aligned} \mathcal {S}_{t_k+t}-\mathcal {S}_{t_k}&= \tilde{\beta }t+\sum _{i=2}^{\infty } c_i [\mathcal {I}_i(t+t_k)-\mathcal {I}_i(t_k)-c_i t] \nonumber \\&= \tilde{\beta }t + \sum _{i=2}^{\infty } c_i [1-\mathcal {I}_i(t_k)] [\mathcal {I}_i(t+t_k)-\mathcal {I}_i(t_k)-c_i t] - t \sum _{i=2}^\infty c_i^2 \mathcal {I}_i(t_k). \end{aligned}$$

For *k* respectively $t_k$ fixed and $t \ge 0$, let $\Delta _i^k(t) = \mathcal {I}_i(t+t_k)-\mathcal {I}_i(t_k) = [1-\mathcal {I}_i(t_k)] [\mathcal {I}_i(t+t_k)-\mathcal {I}_i(t_k) ] \in \{0,1\}$. By (2.30),

3.63 $$\begin{aligned} \widetilde{\mathbb {P}}\left( \Delta _i^k(t)=1\right)&= \widetilde{\mathbb {P}}(\mathcal {I}_i(t+t_k)=1, \mathcal {I}_i(t_k)=0) = \widetilde{\mathbb {P}}(t_k < T_i \le t+t_k) \nonumber \\&= \frac{{\mathrm {e}}^{-c_i t_k}}{1-{\mathrm {e}}^{-c_iu}\left( 1-{\mathrm {e}}^{-\theta c_iu}\right) } \left( 1-{\mathrm {e}}^{-c_i t}\right) , \end{aligned}$$

and

3.64 $$\begin{aligned} \widetilde{\mathbb {P}}(\mathcal {I}_i(t_k)=0) = \frac{{\mathrm {e}}^{-c_i t_k}-{\mathrm {e}}^{-c_i u} \left( 1-{\mathrm {e}}^{-\theta c_i u}\right) }{1-{\mathrm {e}}^{-c_iu}\left( 1-{\mathrm {e}}^{-\theta c_iu}\right) }\le {\mathrm {e}}^{-c_i t_k}. \end{aligned}$$

As a result,

3.65 $$\begin{aligned} \widetilde{\mathbb {P}}\left( \Delta _i^k(t)=1 \mid \mathcal {I}_i(t_k)=0\right) = \frac{{\mathrm {e}}^{-c_i t_k}}{{\mathrm {e}}^{-c_i t_k}-{\mathrm {e}}^{-c_i u} \left( 1-{\mathrm {e}}^{-\theta c_i u}\right) } \left( 1-{\mathrm {e}}^{-c_i t}\right) > 1-{\mathrm {e}}^{-c_i t}.\nonumber \\ \end{aligned}$$

Let $( \mathcal {T}_i ^k)_{i \ge 2}$ be a sequence of independent exponential random variables with mean $1/c_i$ (under $\widetilde{\mathbb {P}}$) that are independent of $\mathcal {F}_{t_k}$, the $\sigma$-algebra generated by $(\mathcal {S}_t)_{t\in [0,t_k]}$. Let $(B_i^k(t))_{0 \le t \le \delta _u}=(B_i^k(t;t_k,u))_{0 \le t \le \delta _u}$ be a sequence of processes that are independent in *i* (and also independent of all randomness so far), non-decreasing, taking values in $\{0,1\}$ and with success probability at time $t \in [0,\delta _u]$ of

3.66 $$\begin{aligned} \widetilde{\mathbb {P}}(B_i^k(t)=1)= & {} \Big ( \frac{{\mathrm {e}}^{-c_i t_k}}{{\mathrm {e}}^{-c_i t_k}-{\mathrm {e}}^{-c_i u} (1-{\mathrm {e}}^{-\theta c_i u})} - 1 \Big ) ({\mathrm {e}}^{c_i t}-1) \nonumber \\= & {} \frac{{\mathrm {e}}^{-c_i u} \left( 1-{\mathrm {e}}^{-\theta c_i u}\right) }{{\mathrm {e}}^{-c_i t_k}-{\mathrm {e}}^{-c_i u} \left( 1-{\mathrm {e}}^{-\theta c_i u}\right) } \left( {\mathrm {e}}^{c_i t}-1\right) . \end{aligned}$$

Thus, conditionally on $\mathcal {I}_i(t_k)=0$,

3.67 $$\begin{aligned} (\Delta _i^k(t))_{t\in [0,\delta _u]}{\mathop {=}\limits ^{d}} \big (\mathbb {1}_{\{\mathcal {T}_i^k \le t\}}+(1-\mathbb {1}_{\{\mathcal {T}_i^k \le t\}})B_i^k(t)\big )_{t\in [0,\delta _u]}. \end{aligned}$$

We can therefore without loss of generality assume that under $\widetilde{\mathbb {P}}$, the sequence of processes $(\mathcal {I}_i(t): t \ge 0)_{i \ge 2}$ in (3.62) is constructed inductively as follows. Recall that $t_{k+1}=t_k+\delta _u$. Conditional on $(\mathcal {I}_i(t_k))_{i \ge 2}$, for $0 \le t \le \delta _u$,

3.68 $$\begin{aligned} \mathcal {I}_i(t+t_k) \equiv \mathcal {I}_i(t_k) + [1-\mathcal {I}_i(t_k)] \big ( \mathbb {1}_{\{\mathcal {T}_i^k \le t\}}+(1-\mathbb {1}_{\{\mathcal {T}_i^k \le t\}}) B_i^k(t) \big ). \end{aligned}$$

For lower deviations (see Part 2 and 4 below), we will use as a lower bound in (3.62)

3.69 $$\begin{aligned}{}[1-\mathcal {I}_i(t_k)] [\mathcal {I}_i(t+t_k)-\mathcal {I}_i(t_k)] = [1-\mathcal {I}_i(t_k)] \Delta _i^k(t) \ge [1-\mathcal {I}_i(t_k)] \mathbb {1}_{\{\mathcal {T}_i^k \le t\}}. \end{aligned}$$

For upper deviations (see Part 1 and 3 below), we require an upper bound instead. In a first step, we replace $\Delta _i^k(t)$ by $\mathbb {1}_{\{\mathcal {T}_i^k \le t\}}$ and show that the resulting error is sufficiently small in case $t_k \le u/2$ (see Part 1). Indeed, let

3.70 $$\begin{aligned}&\mathcal {E}_i^k(t) \equiv [1-\mathcal {I}_i(t_k)] \big ( [\mathcal {I}_i(t+t_k)-\mathcal {I}_i(t_k)] - \mathbb {1}_{\{\mathcal {T}_i^k \le t\}} \big ) \nonumber \\&\quad \quad \quad = [1-\mathcal {I}_i(t_k)] \left( 1-\mathbb {1}_{\{\mathcal {T}_i^k \le t\}}\right) B_i^k(t), \end{aligned}$$

and define, for $t\in I_k=[0,\delta _u]$,

3.71 $$\begin{aligned} \mathcal {B}_{t_k,t}^-=t \sum _{i=2}^\infty c_i^2 \mathcal {I}_i(t_k), \qquad \mathcal {B}_{t_k,t}^+=\sum _{i\ge 2} c_i[1-\mathcal {I}_i(t_k)] \left( 1-\mathbb {1}_{\{\mathcal {T}_i^k \le t\}}\right) B_i^k(t). \end{aligned}$$

Then, we obtain that

3.72 $$\begin{aligned} \mathcal {S}_{t_k+t}-\mathcal {S}_{t_k}&= \tilde{\beta }t + \sum _{i=2}^{\infty } c_i [1-\mathcal {I}_i(t_k)] \left( \mathbb {1}_{\{\mathcal {T}_i^k \le t\}}-c_i t\right) - \mathcal {B}_{t_k,t}^-+\mathcal {B}_{t_k,t}^+. \end{aligned}$$

By Lemma 3.1 and for $t\le \delta _u$, the term $\mathcal {B}_{t_k,t}^-$ is, with probability at least $1-Cu^{-(\tau -1)}$ bounded by $\delta _u K u^{\tau -3}=K/u$, which is $o(\widetilde{\mathbb {E}}[\mathcal {S}_{t_k}])$ for $t_k\in [\varepsilon ,u/2]$ respectively $o_{\scriptscriptstyle T}(\widetilde{\mathbb {E}}[\mathcal {S}_{t_k}])$ for $t_k\in [u/2,u-Tu^{-(\tau -2)}]$ by Corollary 2.5(a), (b) and Lemma 2.4(d). Further, in Part 5 below, we will prove that

3.73 $$\begin{aligned} \widetilde{\mathbb {P}}\left( E_u,\exists t_k\in [\varepsilon , u-Tu^{-(\tau -2)}], t\le \delta _u:\mathcal {B}_{t_k,t}^+\ge 7\eta \widetilde{\mathbb {E}}[\mathcal {S}_{t_k}]\right) =o_{\scriptscriptstyle T}(u^{-(\tau -1)/2}). \end{aligned}$$

We first complete the proof subject to (3.73). In Part 5 we will prove (3.73). There, we will rely on the sharp bounds obtained on the middle term in (3.72), which will be obtained in what follows by careful domination arguments in terms of Lévy processes.

**Part 1: The case** $t_k\le u/2$ **and a large upper deviation** We start by bounding the probability that there exists a $t\in I_k=[t_{k},t_{k+1}]$, $\varepsilon \le t_k \le u/2$ such that $\mathcal {S}_t-\widetilde{\mathbb {E}}[\mathcal {S}_t] \ge 10\eta \widetilde{\mathbb {E}}[\mathcal {S}_t]$. Using that $\widetilde{\mathbb {E}}[\mathcal {S}_{t}]=\widetilde{\mathbb {E}}[\mathcal {S}_{t_k}](1+o(1))$ for any $t\in I_k$ by Corollary 2.5(c), we bound

3.74 $$\begin{aligned} \widetilde{\mathbb {P}}\left( E_u, \exists \, t\in I_k:\mathcal {S}_t-\widetilde{\mathbb {E}}[\mathcal {S}_t]\ge 10\eta \widetilde{\mathbb {E}}[\mathcal {S}_t]\right) \le \widetilde{\mathbb {P}}\left( \exists \, t\le \delta _u:\mathcal {S}_{t_k+t}-\mathcal {S}_{t_k}\ge 8\eta \widetilde{\mathbb {E}}[\mathcal {S}_{t_k}]\right) .\nonumber \\ \end{aligned}$$

By (3.72),

3.75 $$\begin{aligned} \mathcal {S}_{t_k+t}-\mathcal {S}_{t_k} \le \tilde{\beta }t+\sum _{i=2}^{\infty } c_i \left[ \mathbb {1}_{\{\mathcal {T}_i^k \le t\}}-c_i t\right] + \mathcal {B}_{t_k,t}^+. \end{aligned}$$

which can be stochastically dominated by the process $\tilde{\beta }t + \mathcal {R}_t + \mathcal {B}_{t_k,t}^+$ with $\mathcal {R}_t \equiv \sum _{i=2}^{\infty } c_i [N_i(t)-c_i t],$ where $(N_i(t))_{t\ge 0}$ is a Poisson process with rate $c_i$. As a result, with (3.73),

3.76 $$\begin{aligned}&\widetilde{\mathbb {P}}\left( E_u, \exists \, t\le \delta _u:\mathcal {S}_{t_k+t}-\mathcal {S}_{t_k}\ge 8\eta \widetilde{\mathbb {E}}[\mathcal {S}_{t_k}], \mathcal {B}_{t_k,t}^+\le 7\eta \widetilde{\mathbb {E}}[\mathcal {S}_{t_k}]\right) \nonumber \\&\quad \le \widetilde{\mathbb {P}}\left( \exists \, t\le \delta _u:\tilde{\beta }t + \mathcal {R}_t\ge \frac{\eta }{2}\widetilde{\mathbb {E}}[\mathcal {S}_{t_k}]\right) . \end{aligned}$$

Since $(\mathcal {R}_t)_{t\ge 0}$ is a finite-variance Lévy process, it is well-concentrated. In more detail, for $\lambda \in {\mathbb {R}}$, we define the exponential martingale

3.77 $$\begin{aligned} \mathcal {M}_t(\lambda )={\mathrm {e}}^{\lambda \mathcal {R}_t-t\phi (\lambda )}, \qquad \text {where} \qquad \phi (\lambda )=\log {\widetilde{\mathbb {E}}\left[ {\mathrm {e}}^{\lambda \mathcal {R}_1}\right] } =\sum _{i=2}^{\infty } c_i\left[ {\mathrm {e}}^{c_i\lambda }-1-c_i\lambda \right] .\nonumber \\ \end{aligned}$$

Then, for every $\lambda \ge 0$, using that $\phi (\lambda )\ge 0$ and by Doob’s inequality,

3.78 $$\begin{aligned} \widetilde{\mathbb {P}}(\exists \, s\le t:\tilde{\beta }s + \mathcal {R}_s\ge x)&\le \widetilde{\mathbb {P}}\left( \exists \, s\le t:\mathcal {M}_s(\lambda )\ge {\mathrm {e}}^{x\lambda -t\phi (\lambda )-t |\tilde{\beta }| \lambda }\right) \nonumber \\&\le {\mathrm {e}}^{-2[x\lambda -t\phi (\lambda )-t |\tilde{\beta }| \lambda ]}\widetilde{\mathbb {E}}\left[ \mathcal {M}_t(\lambda )^2\right] ={\mathrm {e}}^{-[2x\lambda -t\phi (2\lambda )-2t |\tilde{\beta }| \lambda }]. \end{aligned}$$

We apply this inequality to $x=\frac{\eta }{2}\widetilde{\mathbb {E}}[\mathcal {S}_{t_k}]$, $t=\delta _u$ and $\lambda =1$, and Corollary 2.5(a) implies that $\widetilde{\mathbb {E}}[\mathcal {S}_{t_k}]\ge c t_k u^{\tau -3} =ck/u$ for $t_k=k\delta _u\in [\varepsilon ,u/2]$. Therefore (using $t_k=k\delta _u$)

3.79 $$\begin{aligned} \widetilde{\mathbb {P}}\left( E_u, \exists \, t\le \delta _u:\mathcal {S}_{t_k+t}-\mathcal {S}_{t_k}\ge 8\eta \widetilde{\mathbb {E}}[\mathcal {S}_{t_k}], \mathcal {B}_{t_k,t}^+\le 7\eta \widetilde{\mathbb {E}}[\mathcal {S}_{t_k}]\right) \le (1+o(1)){\mathrm {e}}^{-ck\delta _u u^{\tau -3}},\nonumber \\ \end{aligned}$$

which is small even when summed out over *k* as above.

**Part 2: The case** $t_k\le u/2$ **and a large lower deviation** We continue with bounding the probability that there exists a $t\le \delta _u$ and $\varepsilon \le t_k \le u/2$ such that $\mathcal {S}_{t_k+t}-\widetilde{\mathbb {E}}[\mathcal {S}_{t_k+t}]\le -10\eta \widetilde{\mathbb {E}}[\mathcal {S}_{t_k+t}]$, which is slightly more involved. Here we can use that $\mathcal {B}_{t_k,t}^+\ge 0$. Again using that $\widetilde{\mathbb {E}}[\mathcal {S}_{t_k+t}]=\widetilde{\mathbb {E}}[\mathcal {S}_{t_k}](1+o(1))$ for any $t\le \delta _u$ by Corollary 2.5(c), we bound

3.80 $$\begin{aligned}&\widetilde{\mathbb {P}}\left( E_u, \exists t\le \delta _u:\mathcal {S}_{t_k+t}-\widetilde{\mathbb {E}}[\mathcal {S}_{t_k+t}]\le -10\eta \widetilde{\mathbb {E}}[\mathcal {S}_{t_k+t}]\right) \nonumber \\&\quad \le \widetilde{\mathbb {P}}\left( \exists \, t \le \delta _u:\mathcal {S}_{t_k+t}-\mathcal {S}_{t_k}\le -8\eta \widetilde{\mathbb {E}}[\mathcal {S}_{t_k}]\right) . \end{aligned}$$

Further, using (3.62) and (3.69), as well as the realization that $\mathbb {1}_{\{\mathcal{T}_i^k\le t\}}=\mathbb {1}_{\{N_i(t)\ge 1\}}$, we obtain

3.81 $$\begin{aligned}&\mathcal {S}_{t_k+t}-\mathcal {S}_{t_k} \ge \tilde{\beta }t+\sum _{i=2}^{\infty } c_i \nonumber \\&\quad [1-\mathcal {I}_i(t_k)] [N_i(t)-c_i t] -\sum _{i=2}^{\infty } c_i [1-\mathcal {I}_i(t_k)] [N_i(t)-\mathbb {1}_{\{N_i(t)\ge 1\}}] -\mathcal {B}_{t_k,t}^- \nonumber \\&\quad \ge \tilde{\beta }t + \mathcal {R}_t'-\mathcal {D}_t-\mathcal {B}_{t_k,t}^-, \end{aligned}$$

where we set

3.82 $$\begin{aligned} \mathcal {R}_t'&=\sum _{i=2}^{\infty } c_i [1-\mathcal {I}_i(t_k)] [N_i(t)-c_i t] \end{aligned}$$

and

3.83 $$\begin{aligned} \mathcal {D}_t=\sum _{i=2}^{\infty } c_i [1-\mathcal {I}_i(t_k)] [N_i(t)-\mathbb {1}_{\{N_i(t)\ge 1\}}]. \end{aligned}$$

Thus, conditionally on $(\mathcal {I}_i(t_k))_{i\ge 2}$, the process $(\mathcal {R}_t')_{t\ge 0}$ is a Lévy process similar to the Lévy process investigated in Part 1 above and $\mathcal {D}_t$ is the contribution due to *i* for which $N_i(t)\ge 2$. We deal with the two terms one by one (recalling that we have already dealt with $\mathcal {B}_{t_k,t}^-$ above (3.73)), starting with $(\mathcal {R}_t')_{t\ge 0}$. As in the previous part, we show that

3.84 $$\begin{aligned} \widetilde{\mathbb {P}}\left( \exists \, s\le \delta _u:\tilde{\beta }s + \mathcal {R}_s'\le -4\eta \widetilde{\mathbb {E}}[\mathcal {S}_{t_k}]\right) \end{aligned}$$

is small enough even when summed out over *k* such that $t_k\in [\varepsilon ,u/2]$. This again follows by Doob’s inequality and the bound that for any $\lambda \ge 0$, and with $\mathcal {F}_{t_k}$ the $\sigma$-algebra generated by $(\mathcal {S}_t)_{t\in [0,t_k]}$,

3.85 $$\begin{aligned}&\widetilde{\mathbb {P}}\left( \exists \, s\le t:\tilde{\beta }s + \mathcal {R}_s'\le -x \mid \mathcal {F}_{t_k}\right) \nonumber \\&\quad \le \widetilde{\mathbb {P}}\left( \exists \, s\le t:\mathcal {M}_s'(-\lambda )\ge {\mathrm {e}}^{x\lambda -t\phi '(-\lambda )-t |\tilde{\beta }| \lambda }\mid \mathcal {F}_{t_k}\right) \nonumber \\&\quad \le {\mathrm {e}}^{-\left[ 2x\lambda -t\phi '(-\lambda )-t |\tilde{\beta }| \lambda \right] } \widetilde{\mathbb {E}}\left[ \mathcal {M}_t'(-\lambda )^2 \mid \mathcal {F}_{t_k}\right] ={\mathrm {e}}^{-\left[ 2x\lambda -t\phi '(-2\lambda )-2t |\tilde{\beta }| \lambda \right] }, \end{aligned}$$

where

3.86 $$\begin{aligned} \mathcal {M}_t'(\lambda )= {\mathrm {e}}^{\lambda \mathcal {R}_t'-t\phi '(\lambda )}, \qquad \text {with} \qquad \phi '(\lambda )=\log {\widetilde{\mathbb {E}}\left[ {\mathrm {e}}^{\lambda \mathcal {R}'_1}\mid \mathcal {F}_{t_k}\right] }. \end{aligned}$$

We compute that

3.87 $$\begin{aligned} \phi '(\lambda )= & {} \sum _{i=2}^{\infty } \log {\widetilde{\mathbb {E}}[{\mathrm {e}}^{\lambda c_i[1-\mathcal {I}_i(t_k)][N_i(1)-c_i]}\mid \mathcal {F}_{t_k}]} \nonumber \\= & {} \sum _{i=2}^{\infty } c_i[1-\mathcal {I}_i(t_k)]\big ({\mathrm {e}}^{\lambda c_i}-1-\lambda c_i\big ). \end{aligned}$$

Now follow the same steps as in Part 1, using that $0 \le \phi '(-2) \le \text{ const. }$ We continue to bound $\mathcal {D}_t$ by bounding

3.88 $$\begin{aligned} \widetilde{\mathbb {P}}\left( \exists \, t\le \delta _u:\mathcal {D}_t\ge 2\eta \widetilde{\mathbb {E}}[\mathcal {S}_{t_k}]\right) =\widetilde{\mathbb {P}}(\mathcal {D}_{\delta _u}\ge 2\eta \widetilde{\mathbb {E}}[\mathcal {S}_{t_k}]), \end{aligned}$$

since the process $t\mapsto \mathcal {D}_t$ is non-decreasing. By the Markov inequality,

3.89 $$\begin{aligned}&\widetilde{\mathbb {P}}(\mathcal {D}_{t}\ge x)\le x^{-1} \widetilde{\mathbb {E}}[\mathcal {D}_t] \le x^{-1} \sum _{i=2}^{\infty } c_i \widetilde{\mathbb {E}}[N_i(t)-\mathbb {1}_{\{N_i(t)\ge 1\}}] \nonumber \\&\quad \le cx^{-1} \sum _{i=2}^{\infty } c_i (c_i t)^2\le c t^2/x. \end{aligned}$$

Applying this to $x=2\eta \widetilde{\mathbb {E}}[\mathcal {S}_{t_k}]$ with $\widetilde{\mathbb {E}}[\mathcal {S}_{t_k}] \ge c t_k u^{\tau -3}$ and $t=\delta _u=u^{-(\tau -2)}$ yields

3.90 $$\begin{aligned} \widetilde{\mathbb {P}}\left( \exists \, t\le \delta _u:\mathcal {D}_{t}\ge 2\eta \widetilde{\mathbb {E}}[\mathcal {S}_{t_k}]\right) \le c u^{-2(\tau -2)} (\eta t_k)^{-1} u^{-(\tau -3)} =c k^{-1} u^{-(2\tau -5)}. \end{aligned}$$

When summing this out over *k* such that $t_k=k\delta _u\in [\varepsilon ,u/2]$ we obtain a bound $c(\log {u}) u^{-(2\tau -5)}=o(u^{-(\tau -1)/2}),$ since $(2\tau -5)>(\tau -1)/2$ precisely when $\tau >3$. This proves that

3.91 $$\begin{aligned} \sum _{k:k\delta _u\in [\varepsilon ,u/2]} \widetilde{\mathbb {P}}(\mathcal {D}_{\delta _u}\ge 2\eta \widetilde{\mathbb {E}}[\mathcal {S}_{t_k}]) =o(u^{-(\tau -1)/2}), \end{aligned}$$

as required. Collecting terms completes Part 2.

**Part 3: The case** $t_k\ge u/2$ **and a large upper deviation** This proof is more subtle. We fix *k* such that $t_k\in [u/2, u-T\delta _u]$ and condition on $\mathcal {F}_{t_k}$, which is the $\sigma$-field generated by $(\mathcal {S}_t)_{t\le t_k}$ to write (recall (3.57))

3.92 $$\begin{aligned}&\widetilde{\mathbb {P}}\big (E_u, \mathcal {S}_u\in [0,M/u], \exists \, t\in I_k:\mathcal {S}_t-\widetilde{\mathbb {E}}[\mathcal {S}_t]\ge 10\eta \widetilde{\mathbb {E}}[\mathcal {S}_t]\big ) \nonumber \\&\quad \le \widetilde{\mathbb {E}}\Big [\mathbb {1}_{\big \{|\mathcal {S}_{t_k}-\widetilde{\mathbb {E}}[\mathcal {S}_{t_k}]| \le \eta \widetilde{\mathbb {E}}[\mathcal {S}_{t_k}], \sum _{i=2}^{\infty } c_i [1-\mathcal {I}_i(u/2)]({\mathrm {e}}^{\lambda c_i}-1-\lambda c_i) \le K\lambda ^2u^{\tau -4}\big \}}\nonumber \\&\quad \quad \times \widetilde{\mathbb {P}}\big (\exists \, t\le \delta _u:\mathcal {S}_{t_k+t}-\widetilde{\mathbb {E}}[\mathcal {S}_{t_k+t}]\ge 10\eta \widetilde{\mathbb {E}}[\mathcal {S}_{t_k+t}]\mid \mathcal {F}_{t_k}\big )\Big ]. \end{aligned}$$

First observe that on $\{ |S_{t_k}-\widetilde{\mathbb {E}}[\mathcal {S}_{t_k}]| \le \eta \widetilde{\mathbb {E}}[\mathcal {S}_{t_k}]\}$, we have

3.93 $$\begin{aligned}&\widetilde{\mathbb {P}}\big (\exists \, t\le \delta _u:\mathcal {S}_{t_k+t}-\widetilde{\mathbb {E}}[\mathcal {S}_{t_k+t}]\ge 10\eta \widetilde{\mathbb {E}}[\mathcal {S}_{t_k+t}]\mid \mathcal {F}_{t_k}\big ) \nonumber \\&\quad \le \widetilde{\mathbb {P}}\big (\exists \, t\le \delta _u:\mathcal {S}_{t_k+t}-\mathcal {S}_{t_k}\ge 8\eta \widetilde{\mathbb {E}}[\mathcal {S}_{t_k}] \mid \mathcal {F}_{t_k}\big ) \end{aligned}$$

by using that $\widetilde{\mathbb {E}}[\mathcal {S}_{t_k+t}]=\widetilde{\mathbb {E}}[\mathcal {S}_{t_k}](1+o_{\scriptscriptstyle T}(1))$ for any $t\le \delta _u$ by Corollary 2.5(d). Using (3.72) similarly to (3.81), we bound

3.94 $$\begin{aligned} \mathcal {S}_{t_k+t}-\mathcal {S}_{t_k} \le \tilde{\beta }t+\sum _{i=2}^{\infty } c_i [1-\mathcal {I}_i(t_k)] [N_i(t)-c_i t] +\mathcal {B}_{t_k,t}^+=\tilde{\beta }t + \mathcal {R}_t'+\mathcal {B}_{t_k,t}^+, \end{aligned}$$

where we note that $\mathcal {R}_t'$ is as in Part 2, (3.81). Conditionally on $\mathcal {F}_{t_k}$, the process $(\mathcal {R}_t')_{t\ge 0}$ is a Lévy process, and we use

3.95 $$\begin{aligned}&\widetilde{\mathbb {P}}\left( \exists \, s\le t:\tilde{\beta }s + \mathcal {R}_s'\ge x\mid \mathcal {F}_{t_k}\right) \nonumber \\&\quad \le \widetilde{\mathbb {P}}\left( \exists \, s\le t:\mathcal {M}_s'(\lambda )\ge {\mathrm {e}}^{x\lambda -t\phi ''(\lambda )-t |\tilde{\beta }| \lambda }\mid \mathcal {F}_{t_k}\right) \nonumber \\&\quad \le {\mathrm {e}}^{-2\left[ x\lambda -t\phi ''(\lambda )-t|\tilde{\beta }|\lambda \right] } \widetilde{\mathbb {E}}\left[ \mathcal {M}_t'(\lambda )^2\mid \mathcal {F}_{t_k}\right] ={\mathrm {e}}^{-2x\lambda +t\phi ''(2\lambda )+2t|\tilde{\beta }|\lambda }, \end{aligned}$$

where we recall Eqs. (3.86) and (3.87). Since ${\mathrm {e}}^{\lambda c_i}-1-\lambda c_i\ge 0$ for every $\lambda \in {\mathbb {R}}$, and since $1-\mathcal {I}_i(t_k)\le 1-\mathcal {I}_i(u/2)$ for every $t_k\ge u/2$, a.s.

3.96 $$\begin{aligned} \phi '(\lambda ) \le \sum _{i=2}^{\infty } c_i[1-\mathcal {I}_i(u/2)]\big ({\mathrm {e}}^{\lambda c_i}-1-\lambda c_i\big ). \end{aligned}$$

On the event $\{\sum _{i=2}^{\infty } c_i [1-\mathcal {I}_i(u/2)]\big ({\mathrm {e}}^{\lambda c_i}-1-\lambda c_i\big ) \le K\lambda ^2u^{\tau -4}\}$ (recall (3.92)), we have that $\phi '(\lambda )\le K\lambda ^2u^{\tau -4}$, so that we can further bound, choosing $\lambda =\delta u$ and $t=\delta _u=u^{-(\tau -2)}$,

3.97 $$\begin{aligned}&\widetilde{\mathbb {P}}(\exists \, s\le t:\tilde{\beta }s + \mathcal {R}_s'\ge x\mid \mathcal {F}_{t_k}) \le {\mathrm {e}}^{-2x\lambda +tK4\lambda ^2u^{\tau -4}+2t|\tilde{\beta }|\lambda } \nonumber \\&\quad \le {\mathrm {e}}^{-2x\delta u+K4\delta ^2 +2 u^{-(\tau -3)} |\tilde{\beta }| \delta }. \end{aligned}$$

We take $x=\frac{\eta }{2}\widetilde{\mathbb {E}}[\mathcal {S}_{t_k}]$ and note that Corollary 2.5(b) and Lemma 2.4(d) yield that $\widetilde{\mathbb {E}}[\mathcal {S}_{t_k}]\ge c(u-t_k) u^{\tau -3}$ for $t_k\in [u/2,u-T\delta _u]$. Then,

3.98 $$\begin{aligned} \widetilde{\mathbb {P}}(\exists \, s\le t:\tilde{\beta }s + \mathcal {R}_s'\ge x\mid \mathcal {F}_{t_k}) \le c {\mathrm {e}}^{-c\delta (u-t_k) u^{\tau -2}}. \end{aligned}$$

Summing over *k* with $t_k=k \delta _u\in [u/2, u-T\delta _u]$ and $\delta _u=u^{-(\tau -2)}$, using Proposition 2.8 and $\widetilde{\mathbb {E}}[\mathcal {S}_{t_k}]\le c(u-t_k) u^{\tau -3}$ by Corollary 2.5(b) and Lemma 2.4(d) yields as an upper bound (recall also (3.92) and the definition of $E_u$ from (3.57))

3.99 $$\begin{aligned}&\sum _{k:t_k\in [u/2, u-T\delta _u]} \widetilde{\mathbb {P}}\big (E_u, \mathcal {S}_u\in [0,M/u], \exists \, t\le \delta _u:\mathcal {S}_{t_k+t}-\widetilde{\mathbb {E}}[\mathcal {S}_{t_k+t}]\ge 10\eta \widetilde{\mathbb {E}}[\mathcal {S}_{t_k+t}],\nonumber \\&\qquad \mathcal {B}_{t_k,t}^+\le 7\eta \widetilde{\mathbb {E}}[\mathcal {S}_{t_k}]\big ) \nonumber \\&\qquad \le c \sum _{k:t_k\in [u/2, u-T\delta _u]} \widetilde{\mathbb {P}}(|\mathcal {S}_{t_k}-\widetilde{\mathbb {E}}[\mathcal {S}_{t_k}]|\le \eta \widetilde{\mathbb {E}}[\mathcal {S}_{t_k}]) {\mathrm {e}}^{-c\delta (u-k\delta _u) u^{\tau -2}}\nonumber \\&\qquad \le c \sum _{k:t_k\in [u/2, u-T\delta _u]} \eta \widetilde{\mathbb {E}}[\mathcal {S}_{t_k}] \Big (\sup _w \widetilde{f}_{\mathcal {S}_{t_k}}(w)\Big ){\mathrm {e}}^{-c\delta (u-k\delta _u) u^{\tau -2}}\nonumber \\&\qquad \le c u^{-(\tau -3)/2} \sum _{k:t_k\in [u/2, u-T\delta _u]} C(u-k\delta _u) u^{\tau -3} {\mathrm {e}}^{-c\delta (u-k\delta _u) u^{\tau -2}} \nonumber \\&\qquad \le c u^{-(\tau -1)/2} \sum _{k:t_k\in [u/2, u-T\delta _u]} C(u-k\delta _u) u^{\tau -2} {\mathrm {e}}^{-c\delta (u-k\delta _u) u^{\tau -2}}\nonumber \\&\qquad \le cu^{-(\tau -1)/2}{\mathrm {e}}^{-c\delta T}=o_{\scriptscriptstyle T}(1)u^{-(\tau -1)/2}, \end{aligned}$$

as required.

**Part 4: The case** $t_k\ge u/2$ **and a large lower deviation** We again start from (3.81), and note that $\mathcal {B}_{t_k,t}^+\ge 0$ and that the bound on $\mathcal {D}_t$ proved in Part 2 and that on $\mathcal {B}_{t_k,t}^{-}$ proved around (3.73) still apply, now using that by Corollary 2.5(b) and Lemma 2.4(d) $\widetilde{\mathbb {E}}[\mathcal {S}_{t_k}]\ge c(u-t_k) u^{\tau -3}$ for $t_k\in [u/2, u-T\delta _u]$ with $\delta _u=u^{-(\tau -2)}$ below (3.89). The exponential martingale bound for $\mathcal {R}_t'$ performed in Part 3 can easily be adapted to deal with a large lower deviation as well. We omit further details. $\square$

**Part 5: The error term** $\mathcal {B}_{t_k,t}^+$. Recall the definition of $\mathcal {B}_{t_k,t}^+$ in (3.71), and the bound that we need to prove in (3.73). Write

3.100 $$\begin{aligned}&\mathcal {B}_{t_k,t}^+=\sum _{i\ge 2} c_i[1-\mathcal {I}_i(t_k)] \left( 1-\mathbb {1}_{\{\mathcal {T}_i^k \le t\}}\right) B_i^k(t) \nonumber \\&\quad =\sum _{i\ge 2} c_i[1-\mathcal {I}_i(t_k)] B_i^k(t) - \sum _{i\ge 2}c_i[1-\mathcal {I}_i(t_k)]\mathbb {1}_{\{\mathcal {T}_i^k \le t\}} B_i^k(t)\equiv \mathcal {B}_{t_k,t}^{+,1}-\mathcal {B}_{t_k,t}^{+,2}. \end{aligned}$$

We first use the first moment method to obtain the estimate

3.101 $$\begin{aligned} \widetilde{\mathbb {P}}\Big (E_u, \exists t_k\in [\varepsilon , u-T\delta _u], t\in [0,\delta _u]:\mathcal {B}_{t_k,t}^{+,2}>\eta \widetilde{\mathbb {E}}[\mathcal {S}_{t_k+t}]\Big ) =o(u^{-(\tau -1)/2}). \end{aligned}$$

Indeed, note that $\sup _{t\le \delta _u}\mathcal {B}_{t_k,t}^{+,2}=\mathcal {B}_{t_k,\delta _u}^{+,2}$ by the fact that $t\mapsto B_i^k(t)$ is non-decreasing. By the independence of $\mathcal {I}_i(t_k) \in \{0,1\}, \mathcal {T}_i^k$ and $B_i^k(t) \in \{0,1\}$ (cf. below (3.65)),

3.102 $$\begin{aligned} \widetilde{\mathbb {E}}\left[ \mathcal {B}_{t_k,\delta _u}^{+,2} \right] = \sum _{i\ge 2}c_i \widetilde{\mathbb {P}}( \mathcal {I}_i(t_k)=0 ) \widetilde{\mathbb {P}}( \mathcal {T}_i^k \le \delta _u) \widetilde{\mathbb {P}}\left( B_i^k(\delta _u)=1 \right) . \end{aligned}$$

Use (3.64) and (3.66) to bound this from above by

3.103 $$\begin{aligned}&\sum _{i\ge 2}c_i \left( 1-{\mathrm {e}}^{-c_i \delta _u}\right) \frac{{\mathrm {e}}^{-c_i u} \left( 1-{\mathrm {e}}^{-\theta c_i u}\right) }{1-{\mathrm {e}}^{-c_iu}\left( 1-{\mathrm {e}}^{-\theta c_iu}\right) } \left( {\mathrm {e}}^{c_i \delta _u}-1\right) \nonumber \\&\quad \le C(\theta ) \sum _{i\ge 2} c_i^3 \delta _u^2 {\mathrm {e}}^{-c_i u} \le C(\theta ) \delta _u^2 u^{\tau -4}=C(\theta ) u^{-\tau }, \end{aligned}$$

using Corollary 2.11. As a result, using Markov’s inequality,

3.104 $$\begin{aligned}&\widetilde{\mathbb {P}}\Big (E_u, \exists k\delta _u\in [\varepsilon , u-T\delta _u], t\in [0,\delta _u]:\mathcal {B}_{t_k,t}^{+,2}>\eta \widetilde{\mathbb {E}}[\mathcal {S}_{t_k+t}]\Big ) \nonumber \\&\qquad \le C(\theta ) u^{-\tau }\sum _{k:k\delta _u\in [\varepsilon , u-T\delta _u]} \frac{1}{\eta \widetilde{\mathbb {E}}[\mathcal {S}_{t_k+t}]} \nonumber \\&\qquad \le \frac{C(\theta )}{\eta } u^{-\tau } \left( \sum _{k:k\delta _u\in [\varepsilon , u/2]} \frac{1}{t_k u^{\tau -3}} + \sum _{k:k\delta _u\in [u/2, u-T\delta _u]} \frac{1}{(u-t_k) u^{\tau -3}} \right) \nonumber \\&\qquad \le C(\theta ) u^{-(\tau -1)} \sum _{k:k\delta _u\in [\varepsilon \wedge T \delta _u, u/2]} \frac{1}{k} \le C(\theta )u^{-(\tau -1)} \left( \log (u \delta _u^{-1}/2) - \log \left( \varepsilon \delta _u^{-1} \wedge T\right) \right) \nonumber \\&\qquad \le C(\theta ) u^{-(\tau -1)} \log (u) = o(u^{-(\tau -1)/2}), \end{aligned}$$

as required.

We continue with $\mathcal {B}_{t_k,t}^{+,1}$, which we bound as

3.105 $$\begin{aligned} \sup _{t\le \delta _u}\mathcal {B}_{t_k,t}^{+,1}=\mathcal {B}_{t_k,\delta _u}^{+,1}, \end{aligned}$$

again by the fact that $t\mapsto B_i^k(t)$ is non-decreasing. Thus, we can write, using (3.72),

3.106 $$\begin{aligned} \mathcal {B}_{t_k,\delta _u}^{+,1}&=\mathcal {S}_{t_k+\delta _u}-\mathcal {S}_{t_k}-\tilde{\beta }\delta _u -\sum _{i=2}^{\infty } c_i [1-\mathcal {I}_i(t_k)] \left( \mathbb {1}_{\{\mathcal {T}_i^k \le \delta _u\}}-c_i \delta _u\right) +\mathcal {B}_{t_k,\delta _u}^-+\mathcal {B}_{t_k,\delta _u}^{+,2} \nonumber \\&\le \mathcal {S}_{t_{k+1}}-\mathcal {S}_{t_k}-\sum _{i=2}^{\infty } c_i [1-\mathcal {I}_i(t_k)] \left( \mathbb {1}_{\{\mathcal {T}_i^k \le \delta _u\}}-c_i \delta _u\right) +\mathcal {B}_{t_k,\delta _u}^-+\mathcal {B}_{t_k,\delta _u}^{+,2}. \end{aligned}$$

Using that $\widetilde{\mathbb {E}}[\mathcal {S}_{t_{k+1}}]=\widetilde{\mathbb {E}}[\mathcal {S}_{t_k}](1+o(1))$ by Corollary 2.5(c), we can bound this by

3.107 $$\begin{aligned} \mathcal {B}_{t_k,\delta _u}^{+,1}&\le |\mathcal {S}_{t_{k+1}}-\widetilde{\mathbb {E}}[\mathcal {S}_{t_{k+1}}]|+|\mathcal {S}_{t_k}-\widetilde{\mathbb {E}}[\mathcal {S}_{t_k}]|+\eta \widetilde{\mathbb {E}}[\mathcal {S}_{t_k}] \nonumber \\&\qquad +\Big |\sum _{i=2}^{\infty } c_i [1-\mathcal {I}_i(t_k)] (\mathbb {1}_{\{\mathcal {T}_i^k \le \delta _u\}}-c_i \delta _u)\Big |+\mathcal {B}_{t_k,\delta _u}^-+\mathcal {B}_{t_k,\delta _u}^{+,2}. \end{aligned}$$

We write

3.108 $$\begin{aligned}&F_u= \Big \{\exists t_k\in [\varepsilon , u-T\delta _u]:\mathcal {B}_{t_k,\delta _u}^-\le \eta \widetilde{\mathbb {E}}[\mathcal {S}_{t_{k}}], \mathcal {B}_{t_k,t}^{+,2}\le \eta \widetilde{\mathbb {E}}[\mathcal {S}_{t_{k}}], \nonumber \\&\quad \Big |\sum _{i=2}^{\infty } c_i [1-\mathcal {I}_i(t_k)] (\mathbb {1}_{\{\mathcal {T}_i^k \le \delta _u\}}-c_i \delta _u)\Big |\le \eta \widetilde{\mathbb {E}}[\mathcal {S}_{t_{k}}]\Big \}. \end{aligned}$$

By the analysis in Parts 1–4, as well as (3.101), we know that (with a possibly different value for $\eta$ for the last term)

3.109 $$\begin{aligned} \widetilde{\mathbb {P}}\left( E_u\cap F_u^c\right) =o_{\scriptscriptstyle T}(u^{-(\tau -1)/2}). \end{aligned}$$

Indeed, for the bound on $\mathcal {B}_{t_k,\delta _u}^-$, see the argument below (3.72). The last term is bounded in terms of Lévy processes in each of the different parts. We conclude that it suffices to investigate $\mathcal {B}_{t_k,\delta _u}^{+,1}$ on the event $E_u\cap F_u$.

On $E_u$, $|\mathcal {S}_{t_{k+1}}-\widetilde{\mathbb {E}}[\mathcal {S}_{t_{k+1}}]|\le \eta \widetilde{\mathbb {E}}[\mathcal {S}_{t_{k+1}}]\le 2\eta \widetilde{\mathbb {E}}[\mathcal {S}_{t_{k}}]$ and $|\mathcal {S}_{t_k}-\widetilde{\mathbb {E}}[\mathcal {S}_{t_k}]|\le \eta \widetilde{\mathbb {E}}[\mathcal {S}_{t_{k}}]$. On $F_u$, the last three terms in (3.107) are bounded by $\eta \widetilde{\mathbb {E}}[\mathcal {S}_{t_{k}}]$ as well. Thus, we obtain, on $E_u\cap F_u$ with probability at least $1-o_{\scriptscriptstyle T}(u^{-(\tau -1)/2})$,

3.110 $$\begin{aligned} \mathcal {B}_{t_k,\delta _u}^{+,1} \le 7\eta \widetilde{\mathbb {E}}[\mathcal {S}_{t_k}] \forall t_k\in [\varepsilon , u-T\delta _u], \end{aligned}$$

as required. $$\square $$

### Proof of Proposition 2.13

The proof follows by Proposition 3.3. Indeed, choose $\eta =1/12$ and observe that $\widetilde{\mathbb {E}}[\mathcal {S}_t] > 0$ on $[\varepsilon ,u-T\delta _u]$, using that $\widetilde{\mathbb {E}}[\mathcal {S}_t]\ge c t u^{\tau -3}$ for $t\in [\varepsilon ,u/2]$ and $\widetilde{\mathbb {E}}[\mathcal {S}_t]\ge c(u-t)u^{\tau -3}$ for all $t \in [u/2,u-T \delta _u]$ by Corollary 2.5(a),(b) and Lemma 2.4(d). $\square$

## Conditional Expectations Given $u\mathcal {S}_u=v$

A major difficulty in the proof of Proposition 2.14 is the fact that, while the summands in the definition of $Q_u(t)$ in (2.45) are independent, this property is lost due to the fact that we *condition on* $\mathcal {S}_u$. The following lemma allows us to deal with such expectations:

### Lemma 4.1

(Conditional expectations given a continuous random variable). Let $G((\mathcal {S}_s)_{s\ge 0})$ be a functional of the process $(\mathcal {S}_s)_{s\ge 0}$ such that $G((\mathcal {S}_s)_{s\ge 0})\ge 0$ $\widetilde{\mathbb {P}}$-a.s., and $0<\widetilde{\mathbb {E}}[G((\mathcal {S}_s)_{s\ge 0})]<\infty$. Then, for every $w\in {\mathbb {R}}$,

4.1 $$\begin{aligned} \widetilde{\mathbb {E}}\big [G((\mathcal {S}_s)_{s\ge 0})\mid \mathcal {S}_u=w\big ]= \frac{1}{\widetilde{f}_{\mathcal {S}_u}(w)} \int _{-\infty }^{+\infty } {\mathrm {e}}^{-{\mathrm {i}}k w} \widetilde{\mathbb {E}}\big [G((\mathcal {S}_s)_{s\ge 0}){\mathrm {e}}^{{\mathrm {i}}k\mathcal {S}_u}\big ] \frac{dk}{2\pi }, \end{aligned}$$

where ${\mathrm {i}}$ denotes the imaginary unit.

For $G((\mathcal {S}_s)_{s\ge 0})=1$, (4.1) is just the usual Fourier inversion theorem applied to the (continuous) random variable $\mathcal {S}_u$. The expectation $\widetilde{\mathbb {E}}\big [G((\mathcal {S}_s)_{s\ge 0}){\mathrm {e}}^{{\mathrm {i}}k\mathcal {S}_u}]$ *factorizes* when $G((\mathcal {S}_s)_{s\ge 0})$ is of product form in the underlying random variables $(\mathcal {I}_i(s))_{s\ge 0}$. In our applications, $\widetilde{\mathbb {E}}\big [G((\mathcal {S}_s)_{s\ge 0})\mid \mathcal {S}_u=w\big ]$ will be close to constant in *w*. Then, in order to compute its asymptotics, it suffices to check that the computation in the proof of Proposition 2.8 is hardly affected by the presence of $G((\mathcal {S}_s)_{s\ge 0})$.

### Proof

Define the measure $\widetilde{\mathbb {P}}^{\scriptscriptstyle G}$ by

4.2 $$\begin{aligned} \widetilde{\mathbb {P}}^{\scriptscriptstyle G}(E)=\frac{\widetilde{\mathbb {E}}\big [G((\mathcal {S}_s)_{s\ge 0})\mathbb {1}_{E}\big ]}{\widetilde{\mathbb {E}}\big [G((\mathcal {S}_s)_{s\ge 0})\big ]}. \end{aligned}$$

Under the measure $\widetilde{\mathbb {P}}^{\scriptscriptstyle G}$, the random variable $\mathcal {S}_u$ is again continuous, since $0<\widetilde{\mathbb {E}}[G((\mathcal {S}_s)_{s\ge 0})] <\infty$. Let $\widetilde{f}_{\mathcal {S}_u}^{\scriptscriptstyle G}$ denote the density of $\mathcal {S}_u$ under the measure $\widetilde{\mathbb {P}}^{\scriptscriptstyle G}$. Then, we obtain, by the Fourier inversion theorem applied to $\widetilde{\mathbb {P}}^{\scriptscriptstyle G}$, that

4.3 $$\begin{aligned} \widetilde{f}_{\mathcal {S}_u}^{\scriptscriptstyle G}(w)=\int _{-\infty }^{+\infty } {\mathrm {e}}^{-{\mathrm {i}}k w} \widetilde{\mathbb {E}}^{\scriptscriptstyle G}\big [{\mathrm {e}}^{{\mathrm {i}}k\mathcal {S}_u}]\frac{dk}{2\pi }. \end{aligned}$$

Now, by (4.2),

4.4 $$\begin{aligned} \widetilde{f}_{\mathcal {S}_u}^{\scriptscriptstyle G}(w)=\frac{\widetilde{\mathbb {E}}\big [G((\mathcal {S}_s)_{s\ge 0})\mid \mathcal {S}_u=w\big ]}{\widetilde{\mathbb {E}}\big [G((\mathcal {S}_s)_{s\ge 0})\big ]} \widetilde{f}_{\mathcal {S}_u}(w), \end{aligned}$$

while

4.5 $$\begin{aligned} \widetilde{\mathbb {E}}^{\scriptscriptstyle G}\big [{\mathrm {e}}^{{\mathrm {i}}k\mathcal {S}_u}]=\frac{\widetilde{\mathbb {E}}\big [G((\mathcal {S}_s)_{s\ge 0}){\mathrm {e}}^{{\mathrm {i}}k\mathcal {S}_u}]}{\widetilde{\mathbb {E}}\big [G((\mathcal {S}_s)_{s\ge 0})\big ]}. \end{aligned}$$

Therefore, substituting both sides in (4.3) and multiplying through by $\widetilde{\mathbb {E}}\big [G((\mathcal {S}_s)_{s\ge 0})\big ]$ proves the claim. $\square$

Let $\widetilde{\mathbb {P}}_v$ denote $\widetilde{\mathbb {P}}$ conditionally on $u\mathcal {S}_u=v$, so that Lemma 4.1 implies that

4.6 $$\begin{aligned} \widetilde{\mathbb {E}}_v\big [G((\mathcal {S}_s)_{s\ge 0})\big ]= \frac{1}{\widetilde{f}_{\mathcal {S}_u}(v/u)} \int _{-\infty }^{+\infty } {\mathrm {e}}^{-{\mathrm {i}}k v/u} \widetilde{\mathbb {E}}\big [G((\mathcal {S}_s)_{s\ge 0}){\mathrm {e}}^{{\mathrm {i}}k\mathcal {S}_u}]\frac{dk}{2\pi }. \end{aligned}$$

In many cases, it shall prove to be convenient to rewrite the above using

4.7 $$\begin{aligned} \widetilde{\mathbb {E}}\big [G((\mathcal {S}_s)_{s\ge 0}){\mathrm {e}}^{{\mathrm {i}}k\mathcal {S}_u}\big ] =\widetilde{\mathbb {E}}\Big [{\mathrm {e}}^{{\mathrm {i}}k\mathcal {S}_u}\widetilde{\mathbb {E}}\big [G((\mathcal {S}_s)_{s\ge 0})\mid \mathcal {J}(u)\big ]\Big ], \end{aligned}$$

since the random variables $(T_i)_{i\in \mathcal {J}(u)}$ are, conditionally on $\mathcal {J}(u)$, independent with

4.8 $$\begin{aligned} \widetilde{\mathbb {P}}(T_i\le u-t \mid T_i\le u) =\frac{1-{\mathrm {e}}^{-c_i(u-t)}}{1-{\mathrm {e}}^{-c_iu}}. \end{aligned}$$

In the following lemma, we investigate the effect on $\mathbb {P}(i\in \mathcal {J}(u))$ of conditioning on $\mathcal {S}_u=w$:

### Lemma 4.2

(The set $\mathcal {J}(u)$ conditionally on $\mathcal {S}_u=w$). There exists a constant $d>0$ such that for any *i* and $w=o(u^{(\tau -3)/2})$,

4.9 $$\begin{aligned} \Big |\widetilde{\mathbb {P}}(j\in \mathcal {J}(u) \mid \mathcal {S}_u=w) -\widetilde{\mathbb {P}}(j\in \mathcal {J}(u))\Big | \le d c_i \widetilde{\mathbb {P}}(j\in \mathcal {J}(u)) \widetilde{\mathbb {P}}(j\not \in \mathcal {J}(u)) u^{-(\tau -3)/2}.\nonumber \\ \end{aligned}$$

### Proof

By Lemma 4.1 (for the second term use $G\equiv 1$)

4.10 $$\begin{aligned}&\left| \widetilde{\mathbb {P}}(j\in \mathcal {J}(u) \mid \mathcal {S}_u=w) -\widetilde{\mathbb {P}}(j\in \mathcal {J}(u)) \right| \nonumber \\&\quad = \frac{1}{\widetilde{f}_{\mathcal {S}_u}(w)} \left| \int _{-\infty }^{+\infty } {\mathrm {e}}^{-{\mathrm {i}}k w} \widetilde{\mathbb {E}}\left[ \left( \mathbb {1}_{\{j\in \mathcal {J}(u)\}} -\widetilde{\mathbb {P}}(j\in \mathcal {J}(u)) \right) {\mathrm {e}}^{{\mathrm {i}}k\mathcal {S}_u} \right] \frac{dk}{2\pi } \right| \nonumber \\&\quad = \frac{1}{u^{(\tau -3)/2} \widetilde{f}_{\mathcal {S}_u}(w)} \nonumber \\&\quad \left| \int _{-\infty }^{+\infty } {\mathrm {e}}^{-{\mathrm {i}}k u^{-(\tau -3)/2} w} \widetilde{\mathbb {E}}\left[ \left( \mathbb {1}_{\{j\in \mathcal {J}(u)\}} -\widetilde{\mathbb {P}}(j\in \mathcal {J}(u)) \right) {\mathrm {e}}^{{\mathrm {i}}k u^{-(\tau -3)/2} \mathcal {S}_u} \right] \frac{dk}{2\pi } \right| . \end{aligned}$$

Recall Lemma 2.9. Under the measure $\widetilde{\mathbb {P}}$, the distribution of the indicator processes $(\mathcal {I}_j(t))_{t\ge 0}$ is that of independent indicator processes. Define $\mathcal {S}_u^{\scriptscriptstyle (j)}=\mathcal {S}_u-c_j(\mathcal {I}_j(u)-c_ju)$. By (1.21) and (2.43), the random variables $\mathcal {I}_j(u)$ and $\mathcal {S}_u^{\scriptscriptstyle (j)}$ are independent under $\widetilde{\mathbb {P}}$. This yields

4.11 $$\begin{aligned}&\left| \widetilde{\mathbb {P}}(j\in \mathcal {J}(u) \mid \mathcal {S}_u=w) -\widetilde{\mathbb {P}}(j\in \mathcal {J}(u)) \right| \nonumber \\&\quad \le \frac{1}{u^{(\tau -3)/2} \widetilde{f}_{\mathcal {S}_u}(w)} \int _{-\infty }^{+\infty } \left| \widetilde{\mathbb {E}}\left[ \left( \mathbb {1}_{\{j\in \mathcal {J}(u)\}} -\widetilde{\mathbb {P}}(j\in \mathcal {J}(u)) \right) {\mathrm {e}}^{{\mathrm {i}}k u^{-(\tau -3)/2} c_j(\mathcal {I}_j(u)-c_ju)} \right] \right| \nonumber \\&\quad \left| \widetilde{\mathbb {E}} \left[ {\mathrm {e}}^{{\mathrm {i}}k u^{-(\tau -3)/2} \mathcal {S}_u^{(j)}} \right] \right| \frac{dk}{2\pi } \nonumber \\&\quad = \frac{1}{u^{(\tau -3)/2} \widetilde{f}_{\mathcal {S}_u}(w)} \int _{-\infty }^{+\infty } \widetilde{\mathbb {P}}(j\not \in \mathcal {J}(u))\widetilde{\mathbb {P}}(j\in \mathcal {J}(u)) \left| {\mathrm {e}}^{{\mathrm {i}}k u^{-(\tau -3)/2} c_j} - 1 \right| \nonumber \\&\quad \left| \widetilde{\mathbb {E}}\left[ {\mathrm {e}}^{{\mathrm {i}}k u^{-(\tau -3)/2} \mathcal {S}_u^{\scriptscriptstyle (j)}} \right] \right| \frac{dk}{2\pi }. \end{aligned}$$

Next we claim that there exist constants $C_1, C_2$ such that for all $j \ge 2$

4.12 $$\begin{aligned} \left| \widetilde{\mathbb {E}}\left[ {\mathrm {e}}^{{\mathrm {i}}k u^{-(\tau -3)/2} \mathcal {S}_u^{\scriptscriptstyle (j)}} \right] \right| \le C_1 {\mathrm {e}}^{-C_2 |k|^{\tau -2}}. \end{aligned}$$

Indeed, for $\mathcal {S}_u^{\scriptscriptstyle (j)}$ replaced by $\mathcal {S}_u$ the result was derived in the proof of Proposition 2.8 in [1]. To prove the same for $\mathcal {S}_u^{\scriptscriptstyle (j)}$ with $j \ge 2$ arbitrary, and following the approach in [1], we obtain for $\frac{k}{2\pi } u^{-(\tau -3)/2-1} \le 1/8$ the bound

4.13 $$\begin{aligned}&\log \left( \left| \widetilde{\mathbb {E}}\left[ {\mathrm {e}}^{{\mathrm {i}}k u^{-(\tau -3)/2} \mathcal {S}_u^{(j)}} \right] \right| \right) \nonumber \\&\quad \le -c u^{4-\tau } k^2 \sum _{i\ge 2: c_i<1/u, i\ne j} c_i^3\le -c_0 |k|^{\tau -2} + c u^{4-\tau } k^2 c_j^3 \mathbb {1}_{\{c_j<1/u\}} \nonumber \\&\quad \le -c_0 |k|^{\tau -2} + c u^{-(\tau -1)} k^2\le -c_0 |k|^{\tau -2} + c, \end{aligned}$$

while for $$y_k=8\frac{k}{2\pi }u^{-(\tau -3)/2}>u$$,

4.14 $$\begin{aligned}&\log \left( \left| \widetilde{\mathbb {E}}\left[ {\mathrm {e}}^{{\mathrm {i}}k u^{-(\tau -3)/2} \mathcal {S}_u^{(j)}} \right] \right| \right) \nonumber \\&\quad \le -c_0 |k|^{\tau -2} + cu^{4-\tau }k^2 c_j^3 \mathbb {1}_{\{c_j<1/y_k\}}\le -c_0 |k|^{\tau -2} + cu^{4-\tau }k^2 y_k^{-3} \nonumber \\&\quad \le -c_0 |k|^{\tau -2} + cu^{4-\tau }u^{(\tau -3)} u^{-1}= -c_0 |k|^{\tau -2} + c. \end{aligned}$$

Substituting (4.12) in (4.11) yields

4.15 $$\begin{aligned}&\left| \widetilde{\mathbb {P}}(j\in \mathcal {J}(u) \mid \mathcal {S}_u=w) -\widetilde{\mathbb {P}}(j\in \mathcal {J}(u)) \right| \nonumber \\&\quad \le \frac{1}{u^{(\tau -3)/2} \widetilde{f}_{\mathcal {S}_u}(w)} \int _{-\infty }^{+\infty } \widetilde{\mathbb {P}}(i\not \in \mathcal {J}(u)) \widetilde{\mathbb {P}}(i\in \mathcal {J}(u)) \left| {\mathrm {e}}^{{\mathrm {i}}k u^{-(\tau -3)/2} c_j} - 1 \right| C_1 {\mathrm {e}}^{-C_2 |k|^{\tau -2}} \frac{dk}{2\pi }. \end{aligned}$$

We further have

4.16 $$\begin{aligned} \left| {\mathrm {e}}^{{\mathrm {i}}k u^{-(\tau -3)/2} c_j} - 1 \right| = \left( 2(1-\cos (k u^{-(\tau -3)/2} c_j)) \right) ^{1/2} \le \sqrt{2} |k| u^{-(\tau -3)/2} c_j, \end{aligned}$$

which yields

4.17 $$\begin{aligned}&\left| \widetilde{\mathbb {P}}(j\in \mathcal {J}(u) \mid \mathcal {S}_u=w) -\widetilde{\mathbb {P}}(j\in \mathcal {J}(u)) \right| \nonumber \\&\quad \le C_3 \frac{1}{u^{(\tau -3)/2} \widetilde{f}_{\mathcal {S}_u}(w)} \int _{-\infty }^{+\infty } \widetilde{\mathbb {P}}(i\not \in \mathcal {J}(u)) \widetilde{\mathbb {P}}(i\in \mathcal {J}(u)) k u^{-(\tau -3)/2} c_j {\mathrm {e}}^{-C_2 |k|^{\tau -2}} \frac{dk}{2\pi } \nonumber \\&\quad = C_3 \widetilde{\mathbb {P}}(i\not \in \mathcal {J}(u))\widetilde{\mathbb {P}}(i\in \mathcal {J}(u)) u^{-(\tau -3)/2} c_j \frac{1}{u^{(\tau -3)/2} \widetilde{f}_{\mathcal {S}_u}(w)} \int _{-\infty }^{+\infty } k {\mathrm {e}}^{-C_2 |k|^{\tau -2}} \frac{dk}{2\pi }. \end{aligned}$$

For $w=o(u^{(\tau -3)/2})$ and by Proposition 2.8, $u^{(\tau -3)/2} \widetilde{f}_{\mathcal {S}_u}(w)=B(1+o(1))$ uniformly in *w* and the claim in (i) follows. $\square$

### Corollary 4.3

There exists a constant $C>0$ such that for any *i* and $w=o(u^{(\tau -3)/2})$,

4.18 $$\begin{aligned} \widetilde{\mathbb {P}}(i\in \mathcal {J}(u) \mid \mathcal {S}_u=w) \le C (1\wedge c_iu). \end{aligned}$$

### Proof

The bound by 1 is obvious. The bound by $Cc_iu$ follows once we recall (2.30) and observe that for $c_j\le 1/u$, $\widetilde{\mathbb {P}}(T_j \le u) = \widetilde{\mathbb {P}}(j\in \mathcal {J}(u)) \le C(\tau ) c_j u$. Now use Lemma 4.2(i). $\square$

## The Near-End Ground: Proof of Proposition 2.14

In this section, we prove Proposition 2.14. The proof is divided into several key parts. In Sect. 5.1, we show convergence of the mean process $A_u$ in Proposition 2.14(a). In Sect. 5.2, we prove the convergence of $B_u$ in Proposition 2.14(b).

### Convergence of the Mean Process $A_u$

Recall the definition of $A_u$ from (2.50). By (4.8),

5.1 $$\begin{aligned} A_u(tu^{-(\tau -2)}) =-\sum _{j\in \mathcal {J}(u)} c_j \left[ u\frac{{\mathrm {e}}^{-c_j(u-tu^{-(\tau -2)})}-{\mathrm {e}}^{-c_ju}}{1-{\mathrm {e}}^{-c_ju}}-tu^{-(\tau -2)} \right] . \end{aligned}$$

We use that $|{\mathrm {e}}^x-1-x| \le {\mathrm {e}}^D x^2/2$ for $0 \le x \le D$ with $x=c_jtu^{-(\tau -2)}$, where for $0 \le t \le T$, $c_jtu^{-(\tau -2)} \le tu^{-(\tau -2)} \le \text{ const. }$, to obtain

5.2 $$\begin{aligned} A_u(tu^{-(\tau -2)}) = -\sum _{j\in \mathcal {J}(u)} c_jtu^{-(\tau -2)} \left[ c_ju\frac{{\mathrm {e}}^{-c_ju}}{1-{\mathrm {e}}^{-c_ju}}-1 \right] +E_u(t) \end{aligned}$$

with an error term $E_u(t)$ bounded by

5.3 $$\begin{aligned} |E_u(t)|&\le C \sum _{j\in \mathcal {J}(u)} \left( c_jtu^{-(\tau -2)} \right) ^2 c_ju \frac{{\mathrm {e}}^{-c_ju}}{1-{\mathrm {e}}^{-c_ju}} \nonumber \\&\le C T^2 u^{-2(\tau -2)} \left[ u\sum _{j\in \mathcal {J}(u):c_j>1/u} c_j^3 + \sum _{j\in \mathcal {J}(u):c_j\le 1/u} c_j^2\right] , \end{aligned}$$

uniformly in $t \le T$. Since $\sum _{j \ge 2} c_j^3 < \infty$ and $u^{-2(\tau -2)+1}=u^{5-2\tau }=o(1)$, the first term vanishes. Further, by Corollary 4.3 with $w=v/u$,

5.4 $$\begin{aligned}&u^{-2(\tau -2)}\widetilde{\mathbb {E}}_v\left[ \sum _{j\in \mathcal {J}(u):c_j\le 1/u} c_j^2\right] =u^{-2(\tau -2)} \sum _{j\in \mathcal {J}(u):c_j\le 1/u} c_j^2\widetilde{\mathbb {P}}_v(j\in \mathcal {J}(u)) \le u^{5-2\tau } \nonumber \\&\quad \sum _{j\in \mathcal {J}(u):c_j\le 1/u} c_j^3=o(1), \end{aligned}$$

so that also the second term is $o_{\scriptscriptstyle \widetilde{\mathbb {P}}_v}(1)$.

In the above proof, we see that it is useful to split a sum over $j \in \mathcal {J}(u)$ into $j\in \mathcal {J}(u)$ such that $c_j>1/u$ and $j\in \mathcal {J}(u)$ such that $c_j \le 1/u$. Then we use upper bounds similar to the ones in Corollary 4.3 to bound the arising sums. We will follow this strategy often below.

We further rewrite (5.2) into

5.5 $$\begin{aligned}&A_u(tu^{-(\tau -2)}) = t \sum _{j\in \mathcal {J}(u)} q_j(u)+E_u(t) \qquad \text {with} \nonumber \\&\quad q_j(u) \equiv u^{-(\tau -2)}c_j{\mathrm {e}}^{-c_ju}\frac{{\mathrm {e}}^{c_ju}-1-c_ju}{1-{\mathrm {e}}^{-c_ju}}. \end{aligned}$$

Note that $0 \le q_j(u) \le 1$ for *u* big. Below, we will frequently rely on the bounds

5.6 $$\begin{aligned} q_j(u) \le C(\tau ) u^{-(\tau -2)}c_j (1\wedge c_j u) \end{aligned}$$

and, using (2.30) for $t=u$,

5.7 $$\begin{aligned} \widetilde{\mathbb {P}}(T_j \le u) \le C(\tau )(1\wedge c_j u), \qquad 1-\widetilde{\mathbb {P}}(T_j \le u) \le {\mathrm {e}}^{-c_ju(1+\theta )}. \end{aligned}$$

By (5.3), to prove the claim of Proposition 2.14(a), it is enough to show that

5.8 $$\begin{aligned} \kappa _u \equiv \sum _{j\in \mathcal {J}(u)} q_j(u) = \sum _{j\ge 2} \mathcal {I}_j(u) q_j(u) {\mathop {\longrightarrow }\limits ^{\scriptscriptstyle \widetilde{\mathbb {P}}_v}}\kappa . \end{aligned}$$

For this, we compute the Laplace transform of $\kappa _u$ under the measure $\widetilde{\mathbb {P}}_v$ using Lemma 4.1 and a change of variable. For $a\ge 0$,

5.9 $$\begin{aligned} \widetilde{\mathbb {E}}_v [{\mathrm {e}}^{-a \kappa _u}] =\frac{1}{u^{(\tau -3)/2} \widetilde{f}_{\mathcal {S}_u}(v/u)} \int _{-\infty }^{+\infty } {\mathrm {e}}^{-{\mathrm {i}}k vu^{-(\tau -1)/2}} \widetilde{\mathbb {E}}\big [{\mathrm {e}}^{-a \kappa _u}{\mathrm {e}}^{{\mathrm {i}}k u^{-(\tau -3)/2}\mathcal {S}_u}\big ]\frac{dk}{2\pi }. \end{aligned}$$

By Proposition 2.8, for each $v>0$, $u^{(\tau -3)/2} \widetilde{f}_{\mathcal {S}_u}(v/u)\rightarrow B$. We aim to use *dominated convergence* on the integral appearing in (5.9), for which we have to prove (a) pointwise convergence for each $k\in {\mathbb {R}}$; and (b) a uniform bound that is integrable. We start by proving pointwise convergence:

#### Lemma 5.1

(Pointwise convergence). For $a \ge 0$ arbitrary, $v=o(u^{(\tau -1)/2})$, and with $\kappa _u$ as in (5.8),

5.10 $$\begin{aligned} {\mathrm {e}}^{-{\mathrm {i}}k vu^{-(\tau -1)/2}}\widetilde{\mathbb {E}}\big [{\mathrm {e}}^{-a \kappa _u}{\mathrm {e}}^{{\mathrm {i}}k u^{-(\tau -3)/2} \mathcal {S}_u}\big ] = {\mathrm {e}}^{-a \kappa } {\mathrm {e}}^{-I_{\scriptscriptstyle V}(1) k^2/2}+ o(1). \end{aligned}$$

#### Proof

Trivially, ${\mathrm {e}}^{-{\mathrm {i}}k vu^{-(\tau -1)/2}}\rightarrow 1$ pointwise when $v=o(u^{(\tau -1)/2})$. To compute $\widetilde{\mathbb {E}}\big [{\mathrm {e}}^{-a \kappa _u}{\mathrm {e}}^{{\mathrm {i}}k u^{-(\tau -3)/2}\mathcal {S}_u}\big ]$, recall the definition of $\mathcal {S}_u$ from (1.21) and recall that the indicator processes $\mathcal {I}_j(t)=\mathbb {1}_{\{T_j\le t\}}$ are independent under the measure $\widetilde{\mathbb {P}}$ (cf. Lemma 2.9), to see that

5.11 $$\begin{aligned}&\widetilde{\mathbb {E}}\big [{\mathrm {e}}^{-a \kappa _u}{\mathrm {e}}^{{\mathrm {i}}k u^{-(\tau -3)/2} \mathcal {S}_u}\big ] ={\mathrm {e}}^{{\mathrm {i}}k u^{-(\tau -3)/2}+\tilde{\beta }{\mathrm {i}}k u^{-(\tau -5)/2}} \nonumber \\&\quad \times \prod _{j\ge 2} {\mathrm {e}}^{-{\mathrm {i}}k u^{-(\tau -5)/2}c_j^2}\Big (1+\big ({\mathrm {e}}^{-a q_j(u)+{\mathrm {i}}k u^{-(\tau -3)/2} c_j}-1\big ) \widetilde{\mathbb {P}}(T_j \le u) \Big ). \end{aligned}$$

The remainder of the proof proceeds in three steps.

**Step 1: Asymptotic factorization** We start by proving that

5.12 $$\begin{aligned} \widetilde{\mathbb {E}}\big [{\mathrm {e}}^{-a \kappa _u}{\mathrm {e}}^{{\mathrm {i}}k u^{-(\tau -3)/2} \mathcal {S}_u}\big ] = {\mathrm {e}}^{-a \widetilde{\mathbb {E}}[\kappa _u]} \widetilde{\mathbb {E}}\big [{\mathrm {e}}^{{\mathrm {i}}k u^{-(\tau -3)/2} \mathcal {S}_u}\big ] + o(1). \end{aligned}$$

To this end, we first use

5.13 $$\begin{aligned}&\Big | \prod _{j \ge 2} a_j - \prod _{j \ge 2} b_j \Big | \le \sum _{j \ge 2} \prod _{j_1<j} |a_{j_1}| |a_j-b_j| |\prod _{j_2>j} |b_{j_2}| \le \sum _{j \ge 2} |a_j-b_j| \nonumber \\&\qquad \text{ if } \sup _j (|a_j| \vee |b_j|) \le 1, \end{aligned}$$

to get (recall that $q_j(u)\ge 0$)

5.14 $$\begin{aligned} \Big | \prod _{j \ge 2} a_j - \prod _{j \ge 2} b_j \Big |&= \Big | \widetilde{\mathbb {E}}\big [{\mathrm {e}}^{-a \kappa _u}{\mathrm {e}}^{{\mathrm {i}}k u^{-(\tau -3)/2} \mathcal {S}_u}\big ] - {\mathrm {e}}^{-a \widetilde{\mathbb {E}}[\kappa _u]} \widetilde{\mathbb {E}}\big [{\mathrm {e}}^{{\mathrm {i}}k u^{-(\tau -3)/2} \mathcal {S}_u}\big ] \Big | \nonumber \\&= \Big | \prod _{j\ge 2} \widetilde{\mathbb {E}}\big [ {\mathrm {e}}^{-a \mathcal {I}_j(u) q_j(u) + {\mathrm {i}}k u^{-(\tau -3)/2} c_j \mathcal {I}_j(u)} \big ] \nonumber \\&\qquad - \prod _{j\ge 2} {\mathrm {e}}^{-a q_j(u)\widetilde{\mathbb {P}}(T_j \le u)} \widetilde{\mathbb {E}}\big [ {\mathrm {e}}^{{\mathrm {i}}k u^{-(\tau -3)/2} c_j \mathcal {I}_j(u)} \big ] \Big | \nonumber \\&\le \sum _{j \ge 2} \Big | \widetilde{\mathbb {E}}\big [ {\mathrm {e}}^{-a \mathcal {I}_j(u) q_j(u) + {\mathrm {i}}k u^{-(\tau -3)/2} c_j \mathcal {I}_j(u)} \big ] \nonumber \\&\qquad - {\mathrm {e}}^{-a q_j(u)\widetilde{\mathbb {P}}(T_j \le u)} \widetilde{\mathbb {E}}\big [ {\mathrm {e}}^{{\mathrm {i}}k u^{-(\tau -3)/2} c_j \mathcal {I}_j(u)} \big ] \Big | \nonumber \\&\equiv \sum _{j \ge 2} \Delta _j(-a q_j(u)), \end{aligned}$$

where we abbreviate $q \equiv -a q_j(u) \le 0$ such that

5.15 $$\begin{aligned} \Delta _j(q)=\left| \widetilde{\mathbb {E}}\big [ {\mathrm {e}}^{\mathcal {I}_j(u)q+ {\mathrm {i}}k u^{-(\tau -3)/2} c_j \mathcal {I}_j(u)} \big ] - {\mathrm {e}}^{q\widetilde{\mathbb {P}}(T_j \le u)} \widetilde{\mathbb {E}}\big [ {\mathrm {e}}^{{\mathrm {i}}k u^{-(\tau -3)/2} c_j \mathcal {I}_j(u)}\big ] \right| . \end{aligned}$$

To bound $\Delta _j(q)$, we write ${\mathrm {e}}^{{\mathrm {i}}k u^{-(\tau -3)/2} c_j}=1+({\mathrm {e}}^{{\mathrm {i}}k u^{-(\tau -3)/2} c_j}-1)$ and use the triangle inequality to bound each summand by

5.16 $$\begin{aligned}&\Delta _j(q) = \left| \left( 1-\widetilde{\mathbb {P}}(T_j \le u)+{\mathrm {e}}^{q} {\mathrm {e}}^{{\mathrm {i}}k u^{-(\tau -3)/2} c_j} \widetilde{\mathbb {P}}(T_j \le u) \right) \right. \nonumber \\&\quad \left. - {\mathrm {e}}^{q\widetilde{\mathbb {P}}(T_j \le u)} \left( 1-\widetilde{\mathbb {P}}(T_j \le u)+{\mathrm {e}}^{{\mathrm {i}}k u^{-(\tau -3)/2} c_j} \widetilde{\mathbb {P}}(T_j \le u) \right) \right| \nonumber \\&\le \left| 1-\widetilde{\mathbb {P}}(T_j \le u) +{\mathrm {e}}^{q} \widetilde{\mathbb {P}}(T_j \le u) - {\mathrm {e}}^{q\widetilde{\mathbb {P}}(T_j \le u)}\right| \nonumber \\&\quad + \left| {\mathrm {e}}^{q} - {\mathrm {e}}^{q\widetilde{\mathbb {P}}(T_j \le u)} \right| \left| {\mathrm {e}}^{{\mathrm {i}}k u^{-(\tau -3)/2} c_j} - 1 \right| \widetilde{\mathbb {P}}(T_j \le u). \end{aligned}$$

We can bound

5.17 $$\begin{aligned} \left| {\mathrm {e}}^{q} - {\mathrm {e}}^{q\widetilde{\mathbb {P}}(T_j \le u)} \right| \le |q|{\mathrm {e}}^{(q\vee 0)} \qquad \text {and} \qquad \left| {\mathrm {e}}^{{\mathrm {i}}k u^{-(\tau -3)/2} c_j} - 1 \right| \le |k| u^{-(\tau -3)/2} c_j, \end{aligned}$$

which gives a bound $|q|{\mathrm {e}}^{(q\vee 0)}|k| u^{-(\tau -3)/2} c_j \widetilde{\mathbb {P}}(T_j \le u)$ on the last line of (5.16).

To bound the first line of (5.16), we use the error bounds $|{\mathrm {e}}^{-x}-1+x| \le |x|^2$ for all $x\ge 0$ to all the exponential functions in it, to obtain

5.18 $$\begin{aligned} \left| 1-\widetilde{\mathbb {P}}(T_j \le u) +{\mathrm {e}}^q \widetilde{\mathbb {P}}(T_j \le u) - {\mathrm {e}}^{q\widetilde{\mathbb {P}}(T_j \le u)}\right| \le C q^2\widetilde{\mathbb {P}}(T_j \le u). \end{aligned}$$

Together, this leads us to

5.19 $$\begin{aligned}&\Delta _j(-a q_j(u)) = \Delta _j(q) \le C |q| {\mathrm {e}}^{(q\vee 0)} \Big (|q|+|k|u^{-(\tau -3)/2} c_j\Big ) \nonumber \\&\quad \le C(a) q_j(u) \Big (q_j(u)+|k|u^{-(\tau -3)/2} c_j\Big ) \equiv \Xi _j. \end{aligned}$$

To prove (5.12), by (5.14) and (5.19) it is enough to show that $\sum _{j \ge 2} \Xi _j= o(1).$ Consider the sum over $c_j> 1/u$ first. By (5.6),

5.20 $$\begin{aligned} \sum _{j\ge 2:c_j> 1/u} \Xi _j&\le C \sum _{j\ge 2:c_j> 1/u} u^{-(\tau -2)} c_j\Big (u^{-(\tau -2)}c_j+c_ju^{-(\tau -3)/2}\Big ) \nonumber \\&\le C \sum _{j\ge 2:c_j> 1/u} u^{-(\tau -2)}u^{-(\tau -3)/2} c_j^2 \le C \sum _{j\ge 2:c_j > 1/u} u^{-3(\tau -3)/2}c_j^3= o(1), \end{aligned}$$

where we have used that $\sum _j c_j^3<\infty$ and $\tau >3$ in the last equality. For $c_j\le 1/u$ and by (5.6), we similarly get

5.21 $$\begin{aligned}&\sum _{j\ge 2:c_j \le 1/u} \Xi _j \le C \sum _{j\ge 2:c_j \le 1/u} u^{-(\tau -3)} c_j^2\Big (u^{-(\tau -3)}c_j^2+c_ju^{-(\tau -3)/2}\Big ) \le C \nonumber \\&\quad \sum _{j\ge 2:c_j \le 1/u} u^{-3(\tau -3)/2} c_j^3=o(1). \end{aligned}$$

This completes the proof that $\sum _{j \ge 2} \Xi _j= o(1)$ and thus of the claim in (5.12). $\square$

**Step 2: The limit of** $\widetilde{\mathbb {E}}[\kappa _u]$. We proceed by showing that $\lim _{u \rightarrow \infty } \widetilde{\mathbb {E}}[\kappa _u] = \kappa$ with $\kappa >0$ as in (2.52). By definition of $\kappa _u$ in (5.8), $q_j(u)$ in (5.5) and $\widetilde{\mathbb {P}}(T_j \le u)$ in (2.30),

5.22 $$\begin{aligned}&\lim _{u \rightarrow \infty } \widetilde{\mathbb {E}}[\kappa _u] = \lim _{u \rightarrow \infty } \sum _{j\ge 2} q_j(u)\widetilde{\mathbb {P}}(T_j \le u) \nonumber \\&\quad = \lim _{u \rightarrow \infty } u^{-(\tau -1)} \sum _{j \ge 2} c_ju{\mathrm {e}}^{-c_ju}\frac{{\mathrm {e}}^{c_ju}-1-c_ju}{1-{\mathrm {e}}^{-c_ju}} \frac{{\mathrm {e}}^{\theta c_ju}\left( 1-{\mathrm {e}}^{-c_ju}\right) }{{\mathrm {e}}^{\theta c_ju}\left( 1-{\mathrm {e}}^{-c_ju}\right) +{\mathrm {e}}^{-c_ju}} \nonumber \\&\quad = \lim _{\Delta \rightarrow 0^+} \Delta \sum _{j \ge 2} x_j^{-\alpha }{\mathrm {e}}^{-x_j^{-\alpha }} \left[ {\mathrm {e}}^{x_j^{-\alpha }}-1-x_j^{-\alpha } \right] \frac{{\mathrm {e}}^{\theta x_j^{-\alpha }}}{{\mathrm {e}}^{\theta x_j^{-\alpha }}\left( 1-{\mathrm {e}}^{-x_j^{-\alpha }}\right) +{\mathrm {e}}^{-x_j^{-\alpha }}} \nonumber \\&\quad = \int _0^{\infty } x^{-\alpha } \frac{{\mathrm {e}}^{\theta x^{-\alpha }}{\mathrm {e}}^{-x^{-\alpha }}}{{\mathrm {e}}^{\theta x^{-\alpha }}(1-{\mathrm {e}}^{-x^{-\alpha }})+{\mathrm {e}}^{-x^{-\alpha }}} \big [{\mathrm {e}}^{x^{-\alpha }}-1-x^{-\alpha } \big ]dx, \end{aligned}$$

with $\Delta =u^{-(\tau -1)}$ and $x_j=j \Delta , \ j \ge 2$. Here we used that the integrand in the last line of (5.22) is continuous and integrable over $(0,\infty )$. Set $-x^{-\alpha }=z$ to get the representation (2.52) for $\kappa$. $\square$

**Step 3: Completion of the proof** By Proposition 2.7, we know that

5.23 $$\begin{aligned} \widetilde{\mathbb {E}}\left[ {\mathrm {e}}^{\mathrm{i}k u^{-(\tau -3)/2}\mathcal {S}_u}\right] \rightarrow {\mathrm {e}}^{-k^2 I_{\scriptscriptstyle V}(1)/2}. \end{aligned}$$

Therefore, Steps 1-2 and (5.23) complete the proof of pointwise convergence in Lemma 5.1. $\square$

To show that the dominated convergence theorem can be applied, it remains to show that the integrand in (5.9) has an integrable dominating function:

#### Lemma 5.2

(Domination by an integrable function).

5.24 $$\begin{aligned} \int _{-\infty }^\infty \sup _{u \ge u_0} \left| \widetilde{\mathbb {E}}\big [{\mathrm {e}}^{-a \kappa _u}{\mathrm {e}}^{{\mathrm {i}}k u^{-(\tau -3)/2}\mathcal {S}_u}\big ] \right| dk < \infty . \end{aligned}$$

#### Proof

By definition of $\mathcal {S}_u$ from (1.21) and the independence in Lemma 2.9,

5.25 $$\begin{aligned}&\left| \widetilde{\mathbb {E}}\Big [ {\mathrm {e}}^{-a \kappa _u} {\mathrm {e}}^{{\mathrm {i}}k u^{-(\tau -3)/2} \mathcal {S}_u} \Big ] \right| ^2 \nonumber \\&\quad \le \prod _{j \ge 2} \left| \widetilde{\mathbb {P}}(T_j \le u) {\mathrm {e}}^{-a q_j(u)} {\mathrm {e}}^{{\mathrm {i}}k u^{-(\tau -3)/2} c_j} + (1-\widetilde{\mathbb {P}}(T_j \le u)) \right| ^2 \nonumber \\&\quad = \prod _{j \ge 2} \left[ 1 + \widetilde{\mathbb {P}}(T_j \le u)^2 ({\mathrm {e}}^{-2a q_j(u)}+1) \right. \nonumber \\&\qquad \quad \left. + 2 \widetilde{\mathbb {P}}(T_j \le u) \cos \left( k u^{-(\tau -3)/2} c_j\right) {\mathrm {e}}^{-a q_j(u)} (1-\widetilde{\mathbb {P}}(T_j \le u)) -2\widetilde{\mathbb {P}}(T_j \le u) \right] . \end{aligned}$$

We can rewrite each factor as

5.26 $$\begin{aligned}&1 -2\widetilde{\mathbb {P}}(T_j \le u) \left\{ \widetilde{\mathbb {P}}(T_j \le u)\left( 1-{\mathrm {e}}^{-2a q_j(u)}\right) /2+ \left( 1-\cos \left( k u^{-(\tau -3)/2} c_j\right) {\mathrm {e}}^{-a q_j(u)}\right) \right. \nonumber \\&\left. (1-\widetilde{\mathbb {P}}(T_j \le u)) \right\} \nonumber \\&\quad \le 1 -2\widetilde{\mathbb {P}}(T_j \le u) \left( 1-\cos \left( k u^{-(\tau -3)/2} c_j\right) {\mathrm {e}}^{-a q_j(u)}\right) (1-\widetilde{\mathbb {P}}(T_j \le u)), \end{aligned}$$

since $q_j(u)\ge 0$. We then use $\log (1+x) \le x$ for $x \ge -1$ to obtain

5.27 $$\begin{aligned}&\log \left( \left| \widetilde{\mathbb {E}}\Big [{\mathrm {e}}^{-a \kappa _u} {\mathrm {e}}^{{\mathrm {i}}k u^{-(\tau -3)/2} \mathcal {S}_u} \Big ] \right| ^2 \right) \nonumber \\&\quad \le \sum _{j \ge 2} 2 \widetilde{\mathbb {P}}(T_j \le u) \big (\cos (k u^{-(\tau -3)/2} c_j) {\mathrm {e}}^{-a q_j(u)} -1\big ) (1-\widetilde{\mathbb {P}}(T_j \le u)). \end{aligned}$$

The latter equals

5.28 $$\begin{aligned} \sum _{j \ge 2} 2 \widetilde{\mathbb {P}}(T_j \le u) (\cos (k u^{-(\tau -3)/2} c_j) -1) (1-\widetilde{\mathbb {P}}(T_j \le u)) + e_j(u), \end{aligned}$$

with an overall error term (using that $\sup _j q_j(u)$ is arbitrarily small for *u* big enough)

5.29 $$\begin{aligned} \sum _{j \ge 2} |e_j(u)| \le C(a) \sum _{j \ge 2} \widetilde{\mathbb {P}}(T_j \le u) q_j(u) (1-\widetilde{\mathbb {P}}(T_j \le u)). \end{aligned}$$

Applying (5.7), we get

5.30 $$\begin{aligned} \sum _{j \ge 2} |e_j(u)| \le C(a) \Big \{ \sum _{j \ge 2: c_j>1/u} u^{-(\tau -2)}c_j {\mathrm {e}}^{-c_ju(1+\theta )} + \sum _{j \ge 2: c_j\le 1/u} c_ju u^{-(\tau -2)}c_j^2 u \Big \}\le C(a), \end{aligned}$$

where we have used the bounds

5.31 $$\begin{aligned} \sum _{i: c_i>1/u} u^{-(\tau -2)}c_j {\mathrm {e}}^{-c_ju(1+\theta )}\le 1, \quad u^{-(\tau -4)} \sum _{i: c_i\le 1/u} c_i^3 \le C(\tau ), \end{aligned}$$

whose proof is straightforward.

Together with (5.27) and (5.28), we obtain

5.32 $$\begin{aligned}&\log \left( \left| \widetilde{\mathbb {E}}\Big [ {\mathrm {e}}^{-a \kappa _u} {\mathrm {e}}^{{\mathrm {i}}k u^{-(\tau -3)/2} \mathcal {S}_u} \Big ] \right| \right) \nonumber \\&\quad \le \sum _{j \ge 2} \widetilde{\mathbb {P}}(T_j \le u) \left( \cos (k u^{-(\tau -3)/2} c_j) -1\right) (1-\widetilde{\mathbb {P}}(T_j \le u)) + C(a). \end{aligned}$$

As all summands are nonpositive we obtain together with (2.30)

5.33 $$\begin{aligned}&\log \left( \left| \widetilde{\mathbb {E}}\Big [ {\mathrm {e}}^{-a \kappa _u} {\mathrm {e}}^{{\mathrm {i}}k u^{-(\tau -3)/2} \mathcal {S}_u} \Big ] \right| \right) \nonumber \\&\quad \le C \sum _{j \ge 2: c_j \le 1/u} c_j u \left( \cos (k u^{-(\tau -3)/2} c_j) -1\right) + C(a). \end{aligned}$$

Following the proof of [1, Proposition 2.5, (6.7)-(6.10)], we obtain

5.34 $$\begin{aligned} \log \left( \left| \widetilde{\mathbb {E}}\Big [{\mathrm {e}}^{-a \kappa _u} {\mathrm {e}}^{{\mathrm {i}}k u^{-(\tau -3)/2} \mathcal {S}_u} \Big ] \right| \right) \le C_1-C_2 |k|^{\tau -2} \end{aligned}$$

and integrability of $| \widetilde{\mathbb {E}}[ {\mathrm {e}}^{-a \kappa _u} {\mathrm {e}}^{{\mathrm {i}}k u^{-(\tau -3)/2} \mathcal {S}_u}]|$ against *k* uniformly in *u* follows. $\square$

**Completion of the proof of Proposition**2.14(a). By the dominated convergence theorem, Lemmas 5.1 and 5.2 complete the proof of Proposition 2.14(a). $\square$

### Convergence of the Process $B_u$

In this section, we investigate the convergence of the $B_u$ process and prove Proposition 2.14(b). Since the limit is a *random process*, this part is more involved than the previous section. We first note that

5.35 $$\begin{aligned} B_u(tu^{-(\tau -2)})=\sum _{i\in \mathcal {J}(u)} c_iu\left[ \mathbb {1}_{\{T_i\in (u-tu^{-(\tau -2)},u]\}}-\widetilde{\mathbb {P}}\left( T_i>u-tu^{-(\tau -2)} \mid T_i\le u\right) \right] ,\nonumber \\ \end{aligned}$$

and the processes $(\mathbb {1}_{\{T_i\in (u-tu^{-(\tau -2)},u]\}})_{t\ge 0}$ are, conditionally on $\mathcal {J}(u)$, independent. Thus, $(B_u(tu^{-(\tau -2)}))_{t\ge 0}$ is, conditionally on $\mathcal {J}(u)$, a sum of (conditionally) independent processes having zero mean. We make crucial use of this observation, as well as the technique in Lemma 4.1, to compute expectations of various functionals of the process $(B_u(tu^{-(\tau -2)}))_{t\ge 0}$.

In order to prove the stated convergence in distribution, we follow the usual path of first proving weak convergence of the one-dimensional marginals, followed by the weak convergence of all finite-dimensional distributions, and complete the proof by showing tightness. We now discuss each of these steps in more detail.

#### Convergence of the One-Dimensional Marginal of $B_u$

We start by computing the one-dimensional marginal of $B_u(tu^{-(\tau -2)})$ (recall (5.35)) and show that it is consistent with the claimed Lévy process limit. We achieve this by computing the Laplace transform

5.36 $$\begin{aligned} \psi _{u,v}(a)=\widetilde{\mathbb {E}}_v\big [{\mathrm {e}}^{-a B_u(tu^{-(\tau -2)})}\big ], \end{aligned}$$

and proving that it converges to the Laplace transform of the claimed Lévy process limit at time *t*. The main result in this section is the following proposition:

##### Proposition 5.3

(One-time marginal of $B_u(tu^{-(\tau -2)})$). There exists a measure $\Pi$ such that, for every $v, a>0$ fixed and as $u\rightarrow \infty$,

5.37 $$\begin{aligned} \psi _{v,u}(a) \rightarrow {\mathrm {e}}^{t\int _0^{\infty } ({\mathrm {e}}^{-a z}-1+a z)\Pi (dz)}, \end{aligned}$$

which is the Laplace transform of a Lévy process $(L_s)_{s\ge 0}$ with non-negative jumps and characteristic measure $\Pi$

5.38 $$\begin{aligned} \Pi (dz) \equiv {\mathrm {e}}^z \frac{{\mathrm {e}}^{-\theta z}}{{\mathrm {e}}^{-\theta z}(1-{\mathrm {e}}^z)+{\mathrm {e}}^z} (\tau -1) (-z)^{-(\tau -1)} dz. \end{aligned}$$

Therefore, the one-dimensional marginals of the process $(B_u(su^{-(\tau -2)}))_{t\ge 0}$ converge to those of $(L_s)_{s\ge 0}$.

The remainder of this section is devoted to the proof of Proposition 5.3. As for $A_u$, we use Lemma 4.1 and a change of variables to rewrite

5.39 $$\begin{aligned}&\psi _{v,u}(a)\equiv \widetilde{\mathbb {E}}_v\Big [{\mathrm {e}}^{-a B_u(tu^{-(\tau -2)})}\Big ] \nonumber \\&=\frac{1}{u^{(\tau -3)/2} \widetilde{f}_{\mathcal {S}_u}(v/u)} \int _{-\infty }^{+\infty } {\mathrm {e}}^{-{\mathrm {i}}k v u^{-(\tau -1)/2}} \widetilde{\mathbb {E}}\left[ {\mathrm {e}}^{-a B_u(tu^{-(\tau -2)})}{\mathrm {e}}^{{\mathrm {i}}k u^{-(\tau -3)/2} \mathcal {S}_u}\right] \frac{dk}{2\pi }\nonumber \\&= \frac{1}{u^{(\tau -3)/2} \widetilde{f}_{\mathcal {S}_u}(v/u)} \int _{-\infty }^{+\infty } {\mathrm {e}}^{-{\mathrm {i}}k v u^{-(\tau -1)/2}} \widetilde{\mathbb {E}}\Big [\psi _{\scriptscriptstyle \mathcal {J}}(a) {\mathrm {e}}^{{\mathrm {i}}k u^{-(\tau -3)/2} \mathcal {S}_u} \Big ] \frac{dk}{2\pi }, \end{aligned}$$

where

5.40 $$\begin{aligned}&\psi _{\scriptscriptstyle \mathcal {J}}(a) \equiv \widetilde{\mathbb {E}}\Big [{\mathrm {e}}^{-a B_u(tu^{-(\tau -2)})}\mid \mathcal {J}(u)\Big ] \nonumber \\&\quad =\prod _{j\in \mathcal {J}(u)} {\mathrm {e}}^{a c_ju \widetilde{\mathbb {P}}(T_j>u-tu^{-(\tau -2)} \mid T_j\le u)} \nonumber \\&\qquad \Big (1+({\mathrm {e}}^{-a c_ju}-1) \widetilde{\mathbb {P}}(T_j>u-tu^{-(\tau -2)} \mid T_j\le u)\Big )\nonumber \\&\quad =\prod _{j\in \mathcal {J}(u)} {\mathrm {e}}^{a c_ju p_{j,t}^u} \Big (1+({\mathrm {e}}^{-a c_ju}-1)p_{j,t}^u\Big ), \end{aligned}$$

and where we abbreviate

5.41 $$\begin{aligned} p_{j,t}^u = \widetilde{\mathbb {P}}\left( T_j>u-tu^{-(\tau -2)} \mid T_j\le u\right) = \frac{{\mathrm {e}}^{c_jtu^{-(\tau -2)}}-1}{1-{\mathrm {e}}^{-c_ju}} {\mathrm {e}}^{-c_ju}, \end{aligned}$$

by (4.8). We again wish to use dominated convergence on the integral in (5.39).

We proceed along the lines of the proof of the convergence of the mean process $A_u$. Basically, in the proof below, we replace $-a \kappa _u$ in (5.9) (recall the definition of $\kappa _u$ and $q_j(u)$ from (5.8) and (5.5)) by $\sum _{j\in \mathcal {J}(u)} r_{j,t}^u$, where we define

5.42 $$\begin{aligned} r_{j,t}^u \equiv \left( {\mathrm {e}}^{-a c_ju}-1+a c_ju\right) p_{j,t}^u = \left( {\mathrm {e}}^{-a c_ju}-1+a c_ju\right) \frac{{\mathrm {e}}^{c_jtu^{-(\tau -2)}}-1}{1-{\mathrm {e}}^{-c_ju}} {\mathrm {e}}^{-c_ju}.\qquad \quad \end{aligned}$$

In what follows, we frequently make use of the bounds

5.43 $$\begin{aligned} p_{j,t}^u \le C t u^{-(\tau -1)} \left( c_j u {\mathrm {e}}^{-c_ju}\wedge 1\right) \le C t u^{-(\tau -1)}, \end{aligned}$$

and

5.44 $$\begin{aligned} r_{j,t}^u \le C(a,T) c_j u^{-(\tau -2)}(1\wedge c_j u). \end{aligned}$$

We again start by proving pointwise convergence:

##### Lemma 5.4

[Pointwise convergence revisited] For $a \ge 0$ arbitrary, $v=o(u^{(\tau -1)/2})$,

5.45 $$\begin{aligned} {\mathrm {e}}^{-{\mathrm {i}}k vu^{-(\tau -1)/2}}\widetilde{\mathbb {E}}\big [\psi _{\scriptscriptstyle \mathcal {J}}(a){\mathrm {e}}^{{\mathrm {i}}k u^{-(\tau -3)/2} \mathcal {S}_u}\big ] = {\mathrm {e}}^{t\int _{-\infty }^0 ({\mathrm {e}}^{a z}-1-a z) \Pi (dz)}{\mathrm {e}}^{-I_{\scriptscriptstyle V}(1) k^2/2}+ o(1). \end{aligned}$$

##### Proof

The first factor on the left-hand side of (5.45) converges to 1. We identify the limit of the expectation in the following steps that mimic the pointwise convergence proof in Lemma 5.1. It will be convenient to split the asymptotic factorization in Step 1 of that proof into two parts, denoted by Steps 1(a) and 1(b). We start by showing that we can simplify $\psi _{\scriptscriptstyle \mathcal {J}}(a)$:

**Step 1(a): Simplification of** $\psi _{\scriptscriptstyle \mathcal {J}}(a)$ As a first step towards the identification of the pointwise limit, we show that we can simplify the expectation in (5.45) as follows:

5.46 $$\begin{aligned} \widetilde{\mathbb {E}}\left[ \left| \psi _{\scriptscriptstyle \mathcal {J}}(a) - {\mathrm {e}}^{\sum _{j\in \mathcal {J}(u)} r_{j,t}^u} \right| \right] = o(1). \end{aligned}$$

To prove (5.46), we denote the difference in (5.46) by

5.47 $$\begin{aligned} E_u(t) = \prod _{j\in \mathcal {J}(u)} {\mathrm {e}}^{a c_ju p_{j,t}^u} \left| \prod _{j\in \mathcal {J}(u)} \Big (1+({\mathrm {e}}^{-a c_ju}-1) p_{j,t}^u \Big ) - \prod _{j\in \mathcal {J}(u)} {\mathrm {e}}^{({\mathrm {e}}^{-a c_ju}-1) p_{j,t}^u} \right| ,\qquad \quad \end{aligned}$$

so that

5.48 $$\begin{aligned} \Big |\widetilde{\mathbb {E}}\left[ \left| \psi _{\scriptscriptstyle \mathcal {J}}(a) - {\mathrm {e}}^{\sum _{j\in \mathcal {J}(u)} r_{j,t}^u} \right| \right] \Big | \le \widetilde{\mathbb {E}}[E_u(t)]. \end{aligned}$$

Using the first line of (5.13) and applying the error bound $|{\mathrm {e}}^x-(1+x)| \le |x|^2$ for $|x| \le 1$ to the differences $|a_j-b_j|$, the error of the approximation can be bounded by

5.49 $$\begin{aligned}&E_u(t) \le C \prod _{j\in \mathcal {J}(u)} {\mathrm {e}}^{a c_ju p_{j,t}^u} \sum _{j\in \mathcal {J}(u)} \prod _{i\in \mathcal {J}(u), i<j} \Big (1+({\mathrm {e}}^{-a c_iu}-1) p_{i,t}^u \Big ) \left[ \left( {\mathrm {e}}^{-a c_ju}-1\right) p_{j,t}^u \right] ^2 \nonumber \\&\quad \prod _{i\in \mathcal {J}(u), i>j} {\mathrm {e}}^{\left( {\mathrm {e}}^{-a c_iu}-1\right) p_{i,t}^u}. \end{aligned}$$

Next use that $1-x \le {\mathrm {e}}^{-x}$ for $x \ge 0$ to obtain as a further bound to the above

5.50 $$\begin{aligned} C \sum _{j\in \mathcal {J}(u)} {\mathrm {e}}^{a c_ju p_{j,t}^u} \prod _{i\in \mathcal {J}(u) \backslash \{j\}} {\mathrm {e}}^{\left( {\mathrm {e}}^{-a c_iu}-1+a c_iu\right) p_{i,t}^u} \left[ \left( {\mathrm {e}}^{-a c_ju}-1\right) p_{j,t}^u \right] ^2. \end{aligned}$$

For $t \le T$ with $T>0$ fixed, we further have by (5.43) that ${\mathrm {e}}^{a c_ju p_{j,t}^u} \le C(a,T)$. Together with ${\mathrm {e}}^{-x}-1+x\ge 0$ for $x \ge 0$, we obtain

5.51 $$\begin{aligned} E_u(t) \le C(a,T) \prod _{i\in \mathcal {J}(u)} {\mathrm {e}}^{\left( {\mathrm {e}}^{-a c_iu}-1+a c_iu\right) p_{i,t}^u} \sum _{j\in \mathcal {J}(u)} \left[ \left( {\mathrm {e}}^{-a c_ju}-1\right) p_{j,t}^u \right] ^2. \end{aligned}$$

The bound ${\mathrm {e}}^{-x}-1+x\le x^2/2, \,\forall x \ge 0$ yields

5.52 $$\begin{aligned} E_u(t) \le C(a,T) {\mathrm {e}}^{ \frac{a^2}{2} \sum _{i\in \mathcal {J}(u)} (c_iu)^2 p_{i,t}^u} \sum _{j\in \mathcal {J}(u)} \left[ \left( {\mathrm {e}}^{-a c_ju}-1\right) p_{j,t}^u \right] ^2. \end{aligned}$$

We first bound the sum in (5.52). With $1-{\mathrm {e}}^{-x} \le x$ for $x \ge 0$ and by (5.43) we obtain

5.53 $$\begin{aligned}&\sum _{j\in \mathcal {J}(u)} \left[ \left( {\mathrm {e}}^{-a c_ju}-1\right) p_{j,t}^u \right] ^2 \nonumber \\&\quad \le \sum _{j\in \mathcal {J}(u)} (a c_ju)^2 \left( p_{j,t}^u \right) ^2 \le C(a,T) u^{-2(\tau -3)-1} \Big \{ C + \sum _{j\in \mathcal {J}(u):c_j\le 1/u} c_j^2 u^{-1} \Big \}.\qquad \quad \end{aligned}$$

This yields as an upper bound for (5.46) (recall (5.48)),

5.54 $$\begin{aligned}&\widetilde{\mathbb {E}}[E_u(t)] \le C(a,T) u^{-2(\tau -3)-1} \Big \{ \widetilde{\mathbb {E}}\left[ {\mathrm {e}}^{ \frac{a^2}{2} \sum _{i\in \mathcal {J}(u)} (c_iu)^2 p_{i,t}^u} \right] \nonumber \\&\qquad + \sum _{j :c_j\le 1/u} c_j^2 u^{-1} \widetilde{\mathbb {E}}\Big [\mathbb {1}_{\{j \in \mathcal {J}(u)\}} {\mathrm {e}}^{ \frac{a^2}{2} (c_ju)^2 p_{j,t}^u } \Big ] \widetilde{\mathbb {E}}\Big [ {\mathrm {e}}^{ \frac{a^2}{2} \sum _{i\in \mathcal {J}(u) \backslash \{j\}} (c_iu)^2 p_{i,t}^u} \Big ] \Big \} \nonumber \\&\le C(a,T) u^{-2(\tau -3)-1} \left\{ 1 + \sum _{j :c_j\le 1/u} c_j^3 \right\} \widetilde{\mathbb {E}}\left[ {\mathrm {e}}^{ \frac{a^2}{2} \sum _{i\in \mathcal {J}(u)} (c_iu)^2 p_{i,t}^u} \right] , \end{aligned}$$

where we have used (5.7) in the last line.

The claim (5.46) follows once we show that $\widetilde{\mathbb {E}}\Big [ {\mathrm {e}}^{ \frac{a^2}{2} \sum _{i\in \mathcal {J}(u)} (c_iu)^2 p_{i,t}^u}\Big ]$ is bounded. To prove this, consider first the sum over $c_i>1/u$ only. By (5.43) and (5.31),

5.55 $$\begin{aligned}&\sum _{i\in \mathcal {J}(u): c_i>1/u} (c_iu)^2 p_{i,t}^u \le C \sum _{i\in \mathcal {J}(u): c_i>1/u} (c_iu)^2 c_i tu^{-(\tau -2)} {\mathrm {e}}^{-c_iu} \le C \nonumber \\&\quad \sum _{i: c_i>1/u} tu^{-(\tau -1)} \le C(T). \end{aligned}$$

Using (5.43) once more, it remains to show the boundedness of

5.56 $$\begin{aligned}&\widetilde{\mathbb {E}}\left[ {\mathrm {e}}^{ \frac{a^2}{2} \sum _{i\in \mathcal {J}(u): c_i\le 1/u} (c_iu)^2 p_{i,t}^u} \right] \nonumber \\&\quad \le \widetilde{\mathbb {E}}\left[ {\mathrm {e}}^{ C(a) \sum _{i\in \mathcal {J}(u): c_i\le 1/u} (c_iu)^2 t u^{-(\tau -1)} } \right] \nonumber \\&\quad = \prod _{c_i\le 1/u} \left( \widetilde{\mathbb {P}}(T_i\le u) {\mathrm {e}}^{ C(a) (c_iu)^2 t u^{-(\tau -1)} } + (1-\widetilde{\mathbb {P}}(T_i\le u)) \right) , \end{aligned}$$

which is equivalent to bounding

5.57 $$\begin{aligned}&\sum _{c_i\le 1/u} \log \left( \widetilde{\mathbb {P}}(T_i\le u) {\mathrm {e}}^{ C(a) (c_iu)^2 t u^{-(\tau -1)} } + (1-\widetilde{\mathbb {P}}(T_i\le u)) \right) \nonumber \\&\quad \le \sum _{c_i\le 1/u} \widetilde{\mathbb {P}}(T_i\le u) \left( {\mathrm {e}}^{ C(a) (c_iu)^2 t u^{-(\tau -1)} } - 1 \right) \end{aligned}$$

appropriately. Here we used that $\log (1+x) \le x$ for $x \ge 0$. Next bound $\widetilde{\mathbb {P}}(T_i\le u) \le C c_iu$ in the above to obtain that for $c_i\le 1/u$ we have $C(a) (c_iu)^2 t u^{-(\tau -1)} \le C(a,T) u^{-(\tau -1)} \le \log (2)$ for *u* big enough. Hence we can use that ${\mathrm {e}}^x-1 \le 2x$ for $0 \le x \le \log (2)$ and thus get as a further upper bound to (5.57)

5.58 $$\begin{aligned} C(a,T) \sum _{c_i\le 1/u} c_iu (c_iu)^2 u^{-(\tau -1)} \le C(a,T). \end{aligned}$$

The last inequality follows from (5.31). This completes the proof of (5.46). $\square$

**Step 1(b): Asymptotic factorization** We next show that

5.59 $$\begin{aligned} \widetilde{\mathbb {E}}\big [{\mathrm {e}}^{\sum _{j\in \mathcal {J}(u)} r_{j,t}^u}{\mathrm {e}}^{{\mathrm {i}}k u^{-(\tau -3)/2} \mathcal {S}_u}\big ] = {\mathrm {e}}^{\widetilde{\mathbb {E}}[\sum _{j\in \mathcal {J}(u)} r_{j,t}^u]} \widetilde{\mathbb {E}}\big [{\mathrm {e}}^{{\mathrm {i}}k u^{-(\tau -3)/2} \mathcal {S}_u}\big ] + o(1). \end{aligned}$$

To prove (5.59), we note that, by the definition of $r_{j,t}^u$ in (5.42),

5.60 $$\begin{aligned}&\bar{E}_u(t) \equiv \left| \widetilde{\mathbb {E}}\big [{\mathrm {e}}^{\sum _{j\in \mathcal {J}(u)} r_{j,t}^u}{\mathrm {e}}^{{\mathrm {i}}k u^{-(\tau -3)/2} \mathcal {S}_u}\big ] - {\mathrm {e}}^{\widetilde{\mathbb {E}}\left[ \sum _{j\in \mathcal {J}(u)} r_{j,t}^u\right] } \widetilde{\mathbb {E}}\big [{\mathrm {e}}^{{\mathrm {i}}k u^{-(\tau -3)/2} \mathcal {S}_u}\big ] \right| \nonumber \\&\quad = \left| \prod _{j\ge 2} \widetilde{\mathbb {E}}\big [ {\mathrm {e}}^{\mathcal {I}_j(u) r_{j,t}^u + {\mathrm {i}}k u^{-(\tau -3)/2} c_j \mathcal {I}_j(u)} \big ] - \prod _{j\ge 2} {\mathrm {e}}^{r_{j,t}^u \widetilde{\mathbb {P}}(T_j \le u)} \widetilde{\mathbb {E}}\big [ {\mathrm {e}}^{{\mathrm {i}}k u^{-(\tau -3)/2} c_j \mathcal {I}_j(u)} \big ] \right| . \end{aligned}$$

As in the calculations of the Laplace transform of $A_u$ in (5.14), we now apply (5.13). Note that here we cannot apply the second bound of (5.13) as $\sup _j (|a_j| \vee |b_j|)$ is not bounded by 1 (recall that $r_{j,t}^u \ge 0$). Instead, we get

5.61 $$\begin{aligned}&\bar{E}_u(t) \le \sum _{j \ge 2} \prod _{2\le j_1\le j-1} \left| \widetilde{\mathbb {E}}\big [ {\mathrm {e}}^{\mathcal {I}_{j_1}(u) r_{j_1,t}^u + {\mathrm {i}}k u^{-(\tau -3)/2} c_{j_1} \mathcal {I}_{j_1}(u)} \big ] \right| \nonumber \\&\quad \times \left| \widetilde{\mathbb {E}}\big [ {\mathrm {e}}^{\mathcal {I}_j(u) r_{j,t}^u + {\mathrm {i}}k u^{-(\tau -3)/2} c_j \mathcal {I}_j(u)} \big ] - {\mathrm {e}}^{r_{j,t}^u\widetilde{\mathbb {P}}(T_j \le u)} \widetilde{\mathbb {E}}\big [ {\mathrm {e}}^{{\mathrm {i}}k u^{-(\tau -3)/2} c_j \mathcal {I}_j(u)} \big ] \right| \nonumber \\&\quad \times \prod _{j_2\ge j+1} \left| {\mathrm {e}}^{r_{j_2,t}^u\widetilde{\mathbb {P}}(T_{j_2} \le u)} \widetilde{\mathbb {E}}\big [ {\mathrm {e}}^{{\mathrm {i}}k u^{-(\tau -3)/2} c_{j_2} \mathcal {I}_{j_2}(u)} \big ] \right| . \end{aligned}$$

We proceed to prove that the first and the third product are bounded by constants. Indeed, we can bound the third product using (5.7) by

5.62 $$\begin{aligned} \prod _{j_2\ge j+1} {\mathrm {e}}^{r_{j_2,t}^u\widetilde{\mathbb {P}}(T_{j_2} \le u)} \le {\mathrm {e}}^{C\sum _{j \ge 2} r_{j,t}^u (c_j u\wedge 1)}, \end{aligned}$$

where, by (5.44) and (5.31),

5.63 $$\begin{aligned} \sum _{j \ge 2} r_{j,t}^u (c_j u\wedge 1)\le C. \end{aligned}$$

For the first product in (5.61), we obtain as an upper bound

5.64 $$\begin{aligned}&\prod _{j\ge 2} \widetilde{\mathbb {E}}\big [ {\mathrm {e}}^{\mathcal {I}_j(u) r_{j,t}^u} \big ] = \prod _{j\ge 2} \left( \widetilde{\mathbb {P}}(T_j \le u) {\mathrm {e}}^{r_{j,t}^u} + (1-\widetilde{\mathbb {P}}(T_j \le u)) \right) \nonumber \\&\quad = \prod _{j\ge 2} \left( 1+\widetilde{\mathbb {P}}(T_j \le u)({\mathrm {e}}^{r_{j,t}^u}-1)\right) \le {\mathrm {e}}^{\sum _{j \ge 2} \widetilde{\mathbb {P}}(T_j \le u) ({\mathrm {e}}^{r_{j,t}^u}-1)}. \end{aligned}$$

As $r_{j,t}^u$ is uniformly bounded for *u* big enough the above is again bounded by (5.63).

Hence, it suffices to bound the middle part of (5.61), that is, it remains to show that

5.65 $$\begin{aligned}&\sum _{j \ge 2} \left| \widetilde{\mathbb {E}}\big [ {\mathrm {e}}^{\mathcal {I}_j(u) r_{j,t}^u + {\mathrm {i}}k u^{-(\tau -3)/2} c_j \mathcal {I}_j(u)} \big ] - {\mathrm {e}}^{r_{j,t}^u\widetilde{\mathbb {P}}(T_j \le u)} \widetilde{\mathbb {E}}\big [ {\mathrm {e}}^{{\mathrm {i}}k u^{-(\tau -3)/2} c_j \mathcal {I}_j(u)} \big ] \right| \nonumber \\&\quad =\Delta _j\left( r_{j,t}^u\right) = o(1), \end{aligned}$$

where we recall the definition of $\Delta _j(q)$ in (5.15). By (5.19),

5.66 $$\begin{aligned} \Delta _j\left( r_{j,t}^u\right) \le C r_{j,t}^u {\mathrm {e}}^{r_{j,t}^u}\left( r_{j,t}^u+|k| u^{-(\tau -3)/2} c_j\right) \end{aligned}$$

for $u=u(k)$ big enough. The bound on $r_{j,t}^u$ in (5.44) is equal to *C*(*a*, *T*) times the bounds on $q_j(u)$ in (5.6). The remaining calculations for $A_u$ in (5.19)–(5.21) therefore directly carry over, so that (5.65) follows. $\square$

**Step 2: The limit of** $\mathbb {E}[\sum _{j\in \mathcal {J}(u)} r_{j,t}^u ]$. In this step, we identify the limit of $\mathbb {E}[\sum _{j\in \mathcal {J}(u)} r_{j,t}^u ]$. For this, we use that by definition of $r_{j,t}^u$ in (5.42), that of $p_{j,t}^u$ in (5.41), and (2.30) with $t=u$,

5.67 $$\begin{aligned} \widetilde{\mathbb {E}}\Big [ \sum _{j\in \mathcal {J}(u)} r_{j,t}^u \Big ]&=\sum _{j \ge 2} ({\mathrm {e}}^{-a c_ju}-1+a c_ju) \frac{{\mathrm {e}}^{c_jtu^{-(\tau -2)}}-1}{1-{\mathrm {e}}^{-c_ju}} {\mathrm {e}}^{-c_ju} \frac{{\mathrm {e}}^{\theta c_ju}\left( 1-{\mathrm {e}}^{-c_ju}\right) }{{\mathrm {e}}^{\theta c_ju}\left( 1-{\mathrm {e}}^{-c_ju}\right) +{\mathrm {e}}^{-c_ju}} \nonumber \\&= tu^{-(\tau -1)} \sum _{j \ge 2} \left( {\mathrm {e}}^{-a c_ju}-1+a c_ju\right) \frac{{\mathrm {e}}^{c_ju tu^{-(\tau -1)}}-1}{t u^{-(\tau -1)}} {\mathrm {e}}^{-c_ju} \nonumber \\&\quad \frac{{\mathrm {e}}^{\theta c_ju}}{{\mathrm {e}}^{\theta c_ju}\left( 1-{\mathrm {e}}^{-c_ju}\right) +{\mathrm {e}}^{-c_ju}} \nonumber \\&\quad {\mathop {\rightarrow }\limits ^{u \rightarrow \infty }} t\int _0^\infty \left( {\mathrm {e}}^{-a x^{-\alpha }}-1+a x^{-\alpha }\right) x^{-\alpha } {\mathrm {e}}^{-x^{-\alpha }} \nonumber \\&\quad \frac{{\mathrm {e}}^{\theta x^{-\alpha }}}{{\mathrm {e}}^{\theta x^{-\alpha }}\left( 1-{\mathrm {e}}^{-x^{-\alpha }}\right) +{\mathrm {e}}^{-x^{-\alpha }}} dx. \end{aligned}$$

The convergence of the sum to the integral follows as in (5.22). Next set $-x^{-\alpha }=z$ to get

5.68 $$\begin{aligned} \lim _{u \rightarrow \infty } \frac{1}{t} \widetilde{\mathbb {E}}\left[ \sum _{j\in \mathcal {J}(u)} r_{j,t}^u\right] = \int _{-\infty }^0 ({\mathrm {e}}^{a z}-1-a z) \Pi (dz), \end{aligned}$$

with $\Pi (dz)$ as in (5.38), respectively, (2.55). For $\Pi$ to be the Lévy measure of a real-valued Lévy process with no positive jumps as in [5, Sect. V.1], by the Lévy-Khintchine formula in [5, Sect. 0.2 and Theorem 1 in Sect. I.1], we have to check that $\Pi$ is a measure on $(-\infty ,0)$ that satisfies $\int \Pi (dz) (1 \wedge z^2) < \infty$. Indeed, close to 0, $z^2 \Pi (dz)$ behaves like $(\tau -1) z^{-(\tau -3)} dz$, which is integrable at 0 and for $z \rightarrow \infty$, $\Pi (dz)$ behaves like ${\mathrm {e}}^{-z} (\tau -1) z^{-(\tau -1)} dz$, whose integral is finite for all $n \in \mathbb {N}$. $\square$

**Step 3: Completion of the proof** The convergence of $\widetilde{\mathbb {E}}\big [{\mathrm {e}}^{{\mathrm {i}}k u^{-(\tau -3)/2} \mathcal {S}_u}\big ]\rightarrow {\mathrm {e}}^{-k^2 I_{\scriptscriptstyle V}(1)/2}$ is already proved in (5.23). Therefore, Steps 1(a)–1(b) and 2, together with (5.23), complete the proof of pointwise convergence in Lemma 5.4. $\square$

To show that the dominated convergence theorem can be applied, it again remains to show that the integrand has an integrable dominating function:

##### Lemma 5.5

(Domination by an integrable function).

5.69 $$\begin{aligned} \int _{-\infty }^\infty \sup _{u \ge u_0} \left| \widetilde{\mathbb {E}}\big [\psi _{\scriptscriptstyle \mathcal {J}}(a){\mathrm {e}}^{{\mathrm {i}}k u^{-(\tau -3)/2}\mathcal {S}_u}\big ] \right| dk < \infty . \end{aligned}$$

##### Proof

This follows in a similar way as in the proof of Lemma 5.2. We compute

5.70 $$\begin{aligned} \Big |\widetilde{\mathbb {E}}\big [\psi _{\scriptscriptstyle \mathcal {J}}(a){\mathrm {e}}^{{\mathrm {i}}k u^{-(\tau -3)/2}\mathcal {S}_u}\big ]\Big |^2&=\Big |\widetilde{\mathbb {E}}\big [{\mathrm {e}}^{{\mathrm {i}}k u^{-(\tau -3)/2}\mathcal {S}_u} \prod _{j\in \mathcal {J}(u)} {\mathrm {e}}^{a c_ju p_{j,t}^u} \big (1+({\mathrm {e}}^{-a c_ju}-1)p_{j,t}^u\big )\big ]\Big |^2 \nonumber \\&=\prod _{j\ge 2}\Big |1-\widetilde{\mathbb {P}}(T_j \le u) +{\mathrm {e}}^{{\mathrm {i}}k u^{-(\tau -3)/2}c_j}\widetilde{\mathbb {P}}(T_j \le u){\mathrm {e}}^{a c_ju p_{j,t}^u} \nonumber \\&\big (1+({\mathrm {e}}^{-a c_ju}-1)p_{j,t}^u\big )\Big |^2. \end{aligned}$$

This is identical to the bound appearing in (5.25), apart from the fact that the term $e^{-aq_j(u)}$ in (5.25) is replaced with $b_{j,t}(u)={\mathrm {e}}^{a c_ju p_{j,t}^u}\big (1+({\mathrm {e}}^{-a c_ju}-1)p_{j,t}^u\big )$ in the above. Proceeding as in (5.25) to (5.28), we finally obtain

5.71 $$\begin{aligned}&\log \left| \widetilde{\mathbb {E}}\Big [\psi _{\scriptscriptstyle \mathcal {J}}(a){\mathrm {e}}^{{\mathrm {i}}k u^{-(\tau -3)/2} \mathcal {S}_u} \Big ]\right| ^2 \nonumber \\&\quad \le \sum _{j \ge 2} 2 \widetilde{\mathbb {P}}(T_j \le u) \Big [\widetilde{\mathbb {P}}(T_j \le u)((b_{j,t}(u))^2-1)/2+ \big (\cos (k u^{-(\tau -3)/2} c_j)b_{j,t}(u)-1\big ) \nonumber \\&\quad \times (1-\widetilde{\mathbb {P}}(T_j \le u)), \end{aligned}$$

where the additional first term in comparison to (5.27) arises because $b_{j,t}(u)\le 1$ no longer holds. Indeed, since ${\mathrm {e}}^{xp}(1+({\mathrm {e}}^{-x}-1)p)\ge 1$ for $x\ge 0$ and $p\in [0,1]$, we have that $b_{j,t}(u)\ge 1$. Further,

5.72 $$\begin{aligned} b_{j,t}(u)={\mathrm {e}}^{a c_ju p_{j,t}^u}\big (1+({\mathrm {e}}^{-a c_ju}-1)p_{j,t}^u\big ) \le {\mathrm {e}}^{({\mathrm {e}}^{-a c_ju}-1+a c_ju) p_{j,t}^u}={\mathrm {e}}^{r_{j,t}(u)}. \end{aligned}$$

The first part of the sum in (5.71) can, by (5.72) and since ${\mathrm {e}}^x-1 \le 2x$ for $0 \le x \le \log (2)$, be bounded by

5.73 $$\begin{aligned}&\sum _{j \ge 2} \widetilde{\mathbb {P}}(T_j \le u)^2\left( {\mathrm {e}}^{2 r_{j,t}^u}-1\right) \nonumber \\&\quad \le C(a,T)\sum _{j \ge 2}(1\wedge c_ju)^2 r_{j,t}^u. \end{aligned}$$

Now we can apply (5.44) and (5.31) to get as a further bound

5.74 $$\begin{aligned} C(a,T) \left\{ \sum _{j \ge 2: c_j>1/u} u^{-(\tau -1)} + \sum _{j \ge 2: c_j\le 1/u} c_j^3 u^{-(\tau -4)} \right\} \le C. \end{aligned}$$

For the second part of the sum (5.71), we proceed as in (5.27)–(5.34) to split it as

5.75 $$\begin{aligned}&-\sum _{j \ge 2} 2 \widetilde{\mathbb {P}}(T_j \le u) \widetilde{\mathbb {P}}(T_j \le u)\big (1-\cos (k u^{-(\tau -3)/2} c_j)\big )(1-\widetilde{\mathbb {P}}(T_j \le u)) \nonumber \\&\quad +\sum _{j \ge 2} \widetilde{\mathbb {P}}(T_j \le u) \cos (k u^{-(\tau -3)/2} c_j)(b_{j,t}(u)-1)(1-\widetilde{\mathbb {P}}(T_j \le u)). \end{aligned}$$

By a second order Taylor expansion and the fact that $r_{j,t}(u)$ is bounded, there exists a constant *C* such that $b_{j,t}(u)-1\le C r_{j,t}(u).$ Now we can proceed as in (5.27)–(5.34), where we again take advantage of being able to dominate the bounds on $r_{j,t}^u$ in (5.44) by the bounds on $q_j(u)$ in (5.6). Integrability of $| \widetilde{\mathbb {E}}[\psi _{\scriptscriptstyle \mathcal {J}}(a) {\mathrm {e}}^{{\mathrm {i}}k u^{-(\tau -3)/2} \mathcal {S}_u} ] |$ against *k* follows. $\square$

**Proof of Proposition** 5.3. The claim follows from Lemmas 5.4, 5.5 and the dominated convergence theorem. $\square$

#### Convergence of the Finite-Dimensional Distributions of $B_u$

In this section, the convergence of the one-dimensional marginals of the process $(B_u(tu^{-(\tau -2)}))_{t\ge 0}$ gets extended to convergence of its finite-dimensional distributions. In the same way as above, it can be shown that, for $0<t_1\cdots <t_n$, the increments $(B_u(t_iu^{-(\tau -2)})-B_u(t_{i-1}u^{-(\tau -2)}))_{i=1}^n$ (where, by convention, $t_0=0$) converge in distribution, under $\widetilde{\mathbb {P}}_v$, to *independent* Lévy random variables with the correct distribution.

In what follows, we only outline some minor changes in the proof. Instead of (5.40), we fix $n \in \mathbb {N}$, $\mathbf {a} \in (\mathbb {R}^+)^n$ and $0 = t_0< t_1< \cdots < t_n \le T$ and consider

5.76 $$\begin{aligned}&\psi _{\scriptscriptstyle {\mathcal J}}(\mathbf {a}) \equiv \widetilde{\mathbb {E}}\Big [{\mathrm {e}}^{-\sum _{k=1}^n a_k \left( B_u(t_k u^{-(\tau -2)})-B_u(t_{k-1} u^{-(\tau -2)}) \right) }\mid {\mathcal J}(u)\Big ] \nonumber \\&\quad = \prod _{j\in {\mathcal J}(u)} {\mathrm {e}}^{c_ju \sum _{k=1}^n a_k p_{j,t_{k-1},t_k}^u} \left( 1+\sum _{k=1}^n ({\mathrm {e}}^{-a_k c_ju}-1) p_{j,t_{k-1},t_k}^u \right) \end{aligned}$$

with (the two-point analogue to (5.43))

5.77 $$\begin{aligned}&p_{j,s,t}^u \equiv \widetilde{\mathbb {P}}\left( T_j\in (u-tu^{-(\tau -2)},u-su^{-(\tau -2)}] \mid T_j\le u\right) \nonumber \\&\quad = {\mathrm {e}}^{-c_ju} \frac{{\mathrm {e}}^{c_jtu^{-(\tau -2)}}-{\mathrm {e}}^{c_jsu^{-(\tau -2)}}}{1-{\mathrm {e}}^{-c_ju}} \end{aligned}$$

for $0 \le s \le t \le T$, using (4.8). Then, clearly, (5.43) is replaced with

5.78 $$\begin{aligned} p_{j,s,t}^u \le C (t-s) u^{-(\tau -1)} ({\mathrm {e}}^{-c_ju} c_j u \wedge 1). \end{aligned}$$

We follow Steps 1(a)–(b) to Step 3 in the proof of convergence of the one-time marginal.

Similarly to Step 1(a), one can show that

5.79 $$\begin{aligned} \widetilde{\mathbb {E}}\Big [ \Big | \psi _{\mathcal J}(\mathbf {a}) - \prod _{j\in {\mathcal {J}}(u)} {\mathrm {e}}^{\sum _{k=1}^n ({\mathrm {e}}^{-a_k c_ju}-1+a_k c_ju) p_{j,t_{k-1},t_k}^u} \Big | \Big ] = o(1). \end{aligned}$$

We then continue to reason as from (5.39) onwards, where $r_{j,t}^u$ in (5.42) gets replaced by

5.80 $$\begin{aligned} r_{j,\mathbf {t}}^u \equiv \sum _{k=1}^n \left( {\mathrm {e}}^{-a_k c_ju}-1+a_k c_ju\right) p_{j,t_{k-1},t_k}^u. \end{aligned}$$

The remaining calculations are analogous to the one-dimensional case. The asymptotic factorization in Step 1(b) is replaced with

5.81 $$\begin{aligned} \widetilde{\mathbb {E}}\big [{\mathrm {e}}^{\sum _{j\in {\mathcal J}(u)} r_{j,\mathbf {t}}^u}{\mathrm {e}}^{{\mathrm {i}}k u^{-(\tau -3)/2} \mathcal {S}_u}\big ] = {\mathrm {e}}^{\widetilde{\mathbb {E}}[\sum _{j\in {\mathcal J}(u)} r_{j,\mathbf {t}}^u]} \widetilde{\mathbb {E}}\big [{\mathrm {e}}^{{\mathrm {i}}k u^{-(\tau -3)/2} \mathcal {S}_u}\big ] + o(1) \end{aligned}$$

and we calculate the limit of $\widetilde{\mathbb {E}}[ \sum _{j\in {\mathcal J}(u)} r_{j,\mathbf {t}}^u]$ in a similar way as in Step 2 in the previous subsection as

5.82 $$\begin{aligned} \widetilde{\mathbb {E}}\Big [ \sum _{j\in {\mathcal J}(u)} r_{j,\mathbf {t}}^u\Big ]&= \sum _{j \ge 2} \sum _{k=1}^n ({\mathrm {e}}^{-a_k c_ju}-1+a_k c_ju) \frac{{\mathrm {e}}^{c_jt_ku^{-(\tau -2)}}-{\mathrm {e}}^{c_jt_{k-1}u^{-(\tau -2)}}}{1-{\mathrm {e}}^{-c_ju}} \nonumber \\&{\mathrm {e}}^{-c_ju} \widetilde{\mathbb {P}}( j \in \mathcal {J}(u) ) \nonumber \\&{\mathop {\rightarrow }\limits ^{u \rightarrow \infty }} \int _0^\infty \sum _{k=1}^n ({\mathrm {e}}^{-a_k x^{-\alpha }}-1+a_k x^{-\alpha }) x^{-\alpha } (t_k-t_{k-1}) {\mathrm {e}}^{-x^{-\alpha }} \nonumber \\&\frac{{\mathrm {e}}^{\theta x^{-\alpha }}}{{\mathrm {e}}^{\theta x^{-\alpha }}\left( 1-{\mathrm {e}}^{-x^{-\alpha }}\right) +{\mathrm {e}}^{-x^{-\alpha }}} dx \nonumber \\&= \int _{-\infty }^0 \sum _{k=1}^n \left( {\mathrm {e}}^{a_k z}-1-a_k z\right) (t_k-t_{k-1}) \Pi (dz). \end{aligned}$$

Finally, we note that

5.83 $$\begin{aligned}&\lim _{u \rightarrow \infty } {\mathrm {e}}^{\widetilde{\mathbb {E}}\left[ \sum _{j\in {\mathcal {J}}(u)} r_{j,\mathbf {t}}^u\right] } = \exp \left[ \int _{-\infty }^0 \sum _{k=1}^n ({\mathrm {e}}^{a_k z}-1-a_k z) (t_k-t_{k-1}) \Pi (dz) \right] \nonumber \\&\quad = \mathbb {E}\left[ {\mathrm {e}}^{\sum _{k=1}^n a_k (-(L_{t_k}-L_{t_{k-1}})) } \right] , \end{aligned}$$

where we have used that, by definition, Lévy processes have independent stationary increments. This completes the convergence of the finite-dimensional distributions of $(B_u(tu^{-(\tau -2)}))_{t\ge 0}$. $\square$

#### Tightness of $B_u$

We next turn to *tightness* of the process $(B_u(tu^{-(\tau -2)}))_{t\ge 0}$. For this, we use the following tightness criterion:

##### Proposition 5.6

(Tightness criterion [8, Theorem 15.6 and the comment following it]). The sequence $\{X_n\}$ is tight in $D([0,T],{\mathbb {R}}^d)$ if the limiting process *X* has a.s. no discontinuity at $t=T$ and there exist constants $C>0$, $r>0$ and $a>1$ such that for $0\le t_1<t_2<t_3\le T$ and for all *n*,

5.84 $$\begin{aligned} \mathbb {E}\Big [|X_n(t_2)-X_n(t_1)|^{r}\,|X_n(t_3)-X_n(t_2)|^{r}\Big ] \le C |t_3-t_1|^a. \end{aligned}$$

Let

5.85 $$\begin{aligned}&V^{\scriptscriptstyle (u)}(t) = B_u(tu^{-(\tau -2)})=\sum _{i\in \mathcal {J}(u)} c_iu\left[ \mathbb {1}_{\{T_i\in (u-tu^{-(\tau -2)},u]\}} \right. \nonumber \\&\quad \left. -\widetilde{\mathbb {P}}(T_i>u-tu^{-(\tau -2)} \mid T_i\le u)\right] . \end{aligned}$$

We show tightness of $V^{\scriptscriptstyle (u)}(t)$ given $u\mathcal {S}_u=v$. In what follows, we therefore bound

5.86 $$\begin{aligned}&\widetilde{\mathbb {E}}_v\left[ \left( V^{\scriptscriptstyle (u)}(t_2)-V^{\scriptscriptstyle (u)}(t_1) \right) ^2 \left( V^{\scriptscriptstyle (u)}(t_3)-V^{\scriptscriptstyle (u)}(t_2) \right) ^2 \right] \nonumber \\&= \widetilde{\mathbb {E}}\Big [ \widetilde{\mathbb {E}}\big [ ( V^{\scriptscriptstyle (u)}(t_2)-V^{\scriptscriptstyle (u)}(t_1) )^2 ( V^{\scriptscriptstyle (u)}(t_3)-V^{\scriptscriptstyle (u)}(t_2) )^2 \mid \mathcal {J}(u)\big ] \mid u\mathcal {S}_u=v\Big ]. \end{aligned}$$

First observe that with

5.87 $$\begin{aligned} \mathcal {I}_i^u(s,t) \equiv \mathbb {1}_{\{T_i\in (u-t u^{-(\tau -2)},u-s u^{-(\tau -2)}]\}} \end{aligned}$$

we have $p_{i,s,t}^u = \widetilde{\mathbb {E}}[ \mathcal {I}_i^u(s,t) \mid T_i \le u ]$ (recall (5.77)) and

5.88 $$\begin{aligned}&\widetilde{\mathbb {E}}\left[ \left( V^{\scriptscriptstyle (u)}(t_2)-V^{\scriptscriptstyle (u)}(t_1) \right) ^2 \left( V^{\scriptscriptstyle (u)}(t_3)-V^{\scriptscriptstyle (u)}(t_2) \right) ^2 \mid \mathcal {J}(u) \right] \nonumber \\&= \widetilde{\mathbb {E}}\Big [ \prod _{n\in \{1,2\}} \Big ( \sum _{i\in \mathcal {J}(u)} c_iu \left[ \mathcal {I}_i^u(t_n,t_{n+1}) - p_{i,t_n,t_{n+1}}^u \right] \Big )^2 \mid \mathcal {J}(u) \Big ]. \end{aligned}$$

By the conditional independence of the processes conditional on $\mathcal {J}(u)$ (recall comment preceding (4.8)), and as we subtract their respective expectations, we obtain

5.89 $$\begin{aligned}&\widetilde{\mathbb {E}}\left[ ( V^{\scriptscriptstyle (u)}(t_2)-V^{\scriptscriptstyle (u)}(t_1) )^2 ( V^{\scriptscriptstyle (u)}(t_3)-V^{\scriptscriptstyle (u)}(t_2) )^2 \mid \mathcal {J}(u) \right] \nonumber \\&= \widetilde{\mathbb {E}}\Big [ \sum _{i\in \mathcal {J}(u)} (c_iu)^4 \prod _{n\in \{1,2\}} \left( \mathcal {I}_i^u(t_n,t_{n+1}) - p_{i,t_n,t_{n+1}}^u \right) ^2 \mid \mathcal {J}(u) \Big ] \nonumber \\&\quad + \widetilde{\mathbb {E}}\Big [ \sum _{i\in \mathcal {J}(u)} \sum _{j\in \mathcal {J}(u) \backslash \{i\}} (c_iu)^2 (c_ju)^2 \left( \mathcal {I}_i^u(t_1,t_2) - p_{i,t_1,t_2}^u \right) ^2 \left( \mathcal {I}_j^u(t_2,t_3) - p_{j,t_2,t_3}^u \right) ^2 \mid \mathcal {J}(u) \Big ] \nonumber \\&\quad + 2 \widetilde{\mathbb {E}}\Big [ \sum _{i\in \mathcal {J}(u)} \sum _{j\in \mathcal {J}(u) \backslash \{i\}} (c_iu)^2 (c_ju)^2 \prod _{n\in \{1,2\}} \left( \left( \mathcal {I}_i^u(t_n,t_{n+1}) - p_{i,t_n,t_{n+1}}^u \right) \right. \nonumber \\&\quad \left. \left( \mathcal {I}_j^u(t_n,t_{n+1}) - p_{j,t_n,t_{n+1}}^u \right) \right) \mid \mathcal {J}(u) \Big ]. \end{aligned}$$

We can bound this from above by

5.90 $$\begin{aligned} C \left\{ \sum _{i\in \mathcal {J}(u)} (c_iu)^4 p_{i,t_1,t_2}^u p_{i,t_2,t_3}^u + \prod _{n\in \{1,2\}} \Big ( \sum _{i\in \mathcal {J}(u)} (c_iu)^2 p_{i,t_n,t_{n+1}}^u \Big ) \right\} . \end{aligned}$$

By (5.78),

5.91 $$\begin{aligned}&\widetilde{\mathbb {E}}\left[ \left( V^{\scriptscriptstyle (u)}(t_2)-V^{\scriptscriptstyle (u)}(t_1) \right) ^2 \left( V^{\scriptscriptstyle (u)}(t_3)-V^{\scriptscriptstyle (u)}(t_2) \right) ^2 \mid \mathcal {J}(u) \right] \nonumber \\&\le C (t_2-t_1) (t_3-t_2) \Big \{\sum _{i\in \mathcal {J}(u)} (c_iu)^4 u^{-2(\tau -1)} ({\mathrm {e}}^{-c_iu} c_i u \wedge 1)^2 \nonumber \\&\qquad +\Big (\sum _{i\in \mathcal {J}(u)} (c_iu)^2 u^{-(\tau -1)} ({\mathrm {e}}^{-c_iu} c_i u \wedge 1)\Big )^2 \Big \}. \end{aligned}$$

For the first sum, note that $(c_iu)^4 u^{-2(\tau -1)} =c_i^4 u^{-2(\tau -3)}$, so that its sum is order *o*(1) as $\sum _i c_i^3<\infty$ and $\tau >3$. For the second sum in (5.91), we note that the sum over *i* such that $c_i>1/u$ is clearly bounded, since it is bounded by

5.92 $$\begin{aligned} \sum _{i:c_i>1/u} (c_iu) u^{-(\tau -1)} {\mathrm {e}}^{-c_iu}, \end{aligned}$$

which converges to a constant as $u\rightarrow \infty$ since it is a Riemann approximation to a finite integral. For the contributions due to $c_i\le 1/u$, we bound its expectation as

5.93 $$\begin{aligned}&\widetilde{\mathbb {E}}_v\Big [ \Big (\sum _{i\in \mathcal {J}(u):c_i\le 1/u} (c_iu)^2 u^{-(\tau -1)} \Big )^2 \Big ] \nonumber \\&\le \sum _{i \ne j, c_i\le 1/u,c_j\le 1/u} (c_iu)^2 (c_ju)^2 u^{-2(\tau -1)} c_iu c_ju + \sum _{i\in \mathcal {J}(u):c_i\le 1/u} (c_iu)^4 u^{-2(\tau -1)} c_iu \nonumber \\&\le \Big ( \sum _{i:c_i\le 1/u} (c_iu)^3 u^{-(\tau -1)} \Big )^2 + \sum _{i:c_i\le 1/u} c_i^3 \le C, \end{aligned}$$

by (5.31). Hence, we get with (5.86) and (5.91)–(5.93),

5.94 $$\begin{aligned}&\widetilde{\mathbb {E}}_v\left[ \left( V^{\scriptscriptstyle (u)}(t_2)-V^{\scriptscriptstyle (u)}(t_1) \right) ^2\left( V^{\scriptscriptstyle (u)}(t_3)-V^{\scriptscriptstyle (u)}(t_2) \right) ^2 \right] \nonumber \\&\quad \le C (t_2-t_1) (t_3-t_2) \le C (t_3-t_1)^2, \end{aligned}$$

as required. $$\square $$

#### Completion of the Proof of Proposition 2.14(b)

The convergence of the finite-dimensional distributions together with tightness yields $(B_u(tu^{-(\tau -2)}))_{t\ge 0}{\mathop {\longrightarrow }\limits ^{d}}(L_t)_{t\ge 0}$ by [8, Theorem 5.1].

## Footnotes

1 There is a typo in [7, Theorem 2.4], in which $c=\theta -ab$ should read $c=\theta =\lambda + \zeta$.

2 We take this opportunity to correct some typos in [7]. In [7, (3.76)], the term $-abti^{-\alpha }=-abtc_i$ should be replaced by $-abti^{-2\alpha }=-abtc_i^2.$ This corresponds to the choice of $\tilde{\beta }_i=(\lambda +\zeta )/(ab)-c_i^2$ here. Further, in [7, (3.79)], the product over $j\in [i-1]$ should be over $q\in [i-1]$.
